# Cost-effectiveness analysis of transcatheter aortic valve implantation for asymptomatic severe aortic stenosis across nine European countries

**DOI:** 10.1093/ehjopen/oeag115

**Published:** 2026-07-23

**Authors:** Philippe Genereux, Suzanne Baron, Helene Eltchaninoff, Lutz Frankenstein, Christophe Dubois, Tiffany Patterson, Francesco Saia, Pim Tonino, Eduardo Pinar, Christophe Wyss, Lucy Hillcoat, Heather Davies, Tom Bromilow, Reagan Davis, Pascal Candolfi, Valentina Sellitto, Stefan James, Martine Gilard

**Affiliations:** Morristown Medical Center, Gagnon Cardiovascular Institute, 100 Madison Avenue, Morristown, NJ 07960, USA; Division of Cardiology, Corrigan Minehan Heart Center, Massachusetts General Hospital, 55 Fruit Street, Boston, MA 02114, USA; Department of Cardiology, Rouen Normandy University, INSERM U1096, CHU Rouen, 1 Rue de Germont, 76031 Rouen Cedex, France; Department of Cardiology, Heidelberg University Hospital, Im Neuenheimer Feld 410, 69120 Hedeilberg, Germany; Department of Cardiovascular Medicine, University Hospital Leuven, Herestraat 49, B-3000 Leuven, Belgium; Department of Cardiovascular Sciences, KU Leuven, Campus Gasthuisberg, Herestraat 49, Box 911, B-3000 Leuven, Belgium; Guy’s and St Thomas’ NHS Foundation Trust, Westminster Bridge Road, London SE1 7EH, UK; Cardiology Unit, IRCCS Azienda Ospedaliero-Universitaria di Bologna, Policlinico di Sant'Orsola, Via Pietro Albertoni 15, 40138 Bologna, Italy; Heart Center, Catharina Hospital, Michelangelolaan 2, 5623 EJ Eindhoven, The Netherlands; Hospital Clínico Universitario Virgen de la Arrixaca, Carretera Madrid-Cartagena s/n, 30120 El Palmar (Murcia), Spain; Heart Clinic Hirslanden, Witellikerstrasse 40, 8032 Zurich, Switzerland; York Health Economics Consortium, University of York, York YO10 5NQ, UK; York Health Economics Consortium, University of York, York YO10 5NQ, UK; York Health Economics Consortium, University of York, York YO10 5NQ, UK; York Health Economics Consortium, University of York, York YO10 5NQ, UK; Edwards Lifesciences, 70 route de l'Etraz, 1260 Nyon, Switzerland; Edwards Lifesciences, 70 route de l'Etraz, 1260 Nyon, Switzerland; Department of Medical Sciences, Cardiology and Uppsala Clinical Research Center, Uppsala University, SE-751 85 Uppsala, Sweden; Department of Cardiology, Brest University Hospital (CHU Brest), Boulevard Tanguy Prigent, 29200 Brest, France

**Keywords:** Transcatheter aortic valve implantation, Transcatheter aortic valve replacement, Clinical surveillance, Asymptomatic severe aortic stenosis, Early intervention, Cost-effectiveness

## Abstract

**Aims:**

Asymptomatic severe aortic stenosis (aSAS) is associated with morbidity, with mortality increasing once symptoms develop. Benefits of early aortic valve replacement (AVR) over clinical surveillance (CS) with delayed AVR upon symptom onset have been demonstrated, and it is now recommended from the latest European Guidelines if procedural risk is low (Class IIa, Level A). The aim of this study is to estimate the economic impact of transcatheter aortic valve implantation (TAVI) for the treatment of aSAS.

**Methods and results:**

A cost-utility analysis compared TAVI using the SAPIEN 3/SAPIEN 3 Ultra valve vs. CS in patients with aSAS across nine European countries. A lifetime Markov model captured peri-procedural and long-term outcomes across three health states: alive and well, stroke, and death. Inputs were derived from the EARLY TAVR trial (NCT03042104) and literature sources. Clinical event rates were estimated via parametric survival analysis. Transcatheter aortic valve implantation treatment for patients with aSAS was modelled to be the economically dominant strategy for treating aSAS across all nine European healthcare systems, with cost-effectiveness probabilities ranging from 97.3% (UK) to 99.9% (Belgium). Incremental results per person included cost savings from −£1788 (UK) to −CHF15 802 (Switzerland), quality-adjusted life years gains of 0.18 (Germany, UK) to 0.23 (Switzerland), and life-year gains of 0.12 (UK) to 0.17 (Belgium). Annual discounting of upfront TAVI costs vs. delayed AVR costs drove results.

**Conclusion:**

For patients with aSAS, early intervention with TAVI is estimated to deliver greater health benefits and reduce costs compared with CS. These findings support policies promoting early detection and timely intervention before symptom onset.

## Graphical Abstract

**Graphical Abstract:**
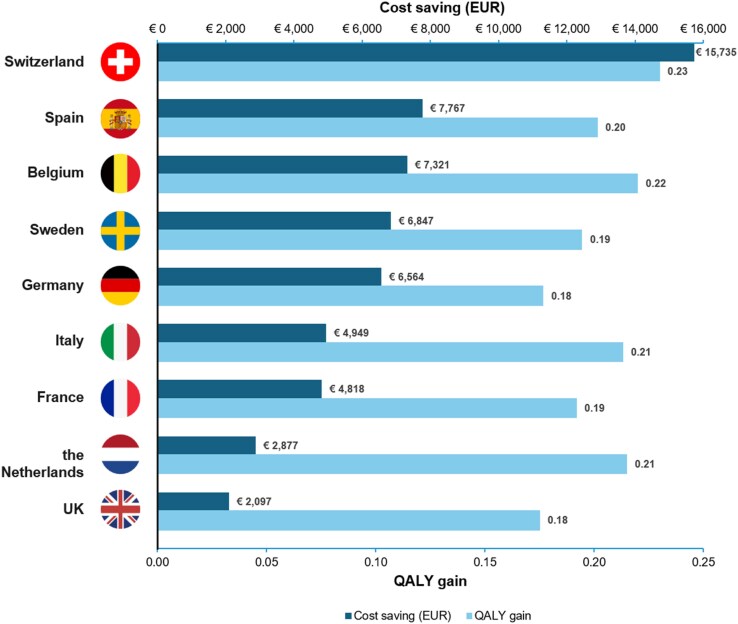

Key Learning Points *What is already known*

The European Society of Cardiology Guidelines 2025 recommend early aortic valve replacement for asymptomatic severe aortic stenosis instead of active surveillance if the procedural risk is low. This is based on trials showing a lower incidence of a composite of death, stroke, or unplanned cardiovascular-related hospitalization. The guidelines do not take health care costs into consideration.

*What this study adds*

Our study supports this new recommendation from an economical point of view, suggesting that early aortic valve replacement is cost-saving and generating more quality adjusted life years.

## Introduction

Aortic stenosis (AS) is common and affects ∼5% of adults aged over 65 globally.^1^ Aortic stenosis is known to be associated with increased morbidity and mortality, especially when AS severity increases and symptoms (i.e. dyspnoea, angina, syncope, and heart failure) develop.^2^ Aortic valve replacement (AVR), either via surgery or transcatheter aortic valve implantation (TAVI), is indicated for patients with symptomatic severe AS (sSAS). At least one-third of severe AS patients are asymptomatic (aSAS) at initial diagnosis.^3^ For those patients who also have low operative risk, clinical surveillance (CS) is recommended, with AVR when symptoms develops or left ventricular function decreases.

Recently, four randomized trials have shown the benefits of a strategy of early intervention over CS in asymptomatic populations, with several meta-analyses showing a significant reduction in heart failure hospitalizations and stroke.^4,5^ Consequently, the updated 2025 European Guidelines recommend early AVR for aSAS if the procedural risk is low (Class 2a, Level A).^6^

The EARLY TAVR (NCT03042104) trial was the largest trial with 901 patients randomized to either early intervention with TAVI [with SAPIEN 3/SAPIEN 3 Ultra valves (Edwards Lifesciences, Irvine, CA)], compared to a strategy of CS.^7^ The EARLY TAVR trial demonstrated the superiority of early TAVI compared to CS, with a ∼50% reduction of the primary endpoint [a composite of death, stroke, or unplanned cardiovascular-related hospitalization (CVH)] at a median follow-up of 3.8 years.^8^ It also demonstrated a ∼40% reduction of the composite of death, stroke, and heart failure hospitalization. Whether a strategy of early intervention with TAVI compared to CS is cost-effective among patient with severe AS and no symptoms has never been studied. Therefore, we aim to evaluate the cost-effectiveness of early TAVI in an aSAS population compared with CS in nine European countries, using data from the EARLY TAVR trial.

## Methods

The cost-effectiveness of early TAVI was modelled in nine European settings: Belgium, France, Germany, Italy, the Netherlands, Spain, Sweden, Switzerland, and the UK. Each setting reflected country-specific demographics and modelling guidelines and was modelled from a healthcare payer perspective. The discount rates and willingness-to-pay thresholds applied for each country are presented in Supplementary material online, *Table S1*.

### Model structure

**Figure 1:**
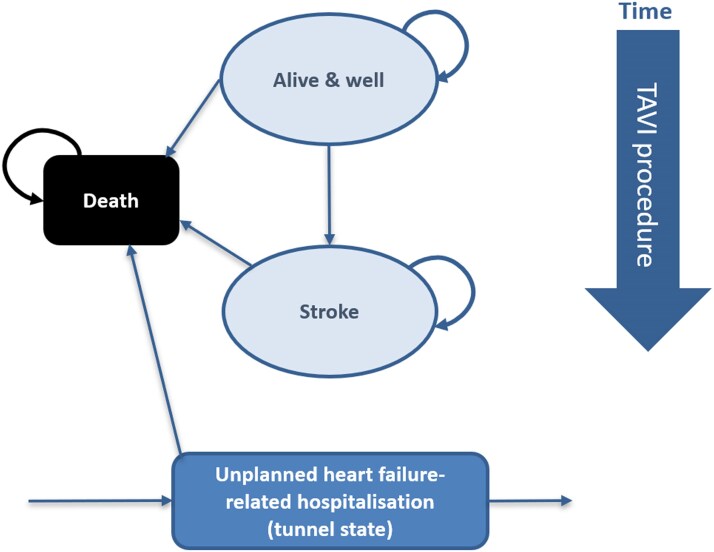
Model structure. The model consisted of three health states: alive and well, stroke, and death. Death was an absorbing state. An unplanned heart failure-related hospitalization tunnel state was used to represent cardiovascular-related hospitalization. The tunnel state could be entered each cycle from the alive-and-well or stroke health states. TAVI, transcatheter aortic valve implantation.

A Markov model structure with a 30-day cycle length was used across a lifetime time horizon to capture both the periprocedural events and long-term impacts of a TAVI procedure. The chosen methodology aligns with previously published EU TAVI cost-effectiveness models for individuals with sSAS at low surgical risk.^9–17^ The Excel-based model consisted of three health states: alive and well, stroke, and death. Death was an absorbing state and could be entered at any point. An unplanned, heart failure-related hospitalization (HFH) tunnel state was used to represent CVH. The tunnel state could be entered each cycle from the alive-and-well or stroke health states (*Figure 1*). There was no limit on the number of HFHs that could be experienced per person and a HFH could occur in the same cycle that AVR is received.

The asymptomatic cohort entered the model in the alive-and-well health state and either underwent TAVI or received CS. In the latter arm of the model, individuals only received AVR upon developing symptoms—in line with the EARLY TAVR data.^8^ For each cycle, there was a risk that the cohort could experience a disabling or non-disabling stroke and progress to the stroke health state. A simplifying assumption was made that only one stroke could be experienced per person within their lifetime. Once progressed, the cohort could not return to the alive-and-well state.

### Model inputs

#### Clinical inputs

The clinical inputs were informed by the EARLY TAVR trial where possible and were consistent across the nine models.^8^ The starting age (75.8 years) and proportion who were male (69.1%) reflected the baseline characteristics of the trial.

The TAVI arm underwent the procedure in the first model cycle; this in line with the median time to procedure (14 days).^8^ The CS arm underwent the procedure in line with time-to-event data. In this arm, 1.8% underwent SAVR rather than TAVI.^8^ The cumulative incidence of the CS arm converting to AVR was defined by symptom level [namely acute valve syndrome (AVS), progressive valve syndrome (PVS), and asymptomatic].^8,18,19^ Published Kaplan–Meier data were digitized and extrapolated beyond the 5-year trial period (see Supplementary material online, *Figure S1*).^20^ Akaike information criterion, Bayesian information criterion, and visual graphical checks were used to determine the statistically best-fitting parametric curve for the digitized dataset, which was the exponential parametric model. From EARLY TAVR, ∼87% of the CS cohort converted to AVR at the median follow-up of 3.8 years, which increased to 100% of those alive within the model’s lifetime horizon.

In line with the EARLY TAVR trial, 42.1% and 43.3% of strokes were disabling in the TAVI and CS arms, respectively.^8^ The annual stroke and HFH event probabilities reported by Généreux *et al.* were digitized using WebPlotDigitizer (v5).^8,20^ The rate of HFHs was used instead of CVHs to mitigate the risk of double counting costs; CVH data reported in EARLY TAVR included the cost of subsequent AVR within 6 months of randomization for the CS arm. It was assumed that a HFH could occur ahead of undergoing AVR in the CS arm, and the annual event probabilities are presented in *Table 1*.

**Table 1:** Key clinical inputs

	TAVI	CS	Source
Weighted of disabling stroke	42.1%	43.3%	Genereux (2025)^8^
Proportion receiving a PPM	5.7%	8.4%	Genereux (2025)^8^
Length of a HFH	7 days	7 days	Nombelo-Franco (2015)^21^
Stroke rate			
Year 1	1.32%	1.79%	Genereux (2025)^8^
Year 2	1.34%	2.05%	
Year 3	1.35%	1.86%	
Year 4	0.69%	1.90%	
Year 5+	0.92%	2.18%	
Heart failure-related hospitalization rate			
Year 1	1.32%	5.16%	Genereux (2025)^8^
Year 2	0.22%	2.84%	
Year 3	1.34%	1.46%	
Year 4	1.36%	0.99%	
Year 5+	1.38%	2.24%	

CS, clinical surveillance; HFH, heart failure-related hospitalization; PPM, new permanent pacemaker; TAVI, transcatheter aortic valve implantation.

The only periprocedural event that impacted ≥5% of the cohort was the receipt of a new permanent pacemaker (PPM), which occurred at a rate of 5.7% for the early TAVI group and 8.4% for CS group. It was assumed that a PPM would be implanted during the AVR procedure and would not impact mortality risk. Therefore, it was deemed appropriate to apply these arm-specific values to all who underwent AVR, regardless of health state.

It was assumed that the cohort could not undergo reintervention in the same cycle as AVR. One-, 2-, and 5-year outcomes from a clinical trial investigating TAVI in a low-risk sSAS population (PARTNER 3) were used to inform the probability of reintervention in the TAVI arm for the first 5 years.^22^ The rates for years 6 and 7 were assumed to be the same as year 5. Data from Bourguignon (2015) informed data from year 8 onwards, with two alternative data sources used to conduct scenario analysis.^23–25^ Model structure limitations meant that a constant, average probability of reintervention per cycle (0.12%) was applied to the CS arm. Further details are reported in the Supplementary material, and the annual probabilities are presented in Supplementary material online, *Table S5*.

#### Mortality

The survival rate in the EARLY TAVR trial was higher than the estimated all-cause mortality of the general population.^8^ Therefore, the trial data were used to conduct scenario analyses but were not used in the model base case. Instead, health state-specific relative risks were applied to general population mortality age and sex-adjusted rates for each country, in line with previous models.^9–15^ Life tables for the years 2017/2019 were used to avoid data sets that may be affected by COVID-19.^26–33^

The stroke-relative risks are presented by country in the Supplementary material (see Supplementary material online, *Table S2*). Mortality was higher during the first month following a stroke event with a lower relative risk being applied for the remainder of the time horizon. The alive-and-well state reflects an asymptomatic population and, therefore, no additional risk of mortality was applied. Furthermore, it was assumed that there would be no increase in mortality by temporarily entering the HFH tunnel state from the alive-and-well or stroke health state, given that the impact of a stroke on mortality was considered greater than experiencing a HFH.^9^

#### Costs and resource use

Cost data for each country (2022/2023) were primarily obtained from literature, including diagnostic-related groups and funding for the TAVI procedure.^9–16^ Costs were inflated to 2022 costs where necessary, using country-specific inflation indices. Although the costs differed for each country, the same resource use parameters and input categories were assumed appropriate across the nine models, with the difference in surveillance captured by the country-specific costs. The cost inputs are presented in the Supplementary material (Supplementary material online, *Table S3*).

The cost of undergoing TAVI included the cost of the SAPIEN 3/SAPIEN 3 Ultra valve and the implantation procedure; this was equal across model arms. Surgical AVR was costed as a complex procedure (rather than standard), in line with clinical expert opinion. The cost associated with a reintervention was assumed equivalent to the initial intervention in each arm.

The CS arm in the alive-and-well health state incurred a monitoring cost until the AVR procedure, consisting of one annual echocardiogram and consultation. The TAVI arm received TAVI in the first model cycle and, therefore, did not require these monitoring costs.^12^ The cost of a HFH event was incurred upon entry to the tunnel state and reflected 7 days in hospital.^9,21^

There were two costs associated with strokes. First, an initial cost was incurred in the same cycle as the event, with this value being stratified by stroke severity. Second, the disabling stroke substrate was associated with an ongoing cost that persisted for the remainder of the time horizon.

#### Utilities

Health state-specific utility adjustment factors were applied multiplicatively to country-specific, age-stratified, and sex-stratified general population norm EQ-5D values.^34–36^ The health state-specific utility values were divided by the age-specific study base case utility values to calculate the adjustment factors. The alive-and-well state was assumed equivalent to the general population. Utility values for stroke were country specific (see Supplementary material, Supplementary material online, *Table S4*). Across all countries, the non-disabling stroke utility value remained constant over the time horizon, in line with findings from a 10-year study.^37^ Although evidence suggests that health-related quality of life (HRQoL) improves following a disabling stroke, the memoryless nature of Markov models meant this could not be tracked. Instead, a constant utility from 2 months after the disabling stroke was assumed. The impact of a stroke on HRQoL was considered greater than that of an HFH. Therefore, no reduction in HRQoL was applied to the HFH tunnel state; this was intuitive for the HFH-stroke substrate but conservative for the alive-and-well HFH substrate.

### Outcomes and sensitivity analysis

The economic outcomes include the average total costs, life years, and quality-adjusted life years (QALYs) per person, as well as the incremental cost-effectiveness ratio (ICER), net monetary benefit, and net health benefit. The clinical outcomes of the analysis included the number of strokes, HFH, reinterventions, and PPM per person.

Deterministic and probabilistic sensitivity analyses (DSA and PSA) were conducted to account for model uncertainty (see Supplementary material for all DSA and PSA inputs and methods). All input values were included in the DSA with the exception of values that changed each year. These include the rate of reintervention, the general population norms for utility values and the general population mortality rate. Further, a total of 28 scenarios were conducted to account for assumptions on mortality, time horizon, reintervention rates, and event rates (see Supplementary material). One key scenario used real-world symptom level breakdown of PVS and AVS to (i) derive hazard ratios for increased rates of HFH and mortality events and (ii) reflect the likely increased cost incurred whilst undergoing AVR. The EARLY TAVR cohort reflected a sample that mostly consisted of patients with PVS (58.5%). Conversely, a real-world analysis of 17 838 comparative patients found that the majority had AVS (51.7%; PVS 34.4%; asymptomatic 14.0%), which is more severe and clinically burdensome than PVS.^19,38^ Using real-world data more closely represents the higher risk of event rates that are observed in clinical practice (see Supplementary material online, *Table S8A*–*C*). Further scenarios used data from a systematic review and meta-analysis to apply incident rate ratios for risk of advanced symptoms (all-cause mortality, HFH, and stroke; Supplementary material online, *Tables S6* and *S7*).^4^

## Results

For each country, it is estimated that early intervention with TAVI for patients with aSAS will yield a lower lifetime cost and generate more QALYs than CS (*Table 2*; Supplementary material online, *Table S9* and *Figures S2*–*S10*; *Figure 2*). As such, it is estimated that early TAVI yields a dominant cost-effectiveness profile when compared with CS: there is a >97% probability of being cost-effective in each country (see Supplementary material online, *Figures S11*–*S28*). The results indicate that early TAVI will generate the highest net health benefit in the Swiss healthcare system and will generate the lowest net health benefit in the UK healthcare system.

**Figure 2:**
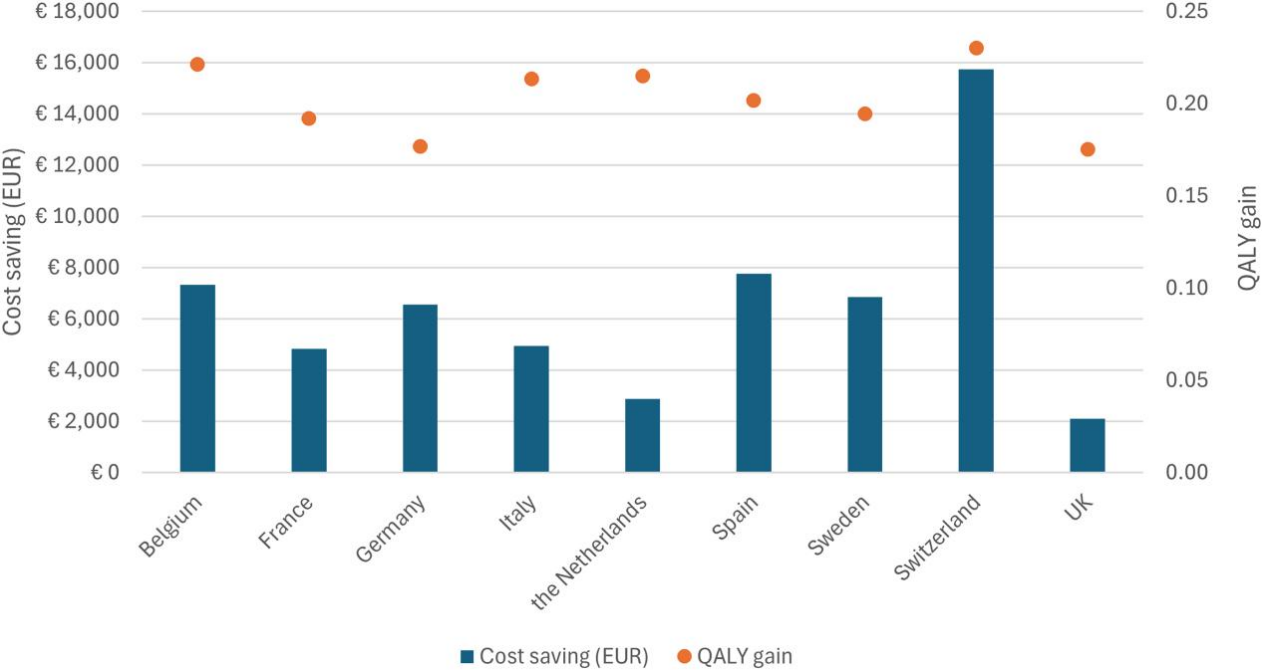
Probabilistic model results. Cost savings and quality-adjusted life year gains of early transcatheter aortic valve implantation compared with clinical surveillance. Currency for Sweden, Switzerland, and UK has been converted to Euros (2022 exchange rate: Sweden 0.094; Switzerland 0.996; UK 1.173).

**Table 2:** Probabilistic economic results by country

	Early TAVI costs (95% CI)	CS costs (95% CI)	Early TAVI QALYs (95% CI)	CS QALYs (95% CI)	Inc. costs (95% CI)	Inc. QALYs (95% CI)	ICER	% Ce	NMB (95% CI)	NHB (95% CI)
Belgium	€34 433 (€29 495 : €40 724)	€41 755 (€34 593 : €51 062)	7.97 (6.05 : 9.65)	7.75 (5.92 : 9.34)	−€7321 (−€13 778 : −€2167)	0.22 (0.04 : 0.47)	Dominant	99.94%	€13 963 (€5675 : €24 273)	0.47 (0.19 : 0.81)
Switzerland	80 325 CHF (60 255 CHF: 103 123 CHF)	96 128 CHF (73 631 CHF: 121 462 CHF)	9.11 (7.20 : 10.59)	8.88 (7.07 : 10.24)	−15 802 CHF (−30 653 CHF: −3712 CHF)	0.23 (0.03 : 0.49)	Dominant	99.34%	27 312 CHF (7672 CHF: 50 064 CHF)	0.55 (0.15 : 1.00)
Germany	€38 620 (€28 781 : €49 802)	€45 184 (€34 584 : €57 312)	8.09 (6.47 : 9.37)	7.91 (6.36 : 9.11)	−€6564 (−€11 977 : −€2215)	0.18 (0.02 : 0.39)	Dominant	99.70%	€12 751 (€4529 : €22 693)	0.36 (0.13 : 0.65)
Spain	€40 315 (€30 049 : €52 212)	€48 081 (€36 671 : €61 003)	7.56 (5.70 : 9.03)	7.36 (5.58 : 8.72)	−€7767 (−€15 099 : −€1895)	0.20 (0.04 : 0.42)	Dominant	99.18%	€13 813 (€4085 : €25 446)	0.46 (0.14 : 0.85)
France	€33 029 (€28 944 : €37 699)	€37 847 (€32 482 : €44 162)	7.54 (5.72 : 9.32)	7.35 (5.64 : 8.99)	−€4818 (−€9101 : −€1392)	0.19 (0.03 : 0.43)	Dominant	99.30%	€14 415 (€4025 : €27 870)	0.29 (0.08 : 0.56)
Italy	€47 204 (€34 355 : €62 038)	€52 154 (€39 017 : €67 037)	7.96 (5.73 : 9.58)	7.75 (5.65 : 9.25)	−€4949 (−€10 580 : −€576)	0.21 (0.03 : 0.46)	Dominant	99.54%	€11 346 (€3614 : €20 992)	0.38 (0.12 : 0.70)
Netherlands	€50 666 (€36 953 : €66 604)	€53 543 (€40 193 : €68 753)	8.92 (6.92 : 10.57)	8.70 (6.82 : 10.23)	−€2877 (−€6259 : €37)	0.21 (0.02 : 0.48)	Dominant	98.36%	€13 627 (€2687 : €28 134)	0.27 (0.05 : 0.56)
Sweden	430 583 SEK (318 926 SEK: 557 845 SEK)	503 343 SEK (386 838 SEK: 635 062 SEK)	7.37 (5.50 : 9.02)	7.18 (5.40 : 8.72)	−72 760 SEK (−124 314 SEK: −30 543 SEK)	0.19 (0.03 : 0.42)	Dominant	99.76%	267 263 SEK (88 306 SEK: (502 532 SEK)	0.27 (0.09 : 0.50)
UK	£37 464 (£34 250 : £41 044)	£39 252 (£35 683 : £43 349)	7.10 (5.32 : 8.59)	6.93 (5.24 : 8.35)	−£1788 (−£4346 : £378)	0.18 (0.03 : 0.38)	Dominant	97.32%	£5289 (£613 : £10 927)	0.26 (0.03 : 0.55)

BE, Belgium; CH, Switzerland; CI, credible interval; CS, clinical surveillance; DE, Germany; early TAVI, early transcatheter aortic valve implantation; ES, Spain; FR, France; ICER, incremental cost effectiveness ratio; Inc., incremental; IT, Italy; NHB, net health benefit; NL, Netherlands; NMB, net monetary benefit; QALY, quality-adjusted life year; SE, Sweden; UK, United Kingdom.

*Tables 3* and *4* present the estimated deterministic lifetime clinical events per person and median survival in each arm, for each country. The per-person results demonstrate that early TAVI increases median survival by 0.16–0.25 years, decreases strokes by 0.03–0.05, decreases HFH by 0.12–0.14, and decreases PPM by 0.03. Early TAVI also leads to an increase of 0.03–0.05 reinterventions per person across all countries.

**Table 3:** Clinical events in each arm by country per person (deterministic)

	Early TAVI	Clinical surveillance	Incremental
Belgium			
Total disabling strokes	0.05	0.09	−0.04
Total non-disabling strokes	0.06	0.09	−0.03
Total HFH	0.14	0.26	−0.12
Total reinterventions	0.17	0.13	0.04
Total new permanent pacemakers	0.06	0.08	−0.03
Switzerland			
Total disabling strokes	0.05	0.10	−0.05
Total non-disabling strokes	0.07	0.10	−0.03
Total HFH	0.17	0.31	−0.14
Total reinterventions	0.23	0.18	0.05
Total new permanent pacemakers	0.06	0.08	−0.03
Germany			
Total disabling strokes	0.04	0.09	−0.04
Total non-disabling strokes	0.06	0.09	−0.03
Total HFH	0.14	0.26	−0.12
Total reinterventions	0.15	0.12	0.03
Total new permanent pacemakers	0.06	0.08	−0.03
Spain			
Total disabling strokes	0.05	0.09	−0.05
Total non-disabling strokes	0.06	0.09	−0.03
Total HFH	0.15	0.28	−0.13
Total reinterventions	0.19	0.15	0.04
Total new permanent pacemakers	0.06	0.08	−0.03
France			
Total disabling strokes	0.05	0.10	−0.05
Total non-disabling strokes	0.07	0.10	−0.03
Total HFH	0.15	0.28	−0.13
Total reinterventions	0.19	0.15	0.04
Total new permanent pacemakers	0.06	0.08	−0.03
Italy			
Total disabling strokes	0.05	0.09	−0.05
Total non-disabling strokes	0.06	0.09	−0.03
Total HFH	0.15	0.27	−0.13
Total reinterventions	0.18	0.14	0.04
Total new permanent pacemakers	0.06	0.08	−0.03
Netherlands			
Total disabling strokes	0.04	0.09	−0.04
Total non-disabling strokes	0.06	0.09	−0.03
Total HFH	0.14	0.26	−0.12
Total reinterventions	0.15	0.12	0.03
Total new permanent pacemakers	0.06	0.08	−0.03
Sweden			
Total disabling strokes	0.05	0.09	−0.05
Total non-disabling strokes	0.06	0.09	−0.03
Total HFH	0.15	0.27	−0.13
Total reinterventions	0.18	0.14	0.04
Total new permanent pacemakers	0.06	0.08	−0.03
UK			
Total disabling strokes	0.04	0.09	−0.04
Total non-disabling strokes	0.06	0.09	−0.03
Total HFH	0.14	0.26	−0.12
Total reinterventions	0.17	0.13	0.03
Total new permanent pacemakers	0.06	0.08	−0.03

BE, Belgium; CH, Switzerland; DE, Germany; ES, Spain; FR, France; IT, Italy; NL, Netherlands; SE, Sweden; UK, United Kingdom.

**Table 4:** Median survival by country per person

	Early TAVI median survival	Clinical surveillance median survival	Incremental
Belgium	11.08	10.83	0.25
Switzerland	13.25	13.00	0.25
Spain	11.75	11.50	0.25
Germany	10.75	10.58	0.17
France	12.17	11.92	0.25
Italy	11.58	11.42	0.16
Netherlands	10.75	10.58	0.17
Sweden	11.58	11.33	0.25
UK	11.00	10.83	0.17

TAVI, transcatheter aortic valve implantation; UK, United Kingdom.

Supplementary material online, *Figures S29*–*S37*, present the results of the one-way sensitivity analyses for each country. Two key drivers of the results are the annual discounting applied to costs and the mortality relative risk applied to the alive-and-well health state.

The results of each respective scenario analysis are detailed within Supplementary material online, *Tables S10*–*S37*. Early TAVI was consistently cost-effective compared with CS.

## Discussion

This is the first study to assess the cost-effectiveness of early TAVI among people with aSAS in Europe. Early TAVI in this population was found to be dominant compared with CS over a lifetime horizon in all countries analysed. These findings are robust, with results from PSA indicating >97% probability of cost-effectiveness for all countries. Consistent findings were observed across a variety of scenario analyses.

Prior studies have established the cost-effectiveness of TAVI in patients with sSAS across nine European countries, reporting it to be cost-effective over SAVR in patients with low-risk surgical mortality.^9–16^ This study adds to the existing literature, demonstrating early TAVI to have economic dominance (i.e. saving costs and generating more QALYs) over CS for the same nine countries. While the magnitude of dominance varies across countries, this is expected. It is attributable to the country-specific model parameterization, in areas such as procedure cost, complication cost, survival, and HRQoL. While the early TAVI arm saw higher incremental costs associated with AVR procedures and reinterventions, there was both a cost saving and health benefit associated with fewer incremental number of strokes and HFH, leading to the dominant ICER result.

The clinical dominance in this study is mainly driven by fewer strokes, HFH and PPMs occurring in the early TAVI arm. This is expected because elective intervention in the aSAS population is likely to reduce the probability of negative clinical events typically associated with sSAS, such as HFH. A recent study demonstrated that patient’s clinical presentation with AVS (such as severe symptoms such as NYHA 3–4 heart failure or syncope) or PVS (mild symptoms such as NYHA 2 heart failure) before AVR was associated with an increase in 36 000 US dollars and ∼27 000 US dollars, respectively, 1-year post-AVR compared to patients treated while asymptomatic.^39^ Additionally, longer surveillance time allowed for the AS severity to progress and to become more complex to treat, with progression of aortic valve calcification, increasing the risk for stroke either in the waiting period (micro-embolism), or peri-procedurally. Consequently, the savings associated with a reduced lifetime incidence of HFHs, strokes, and PPMs offset the high upfront cost of early TAVI.

The one-way sensitivity analyses indicate that the relative risk for mortality in the alive-and-well state is the most common key driver of results. This is predominantly due to a high proportion of the early TAVI arm remaining in the alive-and-well health state due to the early AVR. A change in this parameter will substantially impact the rate of mortality within the early TAVI arm, which will markedly affect the overall cost-effectiveness profile. Another key driver is the annual discounting factor for costs, which is a consequence of the lifetime model horizon. Costs incurred in later years are subject to substantial cumulative discounting. Therefore, the discount factor applied will have a considerable influence on their present value. However, as shown in the tornado graphs (see Supplementary material), the results for all countries remain cost-effective (a positive NMB) when the discount rates are varied according to country-specific guidelines.

Scenario analyses were conducted to evaluate the impact of changing key modelling assumptions and parameter estimates on the model results and overall cost-effectiveness conclusion. For example, the EARLY TAVR trial results may not be reflective of real-world clinical practice in terms of the symptom-level breakdown of participants, mortality rates and the cost of an AVR procedure. Several scenarios were run with alternative real-world data that is deemed more reflective of clinical practice. Early TAVI remained cost-effective across all scenarios, supporting that it is a cost-effective alternative to CS in an aSAS population.

As this cost-effectiveness analysis is primarily informed by EARLY TAVR,^7,8^ the results are likely to be conservative because the CS in this randomized controlled setting was more rigorous than in real-world clinical practice. In the CS arm of EARLY TAVR, the median time from presenting with AS symptoms to receiving an AVR procedure was 32 days.^8^ However, this timeframe is expected to be longer in real-world clinical practice. The cost of the TAVI procedure was conservatively assumed to be equal in both model arms. In clinical practice, it is likely that a TAVI procedure would cost more under a CS strategy as patients would be symptomatic and, thus, at a higher risk of procedural complications. This would require more extensive mitigation procedures compared to asymptomatic patients, which increases cost.

Previous literature and recent guidelines have shown that early elective procedures can have more beneficial outcomes than waiting until symptoms develop, emphasizing that identifying people with aSAS is crucial for timely intervention.^6,40,41^ The findings of this analysis, estimating that early TAVI is cost-effective when compared with CS in nine European countries, aligns with previous literature that early intervention strategies in cardiovascular disease are often cost-effective.^42^

Further, based on ∼96 000 people with aSAS across Europe being eligible for early TAVI a year and an average lifetime cost saving of over €6000 per person (based on this analysis and Durko *et al.*), early TAVI has the potential to save over €500 million across Europe for each annual cohort of patients.^3^ Whilst healthcare capacity issues may lead to this to being an overestimate, it indicates how valuable early TAVI could be.

### Strengths and limitations

A key strength of the methodology in this economic analysis is that, where possible, evidence was used from EARLY TAVR, a large and contemporary RCT.^7,8^ This source provides robust clinical evidence to inform the efficacy data for early TAVI as an intervention for adults with aSAS. The models reflect the clinical trial in several aspects, including demographic characteristics, clinical and periprocedural event rates, mortality data, and pre- and post-procedure impact on HRQoL.

Additionally, unit costs in the model were sourced from national, publicly available databases or from published literature and were inflated to the current cost year where necessary. This makes it probable that costs are accurately captured for each of the healthcare perspectives modelled. The starting age of the cohort was 75.8 years and therefore productivity costs were not modelled, which is a conservative limitation because, by treating aSAS earlier, the steep decline in HRQoL and associated impact on productivity would be mitigated.

There are limitations of the current analysis that should be considered. Namely, the models face the same limitations as EARLY TAVR. The trial participants were aged ≥65 years, predominantly white, eligible for transfemoral TAVI, at low surgical risk, and received a balloon-expandable valve (SAPIEN 3/SAPIEN 3 Ultra valves). Therefore, the results of these cost-effectiveness analyses are not generalizable to a population with different key characteristics, or patients receiving TAVI via a non-transfemoral approach with a different valve platform. Similarly, the EARLY TAVR trial was conducted in the United States and Canada. It is assumed that the outcomes are generalizable across Europe. Further, in the CS arm, the time to AVR procedure and the proportion undergoing SAVR were informed by the trial for each model, whereas these aspects may vary by country in real-world practice. Clinical differences across countries are to be expected in real-world practice, but the trial outcomes for the CS arm will reflect the time to AVR procedure as seen in the trial (a median of 32 days). In reality, due to healthcare constraints, it may be that the time to AVR procedure is longer, which could lead to more favourable outcomes for the early TAVI arm.

A key limitation is that mortality data from the EARLY TAVR trial was not used to generate the base-case results. This was because the results indicated that the survival rate was higher than the estimated all-cause mortality of the general population.^8^ Scenarios were run utilizing the EARLY TAVR trial mortality data, both with and without the general population adjustment. The findings of these scenario analyses are consistent with the base-case, indicating that a strategy of early TAVI is cost-effective when compared with CS.

A further limitation comes from the memoryless nature of Markov models and the varying time point at which those in the CS arm received AVR, meaning that only the cumulative proportion that converted to AVR could be calculated over time. The amount of time that had passed following AVR for each individual in the CS arm could not be tracked. Therefore, time-dependent inputs, such as the probability of reintervention each year following the initial AVR procedure, could not be applied directly. Instead, a static monthly probability of reintervention was calculated and applied in each cycle, based on a weighted average of the probability of intervention at each time point and the proportion alive at each time point.

Another limitation comes from the assumption that the model cohort can experience only one stroke in their lifetime, which may not be reflective of clinical practice. The results from EARLY TAVR reported that some individuals experienced more than one stroke in the CS arm, but this was not observed in the TAVI arm.^7,8^ Therefore, the approach taken does not favour early TAVI and is deemed conservative.

Lastly, the long-term durability of the pericardial bioprosthesis was based on currently available reintervention rates in the literature, most of which were observed within an intermediate and high-surgical risk population. As such, there remains a possibility that reintervention rates could be higher in a low-risk population. This is because they would be expected to live longer than the high-risk population, which could lead to greater costs in the early TAVI arm. Because of this, the most conservative estimates were selected to inform reintervention rates in the base case.

## Conclusions

Early TAVI is the dominant treatment option for individuals with aSAS in all healthcare systems evaluated within this analysis. These findings support policies and the growing body of evidence promoting the early detection of AS and timely intervention before symptom onset, as opposed to providing CS with a delayed AVR procedure.^41^

## Supplementary Material

oeag115_Supplementary_Data

## Data Availability

Input parameters values used and data generated during this cost-utility study are wholly included within this article and the associated Supplementary material.

## References

[1] Genereux P, Stone GW, O'Gara PT, Marquis-Gravel G, Redfors B, Giustino G, Pibarot P, Bax JJ, Bonow RO, Leon MB. Natural history, diagnostic approaches, and therapeutic strategies for patients with asymptomatic severe aortic stenosis. J Am Coll Cardiol 2016;67:2263–2288. 27049682 10.1016/j.jacc.2016.02.057

[2] Pujari SH, Agasthi P. Aortic Stenosis. StatPearls: StatPearls Publishing; 2025. 32491560

[3] Durko AP, Osnabrugge RL, Van Mieghem NM, Milojevic M, Mylotte D, Nkomo VT, Pieter Kappetein A. Annual number of candidates for transcatheter aortic valve implantation per country: current estimates and future projections. Eur Heart J 2018;39:2635–2642. 29546396 10.1093/eurheartj/ehy107

[4] Généreux P, Banovic M, Kang DH, Giustino G, Prendergast B, Lindman B, Newby D, Pibarot P, Redfors B, Craig N, Bartunek J, Schwartz A, Seyedin R, Cohen D, Iung B, Leon M, Dweck M. Aortic valve replacement vs clinical surveillance in asymptomatic severe aortic stenosis: a systematic review and meta-analysis. J Am Coll Cardiol 2025;85:912–922. 39641732 10.1016/j.jacc.2024.11.006

[5] Jacquemyn X, Sá MP, Marín-Cuartas M, Bax JJ, Borger MA, Clavel MA, Pibarot P, Généreux P, Sultan I. Early aortic valve replacement versus conservative management in asymptomatic severe aortic stenosis: meta-analysis of time-to-event data of randomized controlled trials. Int J Cardiol 2025;432:133269. 40222660 10.1016/j.ijcard.2025.133269

[6] Praz F, Borger MA, Lanz J, Marin-Cuartas M, Abreu A, Adamo M, Ajmone Marsan N, Barili F, Bonaros N, Cosyns B, De Paulis R, Gamra H, Jahangiri M, Jeppsson A, Klautz RJM, Mores B, Pérez-David E, Pöss J, Prendergast BD, Rocca B, Rossello X, Suzuki M, Thiele H, Tribouilloy CM, Wojakowski W. 2025 ESC/EACTS guidelines for the management of valvular heart disease. Eur Heart J 2025;46:4635–4736. 40878295 10.1093/eurheartj/ehaf194

[7] Généreux P, Schwartz A, Oldemeyer B, Cohen DJ, Redfors B, Prince H, Zhao Y, Lindman BR, Pibarot P, Leon MB. Design and rationale of the evaluation of transcatheter aortic valve replacement compared to surveillance for patients with asymptomatic severe aortic stenosis: the EARLY TAVR trial. Am Heart J 2024;268:94–103. 38056546 10.1016/j.ahj.2023.11.019

[8] Généreux P, Schwartz A, Oldemeyer JB, Pibarot P, Cohen DJ, Blanke P, Lindman BR, Babaliaros V, Fearon WF, Daniels DV, Chhatriwalla AK, Kavinsky C, Gada H, Shah P, Szerlip M, Dahle T, Goel K, O'Neill W, Sheth T, Davidson CJ, Makkar RR, Prince H, Zhao Y, Hahn RT, Leipsic J, Redfors B, Pocock SJ, Mack M, Leon MB; Investigators ETT. Transcatheter aortic-valve replacement for asymptomatic severe aortic stenosis. N Engl J Med 2025;16:217–227. 10.1056/NEJMoa2405880 39466903

[9] Wyss CA, Corti R, Nestelberger T, Candolfi P, Delbaere A, Fischer B, Schwenkglenks M, Telser H. Transcatheter aortic valve implantation with SAPIEN 3 versus surgical aortic valve replacement in patients with symptomatic severe aortic stenosis at low risk of surgical mortality: a cost-utility analysis for Switzerland. Swiss Med Wkly 2024;154:3558. 39509083 10.57187/s.3558

[10] Dubois C, Adriaenssens T, Annemans L, Bosmans J, Callebaut B, Candolfi P, Cornelis K, Delbaere A, Green M, Kefer J, Lancellotti P, Rosseel M, Shore J, Van Der Heyden J, Vermeersch S, Wyffels E. Transcatheter aortic valve implantation versus surgical aortic valve replacement in severe aortic stenosis patients at low surgical mortality risk: a cost-effectiveness analysis in Belgium. Acta Cardiol 2024;79:46–57. 38450496 10.1080/00015385.2023.2282283

[11] Eerdekens R, Kats S, Grutters JP, Green M, Shore J, Candolfi P, Oortwijn W, Harst PV, Tonino P. Cost-utility analysis of TAVI compared with surgery in patients with severe aortic stenosis at low risk of surgical mortality in the Netherlands. Cost Eff Resour Alloc 2024;22:24. 38528520 10.1186/s12962-024-00531-6 PMC10964658

[12] Gilard M, Eltchaninoff H, Iung B, Lefevre T, Spaulding C, Dumonteil N, Mutuon P, Roussel C, Candolfi P, de Pouvourville G, Green M, Shore J. Cost-effectiveness analysis of SAPIEN 3 transcatheter aortic valve implantation procedure compared with surgery in patients with severe aortic stenosis at low risk of surgical mortality in France. Value Health 2022;25:605–613. 35365304 10.1016/j.jval.2021.10.003

[13] Kuck KH, Leidl R, Frankenstein L, Wahlers T, Sarmah A, Candolfi P, Shore J, Green M. Cost-effectiveness of SAPIEN 3 transcatheter aortic valve implantation versus surgical aortic valve replacement in German severe aortic stenosis patients at low surgical mortality risk. Adv Ther 2023;40:1031–1046. 36622552 10.1007/s12325-022-02392-y PMC9988804

[14] Mennini FS, Meucci F, Pesarini G, Vandoni P, Lettino M, Sarmah A, Shore J, Green M, Giardina S. Cost-effectiveness of transcatheter aortic valve implantation versus surgical aortic valve replacement in low surgical risk aortic stenosis patients. Int J Cardiol 2022;357:26–32. 35306028 10.1016/j.ijcard.2022.03.034

[15] Nilsson K, James S, Angeras O, Backes J, Bjursten H, Candolfi P, Gotberg M, Hagstrom H, Malmberg C, Nielsen NE, Sarmah A, Settergren M, Bromilow T. Cost-effectiveness analysis of transcatheter aortic valve implantation versus surgical aortic valve replacement in patients with severe aortic stenosis at low risk of surgical mortality in Sweden. Ups J Med Sci 2025;129:130. 10.48101/ujms.v130.10741 PMC12045074 40313688

[16] Vázquez Rodríguez J, Pinar Bermúdez E, Zamorano JL, Moreu Burgos J, Díaz-Fernández J, García del Blanco B, Sarmah A, Candolfi P, Shore J, Green M. Cost-effectiveness of SAPIEN 3 transcatheter aortic valve implantation in low surgical mortality risk patients in Spain. REC Interv Cardiol 2023;5:38–45.

[17] Curzen N, Candolfi P, MacCarthy P, Lloyd C, Ateasam-Ur-Rahman M, Bromilow T, Sellitto V, Blackman D. Cost-utility analysis of TAVI vs surgery in low-risk patients with severe aortic stenosis in the UK. Appl Health Econ Health Policy 2026;24:365–378. 41203918 10.1007/s40258-025-01012-4 PMC12924842

[18] Généreux P, Lindman BR, Pibarot P. New classification to describe clinical presentation in aortic stenosis: stable, progressive, and acute valve syndrome. Circulation 2025;151:1627–1629. 40489539 10.1161/CIRCULATIONAHA.125.074251

[19] Généreux P, Pellikka P, Lindman B, Pibarot P, Garcia S, Koulogiannis K, Rodriguez E, Thourani V, Dobbles M, Giustino G, Sharma R, Cohen D, Schwartz A, Leon M, Gillam L. Acute valve syndrome in aortic stenosis. Struct Heart 2024;9:e100377. 10.1016/j.shj.2024.100377 PMC12047511 40321310

[20] Automeris.io . WebpPlotDigitizer v5. https://automeris.io/ (August, 2025).

[21] Nombela-Franco L, del Trigo M, Morrison-Polo G, Veiga G, Jimenez-Quevedo P, Abdul-Jawad Altisent O, Campelo-Parada F, Biagioni C, Puri R, DeLarochelliere R, Dumont E, Doyle D, Paradis JM, Quiros A, Almeria C, Gonzalo N, Nunez-Gil I, Salinas P, Mohammadi S, Escaned J, Fernandez-Ortiz A, Macaya C, Rodes-Cabau J. Incidence, causes, and predictors of early </=30 days) and late unplanned hospital readmissions after transcatheter aortic valve replacement. JACC Cardiovasc Interv 2015;8:1748–1757. 26476610 10.1016/j.jcin.2015.07.022

[22] Mack MJ, Leon MB, Thourani VH, Makkar R, Kodali SK, Russo M, Kapadia SR, Malaisrie SC, Cohen DJ, Pibarot P, Leipsic J, Hahn RT, Blanke P, Williams MR, McCabe JM, Brown DL, Babaliaros V, Goldman S, Szeto WY, Genereux P, Pershad A, Pocock SJ, Alu MC, Webb JG, Smith CR; Partner 3 Investigators. Transcatheter aortic-valve replacement with a balloon-expandable valve in low-risk patients. N Engl J Med 2019;380:1695–1705. 30883058 10.1056/NEJMoa1814052

[23] Edwards Lifesciences . Abstract: propensity-matched 8-year outcomes following SAVR with novel calcification-resistant versus contemporary tissue bioprostheses. In: Heart Valve Society. Edwards Lifesciences; Cairo, Egypt, 2025.

[24] Bourguignon T, Bouquiaux-Stablo AL, Candolfi P, Mirza A, Loardi C, May MA, El-Khoury R, Marchand M, Aupart M. Very long-term outcomes of the Carpentier-Edwards Perimount valve in aortic position. Ann Thorac Surg 2015;99:831–837. 25583467 10.1016/j.athoracsur.2014.09.030

[25] Baron SJ, Ryan MP, Chikermane SG, Thompson C, Clancy S, Gunnarsson CL. Long-term risk of reintervention after transcatheter aortic valve replacement. Am Heart J 2024;267:44–51. 37871783 10.1016/j.ahj.2023.10.002

[26] Office for National Statistics . Data from: National Life Tables, England and Wales, period expectation of life, based on data for the years 2017-19. 2025.

[27] Human Mortality Database . National life table Spain. Spain Total population. https://www.mortality.org/Country/Country?cntr=ESP (August, 2025).

[28] Federal Statistical Office of Germany (Statistisches Bundesamt) . National life table Germany (2017/2019). https://www-genesis.destatis.de/genesis/online?operation=abruftabelleBearbeiten&levelindex=1&levelid=1612797015906&auswahloperation=abruftabelleAuspraegungAuswaehlen&auswahlverzeichnis=ordnungsstruktur&auswahlziel=werteabruf&code=12621-0001&auswahltext=&werteabruf=Value+retrieval#abreadcrumb (August, 2025).

[29] The National Institute of Statistics and Economic Studies . Mortalité en 2018 - Tableaux de séries longues. https://www.insee.fr/fr/statistiques/4503155?sommaire=4503178&q=Tables+de+mortalit%C3%A9%20par+sexe%2C%20%C3%A2ge (August, 2025).

[30] Statistics Sweden . Ettårig livslängdstabell för hela riket efter kön och ålder. År 1960 - 2024. https://www.statistikdatabasen.scb.se/pxweb/sv/ssd/START__BE__BE0101__BE0101I/LivslangdEttariga/ (August, 2025).

[31] Belgium StatBel . Sterftetafels en levensverwachting [Online]. https://statbel.fgov.be/sites/default/files/files/documents/bevolking/5.4%20Sterfte%2C%20levensverwachting%20en%20doodsoorzaken/5.4.3%20Sterftetafels%20en%20levensverwachting/sterftetafelsAE.xls (August, 2025).

[32] Italian National Institute of Statistics . National life tables Italy (2018). http://dati.istat.it/Index.aspx?DataSetCode=DCIS_MORTALITA1&Lang=en (August, 2025).

[33] Statline Netherlands . Levensverwachting; geslacht, leeftijd (per jaar en periode van vijf jaren). https://opendata.cbs.nl/statline/#/CBS/nl/dataset/37360ned/table?from (August, 2025).

[34] Hernández Alava M, Pudney SWA. Estimating EQ-5D by Age and Sex for the UK. NICE DSU report. 2022. https://www.sheffield.ac.uk/nice-dsu/methods-development/estimating-eq-5d

[35] Burstrom K, Johannesson M, Diderichsen F. A comparison of individual and social time trade-off values for health states in the general population. Health Policy 2006;76:359–370. 16214258 10.1016/j.healthpol.2005.06.011

[36] Szende A, Janssen B, Cabases J. Self-reported population health: An international perspective based on EQ-5D. Springer; 2014. 29787044

[37] Luengo-Fernandez R, Gray AM, Bull L, Welch S, Cuthbertson F, Rothwell PM; Oxford Vascular Study. Quality of life after TIA and stroke: ten-year results of the Oxford vascular study. Neurology 2013;81:1588–1595. 24107865 10.1212/WNL.0b013e3182a9f45f PMC3806919

[38] Généreux P, Redfors B, Pibarot P, Lindman BR, Giustino G, Dratch A, Murphy S, Chikermane S, Leon MB, Baron SJ. Health care cost and resource utilization after aortic valve replacement according to the extent of cardiac damage. Circ Cardiovasc Interv 2025;18:e014945. 40190262 10.1161/CIRCINTERVENTIONS.124.014945

[39] Généreux P, Giustino G, Lindman BR, Pibarot P, Baron SJ, Asselin C, Dratch A, Murphy SME, Chikermane S, Thourani VH, Koulogiannis KP, Schwartz A, Leon MB, Pellikka PP, Gillam LD. Acute valve syndrome before aortic valve replacement: impact on clinical outcomes, health care costs, and resource use. J Am Heart Assoc 2025;14:e043486. 41025452 10.1161/JAHA.125.043486 PMC12684531

[40] Kang DH, Park SJ, Rim JH, Yun SC, Kim DH, Song JM, Choo SJ, Park SW, Song JK, Lee JW, Park PW. Early surgery versus conventional treatment in asymptomatic very severe aortic stenosis. Circulation 2010;121:1502–1509. 20308614 10.1161/CIRCULATIONAHA.109.909903

[41] Ademi Z, Rodda SE, Vivoda K, Hennessy S, Fenton O, Ware JS. Highlights from the manifesto on the health economics of cardiovascular disease prevention. Pharmacoeconomics 2025;43:1281–1292. 40921978 10.1007/s40273-025-01537-5 PMC12534329

[42] Oude Wolcherink MJ, Behr CM, Pouwels X, Doggen CJM, Koffijberg H. Health economic research assessing the value of early detection of cardiovascular disease: a systematic review. Pharmacoeconomics 2023;41:1183–1203. 37328633 10.1007/s40273-023-01287-2 PMC10492754

