# Supplementary material for: Cost-effectiveness analysis of transcatheter aortic valve implantation for asymptomatic severe aortic stenosis across nine European countries
Source: Eur Heart J Open. 2026 Jul 23;6(4):oeag115. doi: 10.1093/ehjopen/oeag115 (PMC13394697; doi:10.1093/ehjopen/oeag115)
Supplement: oeag115_Supplementary_Data [file oeag115_supplementary_data.docx]

# Supplementary materials

## Additional information

### Willingness-to-pay thresholds

For several countries included in this analysis, there are no explicit and widely accepted WTPs. As such, commonly used WTPs have been employed based on previous literature. For Belgium, Switzerland, Spain, Italy and Sweden, a €30,000, 50,000 CHF, €30,000, €30,000 and 1,000,000 SEK per QALY threshold has been assumed, respectively, based on use in previous studies (see table S1).^1-7^

### Parametric survival analysis

The cumulative incidence of the CS arm converting to AVR was illustrated by symptom level (namely acute valve syndrome, progressive valve syndrome and asymptomatic) from Généreux (2024).^8^ This Kaplan-Meier (KM) data was digitized and extrapolated. The parametric models fitted to the KM data were exponential, Weibull, Gompertz, log-normal, log-logistic and generalised Gamma. Coefficients were produced for each of these parametric models and used to extrapolate time-to-event curves beyond the five-year trial period.^8^ Akaike information criterion (AIC), Bayesian information criterion (BIC) values, and visual graphical checks were used to determine the statistically best-fitting parametric curve for the digitized dataset, which was the exponential parametric model. Other distributions were tested in scenario analyses.

### General population mortality risk

Health state-specific relative risks were applied to general population mortality age- and sex-adjusted rates for each country in the base case. Life tables for the years 2017-2019 were used to avoid more recent data sets that may be affected by COVID-19.^9-16^ The values reported for each country, apart from the UK, which started at 16 years old, started at the mortality risk for an individual who is 30 years old. The Swiss values reflected the German country-specific data reported due to a lack of Swiss-specific data (see table S2).

### Trial-based mortality approach

The five-year all-cause death over time from the EARLY TAVR trial was digitized using WebPlotDigitizer.^17,18^ Using the same parametric survival method as was used for the conversion to AVR data, the five-year trial-based mortality data was extrapolated to reflect the base case lifetime horizon. The model contained KM data for both model arms from the digitized graph, coefficients, goodness-of-fit statistics and Cholesky matrices.

The Gompertz parametric model had the highest AIC value, followed by Weibull. However, there was a minimal difference between the values (<2) and a visual check on the graph and the observed effect on sensitivity analysis outputs indicated that a Weibull parametric model was a better fit for the survival data. Therefore, the Weibull model was used in this trial-based approach.

Over the five-year EARLY TAVR trial period, 8.4% of the participants assigned to early TAVR and 9.2% of those assigned to CS died.^17^ These data from the trial lead the model population mortality risk to appear better than the general population mortality rates for people of the same age in the UK, despite the cohort having severe aortic stenosis. This lacked face validity when presented graphically. Therefore, the health-state specific mortality approach was used in the base case. An additional mortality approach option was included for scenario analysis, where the trial-based approach is used for the first five years of the time horizon, in line with the EARLY TAVR trial length, and then the health-state specific approach is used for the remainder of the time horizon, as described in the main text. This scenario was done to investigate the impact of the trial-based mortality rates whilst applying the health-state specific approach to the remaining years of the lifetime horizon, which may be more reflective of what is seen in clinical practice.

### General population norm utility values

Health state specific utility adjustment factors were applied multiplicatively to age- and sex-stratified general population norm EQ-5D values for each country.^19-21^ Country-specific and age-categorised time-trade-off (TTO) value sets presenting general population norm utility values for Belgium, Switzerland, Germany, Spain, France and Italy were informed by Szende (2014).^21^ The Swiss TTO values reflected the German country-specific data reported due to a lack of Swiss-specific data. Because the value sets reflected age categories, these data sets were linearly interpolated to gradually increase for each age year. These are shown in Table S4.

### Reintervention base case approach

Outcomes at 1, 2 and 5 years of the PARTNER 3 clinical trial have been used to inform the first 7 years of the model.^22^ Years 6 and 7 were assumed equal to the 3- to 5-year values from PARTNER 3 to ensure that the probabilities increased over time. Bourguignon (2015) informed the values from year 8 onwards.^23^ Longer-term outcomes of the Carpentier-Edwards Perimount valve in aortic position were available from Bourguignon (2015), who reported reintervention rates up to 22 years post-AVR. Reintervention probabilities from a competing risk regression were stratified by age, and probabilities from the Bourguignon (2015) paper that correspond to the ones from an average age of 76 were implemented to be consistent with the EARLY TAVR trial cohort.^17^ Although this study reflects a SAVR procedure, the probabilities were deemed generalisable to TAVR procedures and the S3/S3U valve. Given the lack of clinical data after 22 years, the reintervention rate is assumed to remain constant over the following years of the time horizon.

The monthly probabilities were applied directly into the early TAVR arm calculations to inform how many people undergo reintervention of AVR in each cycle, since it was known that the entire cohort undergo AVR in the first model cycle and therefore how many cycles had passed since AVR to apply the correct time-dependent reintervention probability. The CS arm cohort received AVR over time rather than within the first cycle, in line with the conversion to AVR time-to-event data. Markov models are memory-less, and therefore the number of cycles that passed since undergoing index AVR could not be tracked to ensure the correct monthly probability had been applied. Therefore, an assumption was made to calculate one single probability that was applied in all years. A weighted average approach was taken, with the probability for each year multiplied by the number of people alive in the arm each year over the total across all years. The final probability for the CS arm was 0.12% per month. See Table S5 for the base case inputs.

### Reintervention scenarios

Baron (2024) presented crude rates of reintervention at each year for 10 years following the index TAVR procedure in Figure 1.^24^ These reflect more recent analysis and longer-term data on the rate of reintervention than the Bourguignon (2015) study and so have been included in the model as alternative reintervention rates available for scenario analysis. The 10-year values were extrapolated to reflect the base case lifetime horizon. From years 11 to 20 the rates were linearly interpolated to increase gradually at the same rate as the first 10 years. From year 22 onwards the rate of reintervention is assumed to remain constant to align with the method used to apply the Bourguignon (2015) values.^23^ The weighted average for the CS arm was calculated to be 0.04%.

The rate of receiving reintervention over time has been estimated to be lower in instances of AVR where the SAPIEN 3 Ultra RESILIA valve has been used. Therefore, time to event data reflecting years that individuals have freedom from reintervention following undergoing AVR due to structural valve deterioration was applied in the model as a scenario.^25^

Annual probabilities for each data source are presented in Table S5.

### Real world data scenarios

The CS cohort of the EARLY TAVR trial may not be representative of clinical practice. This is because the median time between presenting with AS symptoms and receiving an AVR procedure was measured as 32 days, and it is likely that this would be longer in clinical practice. This means that those in the CS arm might have experienced symptoms for shorter, been less likely to present with advanced symptoms or had a reduced chance of unplanned hospitalisation, in the trial compared with clinical practice.

A systematic review and meta-analysis paper by Généreux (2024) details the findings of 12 observational studies and 4 RCTs comparing AVR with CS in people with aSAS.^26^ The key findings of this paper include incident rate ratios (IRRs) that reflect the relative difference in rates of all-cause mortality, heart failure-related hospitalisation and stroke experienced when people receive early TAVR compared with CS. These were incorporated in the model in scenario analyses (see Table S6 and S7).

### AVS scenario analysis

In the model, it has been assumed that the CS arm cohort are asymptomatic until they become symptomatic and undergo AVR, in line with the EARLY TAVR trial conversion to AVR data.^17^ In the trial participants in the CS arm were categorised by symptom severity upon undergoing AVR: asymptomatic, progressive valve syndrome (PVS) or acute valve syndrome (AVS).^17^ Généreux (2024) presents the trial-based symptom level breakdown, which indicated a majority of the cohort as having PVS (58.5%). However, in a recent real-world dataset analysis of 17,838 adults with moderate or severe AS undergoing AVR the majority of people had AVS, which is more severe (51.7%).^27^ Therefore, the trial-based proportions by symptom level may not be representative of clinical practice and the risk of event rates may be underestimated. The HFH event rate related hazard ratios associated with people who had PVS or AVS relative to those who were asymptomatic were reported in the real-world data study.^27^

A weighted hazard ratio of 2.4 was applied to the first two years of the model time horizon to increase the rate of HFH in the CS arm for scenario analysis (Table S8a). This scenario adjusted the HFH rates to be more reflective of the AVS breakdown that is seen in clinical practice for the average follow-up period of the real-world study and the symptom-level-specific hazard ratios reflecting the increased risk of HFH events for those with PVS or AVS compared with no symptoms.^27^

The EARLY TAVR trial-based proportions by symptom level of the CS arm may not be representative of clinical practice, meaning the risk of mortality in the base case model may have been underestimated. Based on the symptom level breakdowns from the EARLY TAVR trial and the real-world data study, weighted hazard ratios were calculated. These weighted hazard ratios were applied to the CS overall survival data for scenario analysis (Table S8b).

The proportion of individuals with AVS will likely incur higher costs whilst undergoing AVR than may be estimated in the CS arm of the base case model. A recent study indicates the relative increase in cost of AVR procedure for those with PVS or AVS compared with those who are asymptomatic.^28^ A scenario was conducted in which a weighted relative risk of 1.1, as informed by symptom-level specific relative risks and the EARLY TAVR trial-based breakdown of symptom-level proportions, was applied to the total CS arm AVR procedure cost to address this potential underestimation (Table S8c).

### Summary of scenario analyses

A full list of scenarios is listed below:

- Scenario 1 – 2: Younger starting ages (65 and 70)
- Scenario 3 – 4: Different reintervention rates (Baron and S3UR) (Table S5)
- Scenario 5 – 6: Delayed AVR by 6 or 12 months (cost impact only)
- Scenario 7 – 11: Use of incident rate ratios or hazard ratios from a meta-analysis (Table S6 and S7)
- Scenario 12: All scenarios associated with AVS at once (scenario 13, 14 and 15 and 26)
- Scenario 13: Applied a symptom-level specific risk of HFH event rates for the first two years of the time horizon to the CS arm to make CS cohort of the EARLY TAVR trial more representative of clinical practice (Table S8a)
- Scenario 14: Weighted the CS arm alive and well health state by the symptom presentation breakdown from the EARLY TAVR trial and then applied relative risks or hazard ratios reported from real world data (Table S8b)
- Scenario 15: Weighted the CS arm alive and well health state by the symptom presentation breakdown from real-world evidence and then applied relative risks or hazard ratios reported from real world data (Table S8b)
- Scenario 16: Pre-procedure and 30 days post procedure HRQoL decrements applied to both arms from EARLY TAVR trial [pre-procedure single cycle decrement of 0 applied to the TAVI arm and 0.005 applied to the CS arm] [post-procedure decrement of 0 applied to the TAVI arm and a decrement of 0.001 applied to the CS arm]. Unpublished data.
- Scenario 17: Equal 5+ year event rates (stroke and HFH) across clinical surveillance and TAVI arms
- Scenario 18 – 22: Alternative parametric distributions for conversion to AVR in the CS arm
- Scenario 23: EARLY TAVR trial-based mortality for the entire time horizon (unadjusted).
- Scenario 24: EARLY TAVR trial-based mortality adjusted by general population norms for the entire time horizon.
- Scenario 25: EARLY TAVR trial-based mortality for first 5 years, followed by health state relative risk values onwards
- Scenario 26: Increased procedure costs for people with PVS and AVS at the time of procedure (Table S8c)
- Scenario 27: Explore the impact of using a non-zero value for the decrement associated with PPM implantation. Makino reported a 0.009 decrement (utility loss of 0.073 lasting 1.5 month) for the implantation of a leadless pacemaker in patients with bradycardia and atrial fibrillation in Australia ^29^
- Scenario 28: 5 year time horizon

### Sensitivity analysis

Deterministic sensitivity analysis (DSA) was conducted to account for first-order uncertainty around the data used for the majority of input parameter values (with the exception of inputs such as reintervention rates and general population mortality). DSA involves adjusting the base case value used for individual parameters, within realistic ranges, to observe the impact on the model results. The main output from this sensitivity analysis was a tornado diagram, which summarises the impact that changing each parameter has on the selected model result, typically the net monetary benefit (NMB), and ranks the size of the individual impact from top to bottom. This both identifies the inputs that have the largest impact on the results, and any results that, when varied independently, could change the direction of the cost-effectiveness result. For instance, if an input is varied which causes the NMB to become negative rather than positive. All model inputs that did not have a specified range were varied in the DSA at an assumed range of +/- 20%. It is important to note that any values that changed each year were not included in the DSA. These include the rate of reintervention, the general population norms for utility values and the general population mortality rate. For any two inputs that were required to sum to 100%, only one input was changed with the other set to ‘1 – the new value’. Any three or more inputs that were required to sum to 100% were not included in the DSA.

Probabilistic sensitivity analysis (PSA) was also conducted to account for second-order uncertainty in relation to the model inputs, and quantify the level of confidence in the results of the analysis. This is done by running iterations of the model with all input variables that are subject to uncertainty simultaneously, and were suitable to be included, being varied randomly according to their observed distribution. Distributions are fitted based on the type of parameter being varied. For instance, a beta distribution is applied to proportions and probabilities, a log normal distribution is applied to relative risks and hazard ratios, and a gamma distribution is applied to costs and resource use values. Where statistical parameters were not available from the literature, model inputs were varied in the PSA at an assumed range of +/- 20%. Similarly to the DSA, for any two inputs that were required to sum to 100%, only one input was changed with the other set to ‘1 – the new value’. Any three or more inputs that were required to sum to 100% were not included in the PSA.

The model used a sample of 5,000 iterations, each iteration using a different set of values for the inputs, to ensure stable results. However, the model user was able to vary the number of iterations used to determine results. The convergence of the PSA results was recorded to confirm that 5,000 iterations were sufficient to achieve stability.

Outputs of the PSA include an estimated level of confidence in the direction of the model results. This result reflects the proportion of ICER results that fall below the threshold and, therefore, in what proportion of iterations early TAVR was estimated to be cost-effective. The PSA results are also presented graphically, in the form of a cost-effectiveness plane and a cost-effectiveness acceptability curve (CEAC). The cost-effectiveness plane presents the ICER results from all 5,000 iterations along with the deterministic result and the cost-effectiveness threshold. The CEAC displays the probability that the intervention is cost-effective at various cost-effectiveness thresholds. See Figures S11 to S28 for the CEACs and cost-effectiveness planes.

## Tables

### Inputs

Table S1: Discount rates and willingness-to-pay thresholds by country

|  | Value | DSA low / high value | Source |
| --- | --- | --- | --- |
| **BE** | | | |
| Annual discount rate: costs | 3.0% | 0.0% : 5.0% | Mattias et al (2025)^30^ |
| Annual discount rate: benefits | 1.5% | 0.0% : 5.0% | Mattias et al (2025)^30^ |
| Willingness-to-pay threshold | € 30,000 | Not varied | Dubois et al (2024): hypothetical threshold in line with previous publications ^1^ |
| **CH** | | | |
| Annual discount rate: costs | 3.0% | 1.5% : 6.0% | IQWIG (2022)^31^  Assumed to be the same as Germany. |
| Annual discount rate: benefits | 3.0% | 1.5% : 6.0% |  |
| Willingness-to-pay threshold | 50,000 CHF | Not varied | Wyss et al (2024): hypothetical threshold in line with previous publications^7^ |
| **DE** | | | |
| Annual discount rate: costs | 3.0% | 1.5% : 6.0% | IQWIG (2022)^31^ |
| Annual discount rate: benefits | 3.0% | 1.5% : 6.0% |  |
| Willingness-to-pay threshold | € 35,000 | Not varied | IQWIG (2022)^31^  Assumption. No explicit consented WTP in Germany. |
| **ES** | | | |
| Annual discount rate: costs | 3.0% | 1.5% : 6.0% | Lopez-Bastida et al (2010)^32^ |
| Annual discount rate: benefits | 3.0% | 1.5% : 6.0% |  |
| Willingness-to-pay threshold | € 30,000 | Not varied | Assumption based on previous publication. No explicated consented WTP in Spain. ^33^ |
| **FR** | | | |
| Annual discount rate: costs | 2.5% | 1.5% : 6.0% | HAS (2020)^34^ |
| Annual discount rate: benefits | 2.5% | 1.5% : 6.0% |  |
| Willingness-to-pay threshold | € 50,000 | Not varied |  |
| **IT** | | | |
| Annual discount rate: costs | 3.0% | 1.5% : 6.0% | Farmaco (2020)^35^ |
| Annual discount rate: benefits | 3.0% | 1.5% : 6.0% |  |
| Willingness-to-pay threshold | € 30,000 | Not varied | Menninni et al (2022): hypothetical threshold in line with previous publications^4^ |
| **NL** | | | |
| Annual discount rate: costs | 3.0% | 1.5% : 5.0% | Geuzinge et al (2025)^36^ |
| Annual discount rate: benefits | 1.5% | 1.0% : 4.0% |  |
| Willingness-to-pay threshold | € 50,000 | Not varied |  |
| **SE** | | | |
| Annual discount rate: costs | 3.0% | 1.5% : 6.0% | TLV (2017)^37^ |
| Annual discount rate: benefits | 3.0% | 1.5% : 6.0% |  |
| Willingness-to-pay threshold | 1,000,000 SEK | Not varied | Assumption following previous model. Nilson et at (2025)^5^ |
| **UK** | | | |
| Annual discount rate: costs | 3.5% | 1.5% : 6.0% | NICE (2022)^38^ |
| Annual discount rate: benefits | 3.5% | 1.5% : 6.0% |  |
| Willingness-to-pay threshold | £20,000 | Not varied |  |

BE: Belgium; CH: Switzerland; DE: Germany; DSA: Deterministic sensitivity analysis; ES: Spain; FR: France; IT: Italy; NL: Netherlands; Probabilistic sensitivity analysis; SE: Sweden; UK: United Kingdom

Table S2: Health state-specific mortality relative risks by country

|  | Value | DSA low / high value | PSA distribution | Source |
| --- | --- | --- | --- | --- |
| **BE** | | | | |
| Disabling stroke (month 1) | 2.30 | +/- 20% | Log normal | Dennis et al ^39^ |
| Non-disabling stroke (month 1) | 1.50 | +/- 20% | Log normal | Prencipe et al ^40^ |
| Disabling stroke (month 2+) | 2.30 | +/- 20% | Log normal | Dennis et al ^39^ |
| Non-disabling stroke (month 2+) | 1.50 | +/- 20% | Log normal | Prencipe et al ^40^ |
| HFH | 1.00 | 1.0 : 1.20 | Log normal | Assumed equal to general population |
| **CH** | | | | |
| Disabling stroke (month 1) | 2.30 | +/- 20% | Log normal | Gandjour et al ^41^ |
| Non-disabling stroke (month 1) | 1.50 | +/- 20% | Log normal | Prencipe et al ^40^ |
| Disabling stroke (month 2+) | 2.30 | +/- 20% | Log normal | Gandjour et al ^41^ |
| Non-disabling stroke (month 2+) | 1.50 | +/- 20% | Log normal | Prencipe et al ^40^ |
| HFH | 1.00 | +/- 20% | Log normal | Assumed equal to general population |
| **DE** | | | | |
| Disabling stroke (month 1) | 2.30 | +/- 20% | Log normal | Gandjour et al ^41^ |
| Non-disabling stroke (month 1) | 1.50 | +/- 20% | Log normal | Prencipe et al ^40^ |
| Disabling stroke (month 2+) | 2.30 | +/- 20% | Log normal | Gandjour et al ^41^ |
| Non-disabling stroke (month 2+) | 1.50 | +/- 20% | Log normal | Prencipe et al ^40^ |
| HFH | 1.00 | +/- 20% | Log normal | Assumed equal to general population |
| **ES** | | | | |
| Disabling stroke (month 1) | 2.05 | +/- 20% | Log normal | de Andres-Nogales et al ^42^ |
| Non-disabling stroke (month 1) | 1.50 | +/- 20% | Log normal | Prencipe et al(^40^ |
| Disabling stroke (month 2+) | 2.05 | +/- 20% | Log normal | de Andres-Nogales et al ^42^ |
| Non-disabling stroke (month 2+) | 1.50 | +/- 20% | Log normal | Prencipe et al ^40^ |
| HFH | 1.00 | +/- 20% | Log normal | Assumed equal to general population |
| **FR** | | | | |
| Disabling stroke (month 1) | 2.30 | +/- 20% | Log normal | Gilard et al ^43^; Shah et al ^44^ |
| Non-disabling stroke (month 1) | 1.50 | +/- 20% | Log normal | Prencipe et al ^40^ |
| Disabling stroke (month 2+) | 2.30 | +/- 20% | Log normal | Gilard et al ^43^, Shah et al ^44^ |
| Non-disabling stroke (month 2+) | 1.50 | +/- 20% | Log normal | Prencipe et al ^40^ |
| HFH | 1.00 | +/- 20% | Log normal | Assumed equal to general population |
| **IT** | | | | |
| Disabling stroke (month 1) | 2.05 | +/- 20% | Log normal | Ruggeri et al ^45^ |
| Non-disabling stroke (month 1) | 1.50 | +/- 20% | Log normal | Prencipe et al ^40^ |
| Disabling stroke (month 2+) | 2.05 | +/- 20% | Log normal | Ruggeri et al ^45^ |
| Non-disabling stroke (month 2+) | 1.50 | +/- 20% | Log normal | Prencipe et al ^40^ |
| HFH | 1.00 | +/- 20% | Log normal | Assumed equal to general population |
| **NL** | | | | |
| Disabling stroke (month 1) | 2.30 | +/- 20% | Log normal | Geisler et al ^46^ |
| Non-disabling stroke (month 1) | 1.50 | +/- 20% | Log normal | Prencipe et al ^40^ |
| Disabling stroke (month 2+) | 2.30 | +/- 20% | Log normal | Geisler et al ^46^ |
| Non-disabling stroke (month 2+) | 1.50 | +/- 20% | Log normal | Prencipe et al ^40^ |
| HFH | 1.00 | +/- 20% | Log normal | Assumed equal to general population |
| **SE** | | | | |
| Disabling stroke (month 1) | 2.30 | +/- 20% | Log normal | Geisler et al ^46^ |
| Non-disabling stroke (month 1) | 1.50 | +/- 20% | Log normal | Prencipe et al ^40^ |
| Disabling stroke (month 2+) | 2.30 | +/- 20% | Log normal | Geisler et al ^46^ |
| Non-disabling stroke (month 2+) | 1.50 | +/- 20% | Log normal | Prencipe et al ^40^ |
| HFH | 1.00 | +/- 20% | Log normal | Assumed equal to general population |
| **UK** | | | | |
| Disabling stroke (month 1) | 5.22 | +/- 20% | Log normal | Myat et al ^47^ |
| Non-disabling stroke (month 1) | 5.22 | +/- 20% | Log normal | Myat et al ^47^ |
| Disabling stroke (month 2+) | 1.58 | +/- 20% | Log normal | Myat et al ^47^  NICE NG208 ^48^ |
| Non-disabling stroke (month 2+) | 1.58 | +/- 20% | Log normal |  |
| HFH | 1.00 | +/- 20% | Log normal | Assumed equal to general population |

BE: Belgium; CH: Switzerland; DE: Germany; DSA: Deterministic sensitivity analysis; ES: Spain; FR: France; IT: Italy; NL: Netherlands; Probabilistic sensitivity analysis; SE: Sweden; UK: United Kingdom

Table S3: Cost inputs by country (2022/2023 cost year)

|  | Value (cost year 2022/2023) | DSA low / high value | PSA distribution | Source |
| --- | --- | --- | --- | --- |
| **BE** | | | | |
| Echocardiogram (unit cost) | € 65 | +/- 20% | Gamma | KCE et al (2011)^49^ |
| Consultation (unit cost) | € 35 | +/- 20% | Gamma |  |
| Disabling stroke (initial cost) | € 14,892 | +/- 20% | Gamma | Dubois et al (2024)^1^ |
| Disabling stroke (ongoing cost per year) | € 4,246 | +/- 20% | Gamma |  |
| Non-disabling stroke (initial cost) | € 7,239 | +/- 20% | Gamma |  |
| Non-disabling stroke (ongoing cost per year) | € 0 | +/- 20% | Gamma | Assumption |
| HFH (unit cost per day) | € 8,348 | +/- 20% | Gamma | Dubois et al (2024)^1^ |
| PPM (initial cost) | € 3,579 | +/- 20% | Gamma |  |
| PPM (ongoing cost per month) | € 136 | +/- 20% | Gamma |  |
| TAVR: Cost of intervention | € 8,124 | +/- 20% | Gamma | Unpublished^†^ |
| TAVR: Cost of valve | € 14,329 | +/- 20% | Gamma | Informed by Edwards |
| SAVR: Cost of intervention (complex) | € 24,146 | +/- 20% | Gamma | Dubois et al (2024)^1^ |
| **CH** | | | | |
| Echocardiogram (unit cost) | 307 CHF | +/- 20% | Gamma | Local expert |
| Consultation (unit cost) | 106 CHF | +/- 20% | Gamma |  |
| Disabling stroke (initial cost) | 20,662 CHF | +/- 20% | Gamma | Wyss et al (2024)^7^ |
| Disabling stroke (ongoing cost per year) | 43,776 CHF | +/- 20% | Gamma |  |
| Non-disabling stroke (initial cost) | 7,381 CHF | +/- 20% | Gamma |  |
| Non-disabling stroke (ongoing cost per year) | 0 CHF | +/- 20% | Gamma | Assumption |
| HFH (unit cost per day) | 9,259 CHF | +/- 20% | Gamma | Wyss et al (2024)^7^ |
| PPM (initial cost) | 13,176 CHF | +/- 20% | Gamma |  |
| PPM (ongoing cost per month) | 329 CHF | +/- 20% | Gamma |  |
| TAVR: Cost of intervention (inc. valve) | 50,145 CHF | +/- 20% | Gamma |  |
| SAVR: Cost of intervention (complex) | 45,521 CHF | +/- 20% | Gamma |  |
| **DE** | | | | |
| Echocardiogram (unit cost) | € 59 | +/- 20% | Gamma | Schuler et al (2010)^50^ |
| Consultation (unit cost) | € 218 | +/- 20% | Gamma | Wickle et al (2021)^51^ |
| Disabling stroke (initial cost) | € 9,441 | +/- 20% | Gamma | Kuck et al (2023)^52^ |
| Disabling stroke (ongoing cost per year) | € 13,799 | +/- 20% | Gamma |  |
| Non-disabling stroke (initial cost) | € 7,888 | +/- 20% | Gamma |  |
| Non-disabling stroke (ongoing cost per year) | € 0 | +/- 20% | Gamma | Assumption |
| HFH (unit cost per day) | € 6,185 | +/- 20% | Gamma | Kuck et al (2023)^52^ |
| PPM (initial cost) | € 1,693 | +/- 20% | Gamma |  |
| PPM (ongoing cost per month) | € 131 | +/- 20% | Gamma |  |
| TAVR: Cost of intervention (inc. valve) | € 27,031 | +/- 20% | Gamma |  |
| SAVR: Cost of intervention (complex) | € 25,957 | +/- 20% | Gamma |  |
| **ES** | | | | |
| Echocardiogram (unit cost) | € 348 | +/- 20% | Gamma | Informed by current clinical practice |
| Consultation (unit cost) | € 190 | +/- 20% | Gamma | Informed by current clinical practice |
| Disabling stroke (initial cost) | € 7,324 | +/- 20% | Gamma | Vázquez Rodríguez et al (2023)^53^ |
| Disabling stroke (ongoing cost per year) | € 30,020 | +/- 20% | Gamma |  |
| Non-disabling stroke (initial cost) | € 6,880 | +/- 20% | Gamma |  |
| Non-disabling stroke (ongoing cost per year) | € 0 | +/- 20% | Gamma | Assumption |
| HFH (unit cost per day) | € 3,353 | +/- 20% | Gamma | Vázquez Rodríguez et al (2023)^53^ |
| PPM (initial cost) | € 8,499 | +/- 20% | Gamma |  |
| PPM (ongoing cost per month) | € 62 | +/- 20% | Gamma |  |
| TAVR: Cost of intervention (inc. valve) | € 26,860 | +/- 20% | Gamma |  |
| SAVR: Cost of intervention (complex) | € 14,935 | +/- 20% | Gamma |  |
| **FR** | | | | |
| Echocardiogram (unit cost) | € 97 | +/- 20% | Gamma | Maladie et al (2019)^54^ |
| Consultation (unit cost) | € 39 | +/- 20% | Gamma |  |
| Disabling stroke (initial cost) | € 6,784 | +/- 20% | Gamma | Gilard et al (2022)^43^ |
| Disabling stroke (ongoing cost per year) | € 15,150 | +/- 20% | Gamma |  |
| Non-disabling stroke (initial cost) | € 6,784 | +/- 20% | Gamma |  |
| Non-disabling stroke (ongoing cost per year) | € 0 | +/- 20% | Gamma | Assumption |
| HFH (unit cost per day) | € 2,230 | +/- 20% | Gamma | Gilard et al (2022)^43^ |
| PPM (initial cost) | € 8,898 | +/- 20% | Gamma |  |
| PPM (ongoing cost per month) | € 144 | +/- 20% | Gamma |  |
| TAVR: Cost of intervention | € 8,152 | +/- 20% | Gamma |  |
| TAVR: Cost of valve | € 14,559 | +/- 20% | Gamma |  |
| SAVR: Cost of intervention (complex) | € 22,638 | +/- 20% | Gamma |  |
| **IT** | | | | |
| Echocardiogram (unit cost) | € 66 | +/- 20% | Gamma | Corrao et al (2024)^55^ |
| Consultation (unit cost) | € 26 | +/- 20% | Gamma | Marini et al (2023)^56^ |
| Disabling stroke (initial cost) | € 11,614 | +/- 20% | Gamma | Mennini et al (2022)^4^ |
| Disabling stroke (ongoing cost per year) | € 4,433 | +/- 20% | Gamma |  |
| Non-disabling stroke (initial cost) | € 1,131 | +/- 20% | Gamma | Lorenzoni et al (2021)^57^ |
| Non-disabling stroke (ongoing cost per year) | € 0 | +/- 20% | Gamma | Assumption |
| HFH (unit cost per day) | € 6,876 | +/- 20% | Gamma | Mennini et al (2022)^4^ |
| PPM (initial cost) | € 4,756 | +/- 20% | Gamma |  |
| PPM (ongoing cost per month) | € 63 | +/- 20% | Gamma |  |
| TAVR: Cost of intervention (inc. valve) | € 35,005 | +/- 20% | Gamma |  |
| SAVR: Cost of intervention (complex) | € 30,200 | +/- 20% | Gamma |  |
| **NL** | | | | |
| Echocardiogram (unit cost) | € 269 | +/- 20% | Gamma | Luijten et al (2025)^58^ |
| Consultation (unit cost) | € 105 | +/- 20% | Gamma | Cardiovisit DBC code: 099899091 €210 for maximum 2-hour visit ^59^ |
| Disabling stroke (initial cost) | € 18,104 | +/- 20% | Gamma | Eerdekens et al (2024)^60^ |
| Disabling stroke (ongoing cost per year) | € 9,621 | +/- 20% | Gamma |  |
| Non-disabling stroke (initial cost) | € 3,731 | +/- 20% | Gamma |  |
| Non-disabling stroke (ongoing cost per year) | € 0 | +/- 20% | Gamma | Assumption |
| HFH (unit cost per day) | € 2,982 | +/- 20% | Gamma | Eerdekens et al (2024)^60^ |
| PPM (initial cost) | € 14,342 | +/- 20% | Gamma |  |
| PPM (ongoing cost per month) | € 42 | +/- 20% | Gamma |  |
| TAVR: Cost of intervention (inc. valve) | € 40,537 | +/- 20% | Gamma |  |
| SAVR: Cost of intervention (complex) | € 32,004 | +/- 20% | Gamma |  |
| **SE** | | | | |
| Echocardiogram and consultation (unit cost) | 9,352 SEK | +/- 20% | Gamma | Sveriges et al (2024)^61^ |
| Disabling stroke (initial cost) | 106,165 SEK | +/- 20% | Gamma | Nilsson et al (2025)^5^ |
| Disabling stroke (ongoing cost per year) | 51,408 SEK | +/- 20% | Gamma |  |
| Non-disabling stroke (initial cost) | 106,165 SEK | +/- 20% | Gamma |  |
| Non-disabling stroke (ongoing cost per year) | 0 SEK | +/- 20% | Gamma | Assumption |
| HFH (unit cost per day) | 61,340 SEK | +/- 20% | Gamma | Nilsson et al (2025)^5^ |
| PPM (initial cost) | 77,451 SEK | +/- 20% | Gamma |  |
| PPM (ongoing cost per month) | 3,206 SEK | +/- 20% | Gamma |  |
| TAVR: Cost of intervention (inc. valve) | 302,329 SEK | +/- 20% | Gamma |  |
| SAVR: Cost of intervention (complex) | 244,750 SEK | +/- 20% | Gamma |  |
| **UK** | | | | |
| Echocardiogram (unit cost) | £116.41 | +/- 20% | Gamma | NCC (2024): total HRG, RD51A^62^ |
| Consultation (unit cost) | £173.36 | +/- 20% | Gamma | NCC (2024): consultant-led, code 320, WF01A^62^ |
| Disabling stroke (initial cost) | £21,775.42 | +/- 20% | Gamma | NICE NG207: table 28, cost of stroke and post stroke^63^ |
| Disabling stroke (ongoing cost per year) | £7,730.80 | +/- 20% | Gamma |  |
| Non-disabling stroke (initial cost) | £2,370.57 | +/- 20% | Gamma | NCC (2024): weighted average, total HRGs, stroke with CC<10, AA35D-F ^62^ |
| Non-disabling stroke (ongoing cost per year) | £0.00 | +/- 20% | Gamma | Assumption |
| HFH (unit cost per day) | £1,032.12 | +/- 20% | Gamma | NCC (2024): critical care, ward stay, CCU06,XC07Z ^62^ |
| PPM (initial cost) | £3,754.95 | +/- 20% | Gamma | NCC (2024): weighted average, total HRG, EY08A-E ^62^ |
| PPM (ongoing cost per month) | £144.78 | +/- 20% | Gamma | Shore et al (2020): Table 4, average ICD/CRT device replacement cost per month^64^ |
| TAVR: Cost of intervention | £7,512.88 | +/- 20% | Gamma | NHS (2017): table 6.6.1, bed based^65^ |
| TAVR: Cost of valve | £22,500 | +/- 20% | Gamma | Informed by Edwards |
| SAVR: Cost of intervention (complex) | £20,156.78 | +/- 20% | Gamma | NHS (2017): table 6.6.1, home based^65^ |

BE: Belgium; CH: Switzerland; DE: Germany; DSA: Deterministic sensitivity analysis; ES: Spain; FR: France; IT: Italy; NCC, National Cost Collection; NL: Netherlands; PSA: Probabilistic sensitivity analysis; SE: Sweden; UK: United Kingdom.

Costs have been inflated to the 2022/2023 cost year using country specific indices where relevant.

† Unpublished conference presentation HTAi - Innovation through HTA, Virtual annual HTAi meeting, 2021.

Table S4: Health state-specific utility inputs by country

|  | Utility value | DSA low / high value | PSA distribution | Source |
| --- | --- | --- | --- | --- |
| **BE** | | | | |
| Alive and well | 0.76 | +/- 20% | Beta | Szende et al (2014)^21^ Age and gender adjusted |
| Non-disabling stroke | 0.74 | +/- 20% | Beta | Dewilde et al (2019)^66^ |
| Disabling stroke (month 1) | 0.43 | +/- 20% | Beta |  |
| Disabling stroke (month 2+) | 0.51 | +/- 20% | Beta |  |
| Heart failure-related hospitalisation | 0.76 | +/- 20% | Beta | Assumed to be the same as ‘alive and well’ |
| **CH** | | | | |
| Alive and well | 0.84 | +/- 20% | Beta | Szende et al (2014)^21^ Age and gender adjusted |
| Non-disabling stroke | 0.76 | +/- 20% | Beta | Ali et al (2016), Lanitis et al (2014) and Dewilde et al (2019): weighted average^66-68^ |
| Disabling stroke (month 1) | 0.46 | +/- 20% | Beta |  |
| Disabling stroke (month 2+) | 0.64 | +/- 20% | Beta |  |
| Heart failure-related hospitalisation | 0.84 | +/- 20% | Beta | Assumed to be the same as ‘alive and well’ |
| **DE** | | | | |
| Alive and well | 0.84 | +/- 20% | Beta | Szende et al (2014)^21^ Age and gender adjusted |
| Non-disabling stroke | 0.83 | +/- 20% | Beta | Ali et al (2017)^67^ |
| Disabling stroke (month 1) | 0.44 | +/- 20% | Beta |  |
| Disabling stroke (month 2+) | 0.68 | +/- 20% | Beta |  |
| Heart failure-related hospitalisation | 0.84 | +/- 20% | Beta | Assumed to be the same as ‘alive and well’ |
| **ES** | | | | |
| Alive and well | 0.78 | +/- 20% | Beta | Szende et al (2014)^21^ Age and gender adjusted |
| Non-disabling stroke | 0.72 | +/- 20% | Beta | Ali et al (2017)^67^ |
| Disabling stroke (month 1) | 0.16 | +/- 20% | Beta |  |
| Disabling stroke (month 2+) | 0.51 | +/- 20% | Beta |  |
| Heart failure-related hospitalisation | 0.78 | +/- 20% | Beta | Assumed to be the same as ‘alive and well’ |
| **FR** | | | | |
| Alive and well | 0.74 | +/- 20% | Beta | Szende et al (2014)^21^ Age and gender adusted |
| Non-disabling stroke | 0.62 | +/- 20% | Beta | Lanitis et al (2014)^68^ |
| Disabling stroke (month 1) | 0.51 | +/- 20% | Beta |  |
| Disabling stroke (month 2+) | 0.56 | +/- 20% | Beta |  |
| Heart failure-related hospitalisation | 0.74 | +/- 20% | Beta | Assumed to be the same as ‘alive and well’ |
| **IT** | | | | |
| Alive and well | 0.84 | +/- 20% | Beta | Szende et al (2014)^21^ Age and gender adjusted |
| Non-disabling stroke | 0.62 | +/- 20% | Beta | Pradelli et al (2014)^69^ |
| Disabling stroke (month 1) | 0.51 | +/- 20% | Beta |  |
| Disabling stroke (month 2+) | 0.56 | +/- 20% | Beta |  |
| Heart failure-related hospitalisation | 0.84 | +/- 20% | Beta | Assumed to be the same as ‘alive and well’ |
| **NL** | | | | |
| Alive and well | 0.85 | +/- 20% | Beta | Szende et al (2014)^21^ Age and gender adjusted |
| Non-disabling stroke | 0.82 | +/- 20% | Beta | Beaten et al (2010)^70^ |
| Disabling stroke (month 1) | 0.17 | +/- 20% | Beta |  |
| Disabling stroke (month 2+) | 0.63 | +/- 20% | Beta |  |
| Heart failure-related hospitalisation | 0.85 | +/- 20% | Beta | Assumed to be the same as ‘alive and well’ |
| **SE** | | | | |
| Alive and well | 0.79 | +/- 20% | Beta | Burströmet et al (2006)^20^ Age and gender adjusted |
| Non-disabling stroke | 0.67 | +/- 20% | Beta | Lindgren et al (2008)^71^ |
| Disabling stroke (month 1) | 0.44 | +/- 20% | Beta | Lanitis et al (2014)^68^ |
| Disabling stroke (month 2+) | 0.67 | +/- 20% | Beta | Qureshi et al (2011)^72^ |
| Heart failure-related hospitalisation | 0.79 | +/- 20% | Beta | Assumed to be the same as ‘alive and well’ |
| **UK** | | | | |
| Alive and well | 0.78 | +/- 20% | Beta | Hernández Alavaet et al (2022)^19^ Age and gender adjusted |
| Non-disabling stroke | 0.73 | +/- 20% | Beta | Luengo-Fernandez et al (2013)^73^ |
| Disabling stroke (month 1) | 0.13 | +/- 20% | Beta |  |
| Disabling stroke (month 2+) | 0.45 | +/- 20% | Beta |  |
| Heart failure-related hospitalisation | 0.78 | +/- 20% | Beta | Assumed to be the same as ‘alive and well’ |

BE: Belgium; CH: Switzerland; DE: Germany; ES: Spain; FR: France; IT: Italy; NL: Netherlands; SE: Sweden; UK: United Kingdom

Table S5: Base case and scenario data for the reintervention of AVR

|  | Bourguignon (2015) base case annual probability ^22,23^ | Baron (2024) scenario annual rate ^24^ | SAPIEN 3 Ultra RESILIA scenario annual rate ^25^ |
| --- | --- | --- | --- |
| Year 1 | 0.48% | 0.36% | 0.0% |
| Year 2 | 0.44% | 0.17% | 0.0% |
| Year 3 | 0.59% | 0.13% | 0.0% |
| Year 4 | 0.59% | 0.19% | 0.0% |
| Year 5 | 0.59% | 0.26% | 0.3% |
| Year 6 | 0.59% | 0.41% | 0.8% |
| Year 7 | 0.59% | 0.60% | 0.8% |
| Year 8 | 0.61% | 0.51% | 0.8% |
| Year 9 | 1.23% | 0.75% | 0.8% |
| Year 10 | 1.48% | 0.59% | 0.8% |
| Year 11 | 1.92% | 0.62% | 0.8% |
| Year 12 | 2.19% | 0.64% | 0.8% |
| Year 13 | 2.56% | 0.67% | 0.8% |
| Year 14 | 3.14% | 0.69% | 0.8% |
| Year 15 | 3.67% | 0.72% | 0.8% |
| Year 16 | 4.41% | 0.74% | 0.8% |
| Year 17 | 4.99% | 0.77% | 0.8% |
| Year 18 | 5.85% | 0.79% | 0.8% |
| Year 19 | 6.11% | 0.82% | 0.8% |
| Year 20 | 6.42% | 0.85% | 0.8% |
| Year 21 | 6.95% | 0.85% | 0.8% |
| Year 22 | 7.24% | 0.85% | 0.8% |
| Year 23 | 7.24% | 0.85% | 0.8% |
| Year 24 | 7.24% | 0.85% | 0.8% |
| Year 25 | 7.24% | 0.85% | 0.8% |
| Year 26 | 7.24% | 0.85% | 0.8% |
| Year 27 | 7.24% | 0.85% | 0.8% |
| Year 28 | 7.24% | 0.85% | 0.8% |
| Year 29 | 7.24% | 0.85% | 0.8% |
| Year 30 | 7.24% | 0.85% | 0.8% |
| Year 31 | 7.24% | 0.85% | 0.8% |
| Year 32 | 7.24% | 0.85% | 0.8% |
| Year 33 | 7.24% | 0.85% | 0.8% |
| Year 34 | 7.24% | 0.85% | 0.8% |
| Year 35 | 7.24% | 0.85% | 0.8% |
| Year 36 | 7.24% | 0.85% | 0.8% |
| Year 37 | 7.24% | 0.85% | 0.8% |
| Year 38 | 7.24% | 0.85% | 0.8% |
| Year 39 | 7.24% | 0.85% | 0.8% |
| Year 40 | 7.24% | 0.85% | 0.8% |
| Year 41 | 7.24% | 0.85% | 0.8% |
| Year 42 | 7.24% | 0.85% | 0.8% |
| Year 43 | 7.24% | 0.85% | 0.8% |
| Year 44 | 7.24% | 0.85% | 0.8% |
| Year 45 | 7.24% | 0.85% | 0.8% |
| Year 46 | 7.24% | 0.85% | 0.8% |
| Year 47 | 7.24% | 0.85% | 0.8% |
| Year 48 | 7.24% | 0.85% | 0.8% |
| Year 49 | 7.24% | 0.85% | 0.8% |
| Year 50 | 7.24% | 0.85% | 0.8% |

AVR, aortic valve replacement

Table S6: Meta-analysis incident rate ratios ^26^

|  | RCT | Observational | Pooled |
| --- | --- | --- | --- |
| All-cause mortality | 0.66 | 0.36 | 0.42 |
| HFH | 0.30 | 0.29 | 0.27 |
| Stroke | 0.63 | 1.30 | 0.82 |

HFH, Heart failure-related hospitalisation; RCT, randomised controlled trial

Table S7: Meta-analysis hazard ratios

|  | Early TAVR RCT | Meta-analysis RCT |
| --- | --- | --- |
| All-cause mortality | 0.93 | 0.68 |
| HFH | 0.32 | 0.28 |
| Stroke | 0.62 | 0.62 |

HFH, Heart failure-related hospitalisation; RCT, randomised controlled trial

Table S8a: AVS risk of increased HFH event rate for scenario analysis

| Symptom level | Real-world data (2024) ^27^ | HFH HRs ^27^ |
| --- | --- | --- |
| No symptoms | 14.0% | 1.0 |
| Progressive valve syndrome | 34.3% | 1.5 |
| Acute valve syndrome | 51.7% | 3.3 |
| **Weighted risk increase** | | **2.4** |

AVS, aortic valve stenosis; HFH, heart failure-related hospitalisation; HR, hazard ratio

Table S8b: AVS risk of increased mortality for scenario analysis

| Symptom level | Hazard ratios ^27^ | Trial-based ^17^ | Real-world data (2024) ^27^ |
| --- | --- | --- | --- |
| No symptoms | 1.0 | 2.3% | 14.0% |
| Progressive valve syndrome | 1.1 | 58.5% | 34.3% |
| Acute valve syndrome | 2.2 | 39.2% | 51.7% |
| **Weighted mortality hazard ratio for the CS arm** | | **1.5** | **1.7** |

AVS, aortic valve stenosis; CS, clinical surveillance

Table S8c: AVS risk of increased AVR cost for scenario analysis

| Symptom level | Trial-based breakdown ^17^ | AVR RRs ^28^ |
| --- | --- | --- |
| No symptoms | 2.3% | 1.0 |
| Progressive valve syndrome | 58.5% | 1.1 |
| Acute valve syndrome | 39.2% | 1.2 |
| **Weighted risk increase** | | **1.1** |

AVS, aortic valve stenosis; AVR, aortic valve replacement; RR, relative risk

### Deterministic Results

**Table S9: Deterministic results**

|  | TAVI costs | CS costs | TAVI QALYs | CS QALYs | Inc. costs | Inc. QALYs | ICER | NMB | NHB |
| --- | --- | --- | --- | --- | --- | --- | --- | --- | --- |
| **BE** | € 34,360 | € 41,529 | 7.41 | 7.22 | -€ 7,169 | 0.19 | Dominant | € 12,862 | 0.43 |
| **CH** | 80,390 CHF | 95,856 CHF | 8.58 | 8.37 | -15,467 CHF | 0.21 | Dominant | 25,744 CHF | 0.51 |
| **DE** | € 38,601 | € 45,042 | 7.41 | 7.27 | -€ 6,441 | 0.14 | Dominant | € 11,507 | 0.33 |
| **ES** | € 40,360 | € 47,945 | 7.17 | 6.99 | -€ 7,586 | 0.18 | Dominant | € 12,940 | 0.43 |
| **FR** | € 33,041 | € 37,809 | 7.18 | 6.99 | -€ 4,768 | 0.19 | Dominant | € 14,171 | 0.28 |
| **IT** | € 47,050 | € 51,913 | 7.71 | 7.49 | -€ 4,863 | 0.21 | Dominant | € 11,306 | 0.38 |
| **NL** | € 50,614 | € 53,398 | 8.23 | 8.05 | -€ 2,784 | 0.18 | Dominant | € 11,615 | 0.23 |
| **SE** | 433,869 SEK | 506,491 SEK | 7.03 | 6.85 | -72,622 SEK | 0.18 | Dominant | 253,582 SEK | 0.25 |
| **UK** | £37,525 | £39,376 | 6.61 | 6.47 | -£1,851 | 0.14 | Dominant | £4,691 | 0.23 |

BE: Belgium; CH: Switzerland; CS: Clinical surveillance; DE: Germany; ES: Spain; FR: France; IT: Italy; NL: the Netherlands; SE: Sweden; UK: United Kingdom; ICER: Incremental cost-effective ratio; NHB: Net health benefit; NMB: Net monetary benefit; QALYs: Quality-adjusted life years

### Scenario Results

**Table S10: Scenario 1: Younger starting age 65**

|  | Inc. Cost | Inc. Life years | Inc. QALYs | ICER | NMB | NHB |
| --- | --- | --- | --- | --- | --- | --- |
| **BE** | -€ 11,153 | 0.34 | 0.45 | Dominant | € 24,770 | 0.83 |
| **CH** | -31,044 CHF | 0.26 | 0.40 | Dominant | 51,044 CHF | 1.02 |
| **DE** | -€ 11,772 | 0.24 | 0.30 | Dominant | € 22,277 | 0.64 |
| **ES** | -€ 16,671 | 0.23 | 0.40 | Dominant | € 28,640 | 0.95 |
| **FR** | -€ 9,967 | 0.29 | 0.41 | Dominant | € 30,314 | 0.61 |
| **IT** | -€ 8,764 | 0.23 | 0.54 | Dominant | € 24,956 | 0.83 |
| **NL** | -€ 6,757 | 0.33 | 0.39 | Dominant | € 26,505 | 0.53 |
| **SE** | -114,458 SEK | 0.25 | 0.39 | Dominant | 508,742 SEK | 0.51 |
| **UK** | -£5,076 | 0.18 | 0.29 | Dominant | £10,949 | 0.55 |

BE: Belgium; CH: Switzerland; DE: Germany; ES: Spain; FR: France; IT: Italy; NL: the Netherlands; SE: Sweden; UK: United Kingdom; ICER: Incremental cost-effective ratio; NHB: Net health benefit; NMB: Net monetary benefit; Inc: Incremental; QALYs: Quality-adjusted life years

**Table S11: Scenario 2: Younger starting age 70**

|  | Inc. Cost | Inc. Life years | Inc. QALYs | ICER | NMB | NHB |
| --- | --- | --- | --- | --- | --- | --- |
| **BE** | -€ 9,188 | 0.26 | 0.32 | Dominant | € 18,727 | 0.62 |
| **CH** | -23,246 CHF | 0.22 | 0.31 | Dominant | 38,615 CHF | 0.77 |
| **DE** | -€ 9,055 | 0.19 | 0.22 | Dominant | € 16,912 | 0.48 |
| **ES** | -€ 11,982 | 0.19 | 0.29 | Dominant | € 20,638 | 0.69 |
| **FR** | -€ 7,291 | 0.23 | 0.30 | Dominant | € 22,211 | 0.44 |
| **IT** | -€ 6,753 | 0.19 | 0.38 | Dominant | € 18,077 | 0.60 |
| **NL** | -€ 4,617 | 0.25 | 0.29 | Dominant | € 18,939 | 0.38 |
| **SE** | -93,303 SEK | 0.20 | 0.29 | Dominant | 382,108 SEK | 0.38 |
| **UK** | -£3,370 | 0.15 | 0.22 | Dominant | £7,763 | 0.39 |

BE: Belgium; CH: Switzerland; DE: Germany; ES: Spain; FR: France; IT: Italy; NL: the Netherlands; SE: Sweden; UK: United Kingdom; ICER: Incremental cost-effective ratio; NHB: Net health benefit; NMB: Net monetary benefit; QALYs: Quality-adjusted life years

Table S12: Scenario 3: Baron (2024) reintervention data

|  | Inc. Cost | Inc. Life years | Inc. QALYs | ICER | NMB | NHB |
| --- | --- | --- | --- | --- | --- | --- |
| **BE** | -€ 7,343 | 0.17 | 0.19 | Dominant | € 13,036 | 0.43 |
| **CH** | -15,924 CHF | 0.16 | 0.21 | Dominant | 26,201 CHF | 0.52 |
| **DE** | -€ 6,656 | 0.13 | 0.14 | Dominant | € 11,722 | 0.33 |
| **ES** | -€ 7,801 | 0.14 | 0.18 | Dominant | € 13,155 | 0.44 |
| **FR** | -€ 5,008 | 0.16 | 0.19 | Dominant | € 14,412 | 0.29 |
| **IT** | -€ 5,132 | 0.13 | 0.21 | Dominant | € 11,576 | 0.39 |
| **NL** | -€ 3,116 | 0.16 | 0.18 | Dominant | € 11,947 | 0.24 |
| **SE** | -75,095 SEK | 0.14 | 0.18 | Dominant | 255,847 SEK | 0.26 |
| **UK** | -£2,013 | 0.11 | 0.14 | Dominant | £4,853 | 0.24 |

BE: Belgium; CH: Switzerland; DE: Germany; ES: Spain; FR: France; IT: Italy; NL: the Netherlands; SE: Sweden; UK: United Kingdom; ICER: Incremental cost-effective ratio; NHB: Net health benefit; NMB: Net monetary benefit; QALYs: Quality-adjusted life years

Table S13: Scenario 4: S3UR valve reintervention data

|  | Inc. Cost | Inc. Life years | Inc. QALYs | ICER | NMB | NHB |
| --- | --- | --- | --- | --- | --- | --- |
| **BE** | -€ 7,363 | 0.17 | 0.19 | Dominant | € 13,056 | 0.44 |
| **CH** | -15,965 CHF | 0.16 | 0.21 | Dominant | 26,241 CHF | 0.52 |
| **DE** | -€ 6,680 | 0.13 | 0.14 | Dominant | € 11,746 | 0.34 |
| **ES** | -€ 7,824 | 0.14 | 0.18 | Dominant | € 13,179 | 0.44 |
| **FR** | -€ 5,023 | 0.16 | 0.19 | Dominant | € 14,427 | 0.29 |
| **IT** | -€ 5,162 | 0.13 | 0.21 | Dominant | € 11,606 | 0.39 |
| **NL** | -€ 3,151 | 0.16 | 0.18 | Dominant | € 11,982 | 0.24 |
| **SE** | -75,354 SEK | 0.14 | 0.18 | Dominant | 256,105 SEK | 0.26 |
| **UK** | -£2,044 | 0.11 | 0.14 | Dominant | £4,885 | 0.24 |

BE: Belgium; CH: Switzerland; DE: Germany; ES: Spain; FR: France; IT: Italy; NL: the Netherlands; SE: Sweden; UK: United Kingdom; ICER: Incremental cost-effective ratio; NHB: Net health benefit; NMB: Net monetary benefit; QALYs: Quality-adjusted life years

**Table S14: Scenario 5: Delay AVR in the clinical surveillance arm (6 months). Impact on costs only**

|  | Inc. Cost | Inc. Life years | Inc. QALYs | ICER | NMB | NHB |
| --- | --- | --- | --- | --- | --- | --- |
| **BE** | -€ 6,713 | 0.17 | 0.19 | Dominant | € 12,406 | 0.41 |
| **CH** | -14,460 CHF | 0.16 | 0.21 | Dominant | 24,736 CHF | 0.49 |
| **DE** | -€ 5,981 | 0.13 | 0.14 | Dominant | € 11,047 | 0.32 |
| **ES** | -€ 7,240 | 0.14 | 0.18 | Dominant | € 12,595 | 0.42 |
| **FR** | -€ 4,348 | 0.16 | 0.19 | Dominant | € 13,752 | 0.28 |
| **IT** | -€ 4,163 | 0.13 | 0.21 | Dominant | € 10,606 | 0.35 |
| **NL** | -€ 2,114 | 0.16 | 0.18 | Dominant | € 10,945 | 0.22 |
| **SE** | -69,399 SEK | 0.14 | 0.18 | Dominant | 250,151 SEK | 0.25 |
| **UK** | -£1,259 | 0.11 | 0.14 | Dominant | £4,100 | 0.20 |

AVR: Aortic valve replacement; BE: Belgium; CH: Switzerland; DE: Germany; ES: Spain; FR: France; IT: Italy; NL: the Netherlands; SE: Sweden; UK: United Kingdom; ICER: Incremental cost-effective ratio; NHB: Net health benefit; NMB: Net monetary benefit; QALYs: Quality-adjusted life years

**Table S15: Scenario 6: Delay AVR in the clinical surveillance arm (12 months). Impact on costs only**

|  | Inc. Cost | Inc. Life years | Inc. QALYs | ICER | NMB | NHB |
| --- | --- | --- | --- | --- | --- | --- |
| **BE** | -€ 6,265 | 0.17 | 0.19 | Dominant | € 11,959 | 0.40 |
| **CH** | -13,469 CHF | 0.16 | 0.21 | Dominant | 23,746 CHF | 0.47 |
| **DE** | -€ 5,527 | 0.13 | 0.14 | Dominant | € 10,593 | 0.30 |
| **ES** | -€ 6,897 | 0.14 | 0.18 | Dominant | € 12,252 | 0.41 |
| **FR** | -€ 3,936 | 0.16 | 0.19 | Dominant | € 13,339 | 0.27 |
| **IT** | -€ 3,476 | 0.13 | 0.21 | Dominant | € 9,920 | 0.33 |
| **NL** | -€ 1,455 | 0.16 | 0.18 | Dominant | € 10,286 | 0.21 |
| **SE** | -66,168 SEK | 0.14 | 0.18 | Dominant | 246,919 SEK | 0.25 |
| **UK** | -£678 | 0.11 | 0.14 | Dominant | £3,519 | 0.18 |

AVR: Aortic valve replacement; BE: Belgium; CH: Switzerland; DE: Germany; ES: Spain; FR: France; IT: Italy; NL: the Netherlands; SE: Sweden; UK: United Kingdom; ICER: Incremental cost-effective ratio; NHB: Net health benefit; NMB: Net monetary benefit; QALYs: Quality-adjusted life years

Table S16: Scenario 7: Employing clinical meta-analysis incident rate ratio (RCT)

|  | Inc. Cost | Inc. Life years | Inc. QALYs | ICER | NMB | NHB |
| --- | --- | --- | --- | --- | --- | --- |
| **BE** | -€ 8,632 | 0.00 | 0.11 | Dominant | € 11,801 | 0.39 |
| **CH** | -19,032 CHF | 0.28 | 0.32 | Dominant | 35,022 CHF | 0.70 |
| **DE** | -€ 8,325 | 0.00 | 0.05 | Dominant | € 10,247 | 0.29 |
| **ES** | -€ 10,199 | 0.00 | 0.11 | Dominant | € 13,587 | 0.45 |
| **FR** | -€ 6,494 | 0.01 | 0.12 | Dominant | € 12,355 | 0.25 |
| **IT** | -€ 6,212 | 0.00 | 0.14 | Dominant | € 10,491 | 0.35 |
| **NL** | -€ 4,020 | 0.00 | 0.06 | Dominant | € 7,127 | 0.14 |
| **SE** | -86,307 SEK | 0.00 | 0.11 | Dominant | 196,599 SEK | 0.20 |
| **UK** | -£2,428 | 0.00 | 0.08 | Dominant | £4,005 | 0.20 |

BE: Belgium; CH: Switzerland; DE: Germany; ES: Spain; FR: France; IT: Italy; NL: the Netherlands; SE: Sweden; UK: United Kingdom; ICER: Incremental cost-effective ratio; NHB: Net health benefit; NMB: Net monetary benefit; QALYs: Quality-adjusted life years

Table S17: Scenario 8: Employing clinical meta-analysis incident rate ratio (Observational)

|  | Inc. Cost | Inc. Life years | Inc. QALYs | ICER | NMB | NHB |
| --- | --- | --- | --- | --- | --- | --- |
| **BE** | -€ 7,470 | 0.00 | 0.02 | Dominant | € 8,050 | 0.27 |
| **CH** | -9,605 CHF | 0.28 | 0.24 | Dominant | 21,530 CHF | 0.43 |
| **DE** | -€ 5,660 | 0.00 | 0.01 | Dominant | € 5,953 | 0.17 |
| **ES** | -€ 4,451 | 0.00 | 0.02 | Dominant | € 5,000 | 0.17 |
| **FR** | -€ 3,238 | 0.01 | 0.03 | Dominant | € 4,691 | 0.09 |
| **IT** | -€ 5,217 | 0.00 | 0.02 | Dominant | € 5,877 | 0.20 |
| **NL** | -€ 1,982 | 0.00 | 0.01 | Dominant | € 2,610 | 0.05 |
| **SE** | -72,508 SEK | 0.00 | 0.02 | Dominant | 91,625 SEK | 0.09 |
| **UK** | -£673 | 0.00 | 0.01 | Dominant | £936 | 0.05 |

BE: Belgium; CH: Switzerland; DE: Germany; ES: Spain; FR: France; IT: Italy; NL: the Netherlands; SE: Sweden; UK: United Kingdom; ICER: Incremental cost-effective ratio; NHB: Net health benefit; NMB: Net monetary benefit; QALYs: Quality-adjusted life years

Table S18: Scenario 9: Employing clinical meta-analysis incident rate ratio (Pooled)

|  | Inc. Cost | Inc. Life years | Inc. QALYs | ICER | NMB | NHB |
| --- | --- | --- | --- | --- | --- | --- |
| **BE** | -€ 8,478 | 0.00 | 0.08 | Dominant | € 10,898 | 0.36 |
| **CH** | -16,506 CHF | 0.28 | 0.30 | Dominant | 31,318 CHF | 0.63 |
| **DE** | -€ 7,687 | 0.00 | 0.04 | Dominant | € 9,138 | 0.26 |
| **ES** | -€ 8,607 | 0.00 | 0.09 | Dominant | € 11,172 | 0.37 |
| **FR** | -€ 5,599 | 0.01 | 0.09 | Dominant | € 10,184 | 0.20 |
| **IT** | -€ 6,074 | 0.00 | 0.11 | Dominant | € 9,305 | 0.31 |
| **NL** | -€ 3,492 | 0.00 | 0.05 | Dominant | € 5,882 | 0.12 |
| **SE** | -83,654 SEK | 0.00 | 0.08 | Dominant | 167,541 SEK | 0.17 |
| **UK** | -£1,940 | 0.00 | 0.06 | Dominant | £3,137 | 0.16 |

BE: Belgium; CH: Switzerland; DE: Germany; ES: Spain; FR: France; IT: Italy; NL: the Netherlands; SE: Sweden; UK: United Kingdom; ICER: Incremental cost-effective ratio; NHB: Net health benefit; NMB: Net monetary benefit; QALYs: Quality-adjusted life years

Table S19: Scenario 10: Employing clinical meta-analysis hazard ratios (EARLY TAVR)

|  | Inc. Cost | Inc. Life years | Inc. QALYs | ICER | NMB | NHB |
| --- | --- | --- | --- | --- | --- | --- |
| **BE** | -€ 8,516 | 0.00 | 0.11 | Dominant | € 11,725 | 0.39 |
| **CH** | -19,708 CHF | 0.11 | 0.19 | Dominant | 29,183 CHF | 0.58 |
| **DE** | -€ 8,267 | 0.00 | 0.06 | Dominant | € 10,214 | 0.29 |
| **ES** | -€ 10,234 | 0.00 | 0.11 | Dominant | € 13,665 | 0.46 |
| **FR** | -€ 6,508 | 0.01 | 0.12 | Dominant | € 12,437 | 0.25 |
| **IT** | -€ 6,117 | 0.00 | 0.14 | Dominant | € 10,452 | 0.35 |
| **NL** | -€ 4,005 | 0.00 | 0.06 | Dominant | € 7,150 | 0.14 |
| **SE** | -85,534 SEK | 0.00 | 0.11 | Dominant | 197,227 SEK | 0.20 |
| **UK** | -£2,440 | 0.00 | 0.08 | Dominant | £4,037 | 0.20 |

BE: Belgium; CH: Switzerland; DE: Germany; ES: Spain; FR: France; IT: Italy; NL: the Netherlands; SE: Sweden; UK: United Kingdom; ICER: Incremental cost-effective ratio; NHB: Net health benefit; NMB: Net monetary benefit; QALYs: Quality-adjusted life years

Table S20: Scenario 11: Employing clinical meta-analysis hazard ratios (meta-analysis RCTs)

|  | Inc. Cost | Inc. Life years | Inc. QALYs | ICER | NMB | NHB |
| --- | --- | --- | --- | --- | --- | --- |
| **BE** | -€ 8,786 | 0.00 | 0.11 | Dominant | € 11,995 | 0.40 |
| **CH** | -19,330 CHF | 0.28 | 0.32 | Dominant | 35,382 CHF | 0.71 |
| **DE** | -€ 8,466 | 0.00 | 0.06 | Dominant | € 10,413 | 0.30 |
| **ES** | -€ 10,342 | 0.00 | 0.11 | Dominant | € 13,773 | 0.46 |
| **FR** | -€ 6,580 | 0.01 | 0.12 | Dominant | € 12,509 | 0.25 |
| **IT** | -€ 6,339 | 0.00 | 0.14 | Dominant | € 10,674 | 0.36 |
| **NL** | -€ 4,098 | 0.00 | 0.06 | Dominant | € 7,244 | 0.14 |
| **SE** | -87,521 SEK | 0.00 | 0.11 | Dominant | 199,214 SEK | 0.20 |
| **UK** | -£2,470 | 0.00 | 0.08 | Dominant | £4,067 | 0.20 |

BE: Belgium; CH: Switzerland; DE: Germany; ES: Spain; FR: France; IT: Italy; NL: the Netherlands; SE: Sweden; UK: United Kingdom; ICER: Incremental cost-effective ratio; NHB: Net health benefit; NMB: Net monetary benefit; QALYs: Quality-adjusted life years

Table S21: Scenario 12: Employing all AVS scenarios

|  | Inc. Cost | Inc. Life years | Inc. QALYs | ICER | NMB | NHB |
| --- | --- | --- | --- | --- | --- | --- |
| **BE** | -€ 13,184 | 1.85 | 1.33 | Dominant | € 53,231 | 1.77 |
| **CH** | -21,917 CHF | 1.55 | 1.29 | Dominant | 86,235 CHF | 1.72 |
| **DE** | -€ 11,331 | 1.54 | 1.26 | Dominant | € 55,491 | 1.59 |
| **ES** | -€ 9,661 | 1.57 | 1.15 | Dominant | € 44,174 | 1.47 |
| **FR** | -€ 6,868 | 1.65 | 1.17 | Dominant | € 65,270 | 1.31 |
| **IT** | -€ 11,458 | 1.55 | 1.29 | Dominant | € 50,217 | 1.67 |
| **NL** | -€ 7,639 | 1.80 | 1.53 | Dominant | € 84,219 | 1.68 |
| **SE** | -126,199 SEK | 1.54 | 1.16 | Dominant | 1,282,043 SEK | 1.28 |
| **UK** | -£4,270 | 1.50 | 1.13 | Dominant | £26,809 | 1.34 |

BE: Belgium; CH: Switzerland; DE: Germany; ES: Spain; FR: France; IT: Italy; NL: the Netherlands; SE: Sweden; UK: United Kingdom; ICER: Incremental cost-effective ratio; NHB: Net health benefit; NMB: Net monetary benefit; QALYs: Quality-adjusted life years

Table S22: Scenario 13: Increase HFH in the clinical surveillance arm

|  | Inc. Cost | Inc. Life years | Inc. QALYs | ICER | NMB | NHB |
| --- | --- | --- | --- | --- | --- | --- |
| **BE** | -€ 13,467 | 0.17 | 0.19 | Dominant | € 19,161 | 0.64 |
| **CH** | -22,518 CHF | 0.16 | 0.21 | Dominant | 32,795 CHF | 0.66 |
| **DE** | -€ 11,100 | 0.13 | 0.14 | Dominant | € 16,166 | 0.46 |
| **ES** | -€ 10,121 | 0.14 | 0.18 | Dominant | € 15,476 | 0.52 |
| **FR** | -€ 6,464 | 0.16 | 0.19 | Dominant | € 15,867 | 0.32 |
| **IT** | -€ 10,064 | 0.13 | 0.21 | Dominant | € 16,508 | 0.55 |
| **NL** | -€ 5,028 | 0.16 | 0.18 | Dominant | € 13,860 | 0.28 |
| **SE** | -119,016 SEK | 0.14 | 0.18 | Dominant | 299,778 SEK | 0.30 |
| **UK** | -£2,622 | 0.11 | 0.14 | Dominant | £5,462 | 0.27 |

BE: Belgium; CH: Switzerland; DE: Germany; ES: Spain; FR: France; HFH: Heart failure-related hospitalisation; IT: Italy; NL: the Netherlands; SE: Sweden; UK: United Kingdom; ICER: Incremental cost-effective ratio; NHB: Net health benefit; NMB: Net monetary benefit; QALYs: Quality-adjusted life years

**Table S23: Scenario 14: Trial based mortality symptom level weightings in the clinical surveillance arm (trial weighting)**

|  | Inc. Cost | Inc. Life years | Inc. QALYs | ICER | NMB | NHB |
| --- | --- | --- | --- | --- | --- | --- |
| **BE** | -€ 3,871 | 1.85 | 1.33 | Dominant | € 43,918 | 1.46 |
| **CH** | -7,918 CHF | 1.55 | 1.29 | Dominant | 72,235 CHF | 1.44 |
| **DE** | -€ 3,030 | 1.54 | 1.26 | Dominant | € 47,189 | 1.35 |
| **ES** | -€ 3,443 | 1.57 | 1.15 | Dominant | € 37,957 | 1.27 |
| **FR** | -€ 1,978 | 1.65 | 1.17 | Dominant | € 60,380 | 1.21 |
| **IT** | -€ 1,471 | 1.55 | 1.29 | Dominant | € 40,229 | 1.34 |
| **NL** | € 119 | 1.80 | 1.53 | € 78 | € 76,461 | 1.53 |
| **SE** | -38,519 SEK | 1.54 | 1.16 | Dominant | 1,194,154 SEK | 1.19 |
| **UK** | £570 | 1.50 | 1.13 | £506 | £21,969 | 1.10 |

BE: Belgium; CH: Switzerland; DE: Germany; ES: Spain; FR: France; IT: Italy; NL: the Netherlands; SE: Sweden; UK: United Kingdom; ICER: Incremental cost-effective ratio; NHB: Net health benefit; NMB: Net monetary benefit; QALYs: Quality-adjusted life years

**Table S24: Scenario 15: Trial-based mortality symptom-level weightings in the clinical surveillance arm (Genereux weighting)**

|  | Inc. Cost | Inc. Life years | Inc. QALYs | ICER | NMB | NHB |
| --- | --- | --- | --- | --- | --- | --- |
| **BE** | -€ 7,226 | 0.38 | 0.38 | Dominant | € 18,714 | 0.62 |
| **CH** | -17,671 CHF | 0.44 | 0.45 | Dominant | 40,268 CHF | 0.81 |
| **DE** | -€ 7,096 | 0.35 | 0.34 | Dominant | € 19,025 | 0.54 |
| **ES** | -€ 9,275 | 0.31 | 0.34 | Dominant | € 19,555 | 0.65 |
| **FR** | -€ 5,892 | 0.33 | 0.34 | Dominant | € 22,828 | 0.46 |
| **IT** | -€ 4,995 | 0.33 | 0.40 | Dominant | € 17,093 | 0.57 |
| **NL** | -€ 3,250 | 0.38 | 0.38 | Dominant | € 22,296 | 0.45 |
| **SE** | -75,169 SEK | 0.31 | 0.34 | Dominant | 413,289 SEK | 0.41 |
| **UK** | -£2,000 | 0.34 | 0.33 | Dominant | £8,621 | 0.43 |

BE: Belgium; CH: Switzerland; DE: Germany; ES: Spain; FR: France; IT: Italy; NL: the Netherlands; SE: Sweden; UK: United Kingdom; ICER: Incremental cost-effective ratio; NHB: Net health benefit; NMB: Net monetary benefit; QALYs: Quality-adjusted life years

**Table S25: Scenario 16: HRQoL impact from pre- and 30 days post-procedure (EARLY TAVR)**

|  | Inc. Cost | Inc. Life years | Inc. QALYs | ICER | NMB | NHB |
| --- | --- | --- | --- | --- | --- | --- |
| **BE** | -€ 7,169 | 0.17 | 0.20 | Dominant | € 13,053 | 0.44 |
| **CH** | -15,467 CHF | 0.16 | 0.21 | Dominant | 25,999 CHF | 0.52 |
| **DE** | -€ 6,441 | 0.13 | 0.15 | Dominant | € 11,685 | 0.33 |
| **ES** | -€ 7,586 | 0.14 | 0.18 | Dominant | € 13,127 | 0.44 |
| **FR** | -€ 4,768 | 0.16 | 0.19 | Dominant | € 14,392 | 0.29 |
| **IT** | -€ 4,863 | 0.13 | 0.22 | Dominant | € 11,492 | 0.38 |
| **NL** | -€ 2,784 | 0.16 | 0.18 | Dominant | € 11,929 | 0.24 |
| **SE** | -72,622 SEK | 0.14 | 0.18 | Dominant | 257,062 SEK | 0.26 |
| **UK** | -£1,851 | 0.11 | 0.15 | Dominant | £4,862 | 0.24 |

BE: Belgium; CH: Switzerland; DE: Germany; ES: Spain; FR: France; HRQoL: health-related quality of life; IT: Italy; NL: the Netherlands; SE: Sweden; UK: United Kingdom; ICER: Incremental cost-effective ratio; NHB: Net health benefit; NMB: Net monetary benefit; QALYs: Quality-adjusted life years

Table S26: Scenario 17: Equal 5+ year event rate across clinical surveillance and TAVI arms

|  | Inc. Cost | Inc. Life years | Inc. QALYs | ICER | NMB | NHB |
| --- | --- | --- | --- | --- | --- | --- |
| **BE** | -€ 4,175 | 0.08 | 0.09 | Dominant | € 6,899 | 0.23 |
| **CH** | -7,602 CHF | 0.07 | 0.09 | Dominant | 12,155 CHF | 0.24 |
| **DE** | -€ 3,657 | 0.06 | 0.07 | Dominant | € 6,121 | 0.17 |
| **ES** | -€ 3,947 | 0.06 | 0.09 | Dominant | € 6,547 | 0.22 |
| **FR** | -€ 2,513 | 0.07 | 0.09 | Dominant | € 6,888 | 0.14 |
| **IT** | -€ 2,352 | 0.06 | 0.10 | Dominant | € 5,452 | 0.18 |
| **NL** | -€ 1,093 | 0.07 | 0.08 | Dominant | € 5,234 | 0.10 |
| **SE** | -47,190 SEK | 0.06 | 0.09 | Dominant | 134,526 SEK | 0.13 |
| **UK** | -£610 | 0.05 | 0.07 | Dominant | £1,995 | 0.10 |

BE: Belgium; CH: Switzerland; DE: Germany; ES: Spain; FR: France; IT: Italy; NL: the Netherlands; SE: Sweden; UK: United Kingdom; CS: Clinical surveillance; ET: Early TAVR; ICER: Incremental cost-effective ratio; NHB: Net health benefit; NMB: Net monetary benefit; QALYs: Quality-adjusted life years

**Table S27: Scenario 18: Conversion to AVR (gen gamma)**

|  | Inc. Cost | Inc. Life years | Inc. QALYs | ICER | NMB | NHB |
| --- | --- | --- | --- | --- | --- | --- |
| **BE** | -€ 7,086 | 0.17 | 0.19 | Dominant | € 12,780 | 0.43 |
| **CH** | -15,280 CHF | 0.16 | 0.21 | Dominant | 25,557 CHF | 0.51 |
| **DE** | -€ 6,352 | 0.13 | 0.14 | Dominant | € 11,418 | 0.33 |
| **ES** | -€ 7,508 | 0.14 | 0.18 | Dominant | € 12,863 | 0.43 |
| **FR** | -€ 4,687 | 0.16 | 0.19 | Dominant | € 14,090 | 0.28 |
| **IT** | -€ 4,737 | 0.13 | 0.21 | Dominant | € 11,181 | 0.37 |
| **NL** | -€ 2,653 | 0.16 | 0.18 | Dominant | € 11,484 | 0.23 |
| **SE** | -71,775 SEK | 0.14 | 0.18 | Dominant | 252,527 SEK | 0.25 |
| **UK** | -£1,743 | 0.11 | 0.14 | Dominant | £4,583 | 0.23 |

AVR: Aortic valve replacement; BE: Belgium; CH: Switzerland; DE: Germany; ES: Spain; FR: France; IT: Italy; NL: the Netherlands; SE: Sweden; UK: United Kingdom; ICER: Incremental cost-effective ratio; NHB: Net health benefit; NMB: Net monetary benefit; QALYs: Quality-adjusted life years

**Table S28: Scenario 19: Conversion to AVR (Gompertz)**

|  | Inc. Cost | Inc. Life years | Inc. QALYs | ICER | NMB | NHB |
| --- | --- | --- | --- | --- | --- | --- |
| **BE** | -€ 7,171 | 0.17 | 0.19 | Dominant | € 12,865 | 0.43 |
| **CH** | -15,473 CHF | 0.16 | 0.21 | Dominant | 25,750 CHF | 0.51 |
| **DE** | -€ 6,444 | 0.13 | 0.14 | Dominant | € 11,510 | 0.33 |
| **ES** | -€ 7,589 | 0.14 | 0.18 | Dominant | € 12,943 | 0.43 |
| **FR** | -€ 4,770 | 0.16 | 0.19 | Dominant | € 14,174 | 0.28 |
| **IT** | -€ 4,867 | 0.13 | 0.21 | Dominant | € 11,310 | 0.38 |
| **NL** | -€ 2,788 | 0.16 | 0.18 | Dominant | € 11,619 | 0.23 |
| **SE** | -72,653 SEK | 0.14 | 0.18 | Dominant | 253,404 SEK | 0.25 |
| **UK** | -£1,854 | 0.11 | 0.14 | Dominant | £4,695 | 0.23 |

AVR: Aortic valve replacement; BE: Belgium; CH: Switzerland; DE: Germany; ES: Spain; FR: France; IT: Italy; NL: the Netherlands; SE: Sweden; UK: United Kingdom; ICER: Incremental cost-effective ratio; NHB: Net health benefit; NMB: Net monetary benefit; QALYs: Quality-adjusted life years

**Table S29: Scenario 20: Conversion to AVR (log logistic)**

|  | Inc. Cost | Inc. Life years | Inc. QALYs | ICER | NMB | NHB |
| --- | --- | --- | --- | --- | --- | --- |
| **BE** | -€ 6,929 | 0.17 | 0.19 | Dominant | € 12,622 | 0.42 |
| **CH** | -14,925 CHF | 0.16 | 0.21 | Dominant | 25,202 CHF | 0.50 |
| **DE** | -€ 6,176 | 0.13 | 0.14 | Dominant | € 11,241 | 0.32 |
| **ES** | -€ 7,348 | 0.14 | 0.18 | Dominant | € 12,702 | 0.42 |
| **FR** | -€ 4,526 | 0.16 | 0.19 | Dominant | € 13,929 | 0.28 |
| **IT** | -€ 4,499 | 0.13 | 0.21 | Dominant | € 10,942 | 0.36 |
| **NL** | -€ 2,394 | 0.16 | 0.18 | Dominant | € 11,225 | 0.22 |
| **SE** | -69,961 SEK | 0.14 | 0.18 | Dominant | 250,713 SEK | 0.25 |
| **UK** | -£1,542 | 0.11 | 0.14 | Dominant | £4,382 | 0.22 |

AVR: Aortic valve replacement; BE: Belgium; CH: Switzerland; DE: Germany; ES: Spain; FR: France; IT: Italy; NL: the Netherlands; SE: Sweden; UK: United Kingdom; ICER: Incremental cost-effective ratio; NHB: Net health benefit; NMB: Net monetary benefit; QALYs: Quality-adjusted life years

**Table S30: Scenario 21: Conversion to AVR (log normal)**

|  | Inc. Cost | Inc. Life years | Inc. QALYs | ICER | NMB | NHB |
| --- | --- | --- | --- | --- | --- | --- |
| **BE** | -€ 7,066 | 0.17 | 0.19 | Dominant | € 12,760 | 0.43 |
| **CH** | -15,235 CHF | 0.16 | 0.21 | Dominant | 25,512 CHF | 0.51 |
| **DE** | -€ 6,330 | 0.13 | 0.14 | Dominant | € 11,396 | 0.33 |
| **ES** | -€ 7,489 | 0.14 | 0.18 | Dominant | € 12,844 | 0.43 |
| **FR** | -€ 4,668 | 0.16 | 0.19 | Dominant | € 14,071 | 0.28 |
| **IT** | -€ 4,708 | 0.13 | 0.21 | Dominant | € 11,151 | 0.37 |
| **NL** | -€ 2,622 | 0.16 | 0.18 | Dominant | € 11,453 | 0.23 |
| **SE** | -71,565 SEK | 0.14 | 0.18 | Dominant | 252,317 SEK | 0.25 |
| **UK** | -£1,717 | 0.11 | 0.14 | Dominant | £4,558 | 0.23 |

AVR: Aortic valve replacement; BE: Belgium; CH: Switzerland; DE: Germany; ES: Spain; FR: France; IT: Italy; NL: the Netherlands; SE: Sweden; UK: United Kingdom; ICER: Incremental cost-effective ratio; NHB: Net health benefit; NMB: Net monetary benefit; QALYs: Quality-adjusted life years

**Table S31: Scenario 22: Conversion to AVR (Weibull)**

|  | Inc. Cost | Inc. Life years | Inc. QALYs | ICER | NMB | NHB |
| --- | --- | --- | --- | --- | --- | --- |
| **BE** | -€ 7,187 | 0.17 | 0.19 | Dominant | € 12,880 | 0.43 |
| **CH** | -15,509 CHF | 0.16 | 0.21 | Dominant | 25,786 CHF | 0.52 |
| **DE** | -€ 6,461 | 0.13 | 0.14 | Dominant | € 11,527 | 0.33 |
| **ES** | -€ 7,604 | 0.14 | 0.18 | Dominant | € 12,959 | 0.43 |
| **FR** | -€ 4,786 | 0.16 | 0.19 | Dominant | € 14,189 | 0.28 |
| **IT** | -€ 4,891 | 0.13 | 0.21 | Dominant | € 11,334 | 0.38 |
| **NL** | -€ 2,813 | 0.16 | 0.18 | Dominant | € 11,644 | 0.23 |
| **SE** | -72,827 SEK | 0.14 | 0.18 | Dominant | 253,579 SEK | 0.25 |
| **UK** | -£1,875 | 0.11 | 0.14 | Dominant | £4,715 | 0.24 |

AVR: Aortic valve replacement; BE: Belgium; CH: Switzerland; DE: Germany; ES: Spain; FR: France; IT: Italy; NL: the Netherlands; SE: Sweden; UK: United Kingdom; ICER: Incremental cost-effective ratio; NHB: Net health benefit; NMB: Net monetary benefit; QALYs: Quality-adjusted life years

Table S32: Scenario 23: Trial-based mortality

|  | Inc. Cost | Inc. Life years | Inc. QALYs | ICER | NMB | NHB |
| --- | --- | --- | --- | --- | --- | --- |
| **BE** | -€ 8,361 | 0.88 | 0.73 | Dominant | € 30,270 | 1.01 |
| **CH** | -19,615 CHF | 0.70 | 0.66 | Dominant | 52,471 CHF | 1.05 |
| **DE** | -€ 9,081 | 0.70 | 0.62 | Dominant | € 30,884 | 0.88 |
| **ES** | -€ 11,592 | 0.70 | 0.60 | Dominant | € 29,634 | 0.99 |
| **FR** | -€ 6,712 | 0.75 | 0.62 | Dominant | € 37,762 | 0.76 |
| **IT** | -€ 5,498 | 0.70 | 0.72 | Dominant | € 27,080 | 0.90 |
| **NL** | -€ 4,287 | 0.88 | 0.82 | Dominant | € 45,498 | 0.91 |
| **SE** | -79,857 SEK | 0.70 | 0.63 | Dominant | 707,599 SEK | 0.71 |
| **UK** | -£2,627 | 0.64 | 0.57 | Dominant | £13,972 | 0.70 |

BE: Belgium; CH: Switzerland; DE: Germany; ES: Spain; FR: France; IT: Italy; NL: the Netherlands; SE: Sweden; UK: United Kingdom; ICER: Incremental cost-effective ratio; NHB: Net health benefit; NMB: Net monetary benefit; QALYs: Quality-adjusted life years

**Table S33: Scenario 24: Trial-based mortality, general population adjustment**

|  | Inc. Cost | Inc. Life years | Inc. QALYs | ICER | NMB | NHB |
| --- | --- | --- | --- | --- | --- | --- |
| **BE** | -€ 7,932 | 0.00 | 0.11 | Dominant | € 11,223 | 0.37 |
| **CH** | -18,977 CHF | 0.19 | 0.25 | Dominant | 31,600 CHF | 0.63 |
| **DE** | -€ 7,891 | 0.00 | 0.06 | Dominant | € 9,884 | 0.28 |
| **ES** | -€ 10,165 | 0.00 | 0.12 | Dominant | € 13,680 | 0.46 |
| **FR** | -€ 6,445 | 0.01 | 0.12 | Dominant | € 12,516 | 0.25 |
| **IT** | -€ 5,624 | 0.00 | 0.15 | Dominant | € 10,069 | 0.34 |
| **NL** | -€ 3,849 | 0.00 | 0.06 | Dominant | € 7,075 | 0.14 |
| **SE** | -81,313 SEK | 0.00 | 0.11 | Dominant | 195,755 SEK | 0.20 |
| **UK** | -£2,423 | 0.00 | 0.08 | Dominant | £4,057 | 0.20 |

BE: Belgium; CH: Switzerland; DE: Germany; ES: Spain; FR: France; IT: Italy; NL: the Netherlands; SE: Sweden; UK: United Kingdom; ICER: Incremental cost-effective ratio; NHB: Net health benefit; NMB: Net monetary benefit; QALYs: Quality-adjusted life years

**Table S34: Scenario 25: Mortality - trial based approach for the first 5 years, then health state specific values onwards**

|  | Inc. Cost | Inc. Life years | Inc. QALYs | ICER | NMB | NHB |
| --- | --- | --- | --- | --- | --- | --- |
| **BE** | -€ 7,321 | 0.26 | 0.26 | Dominant | € 15,201 | 0.51 |
| **CH** | -15,218 CHF | 0.25 | 0.27 | Dominant | 28,949 CHF | 0.58 |
| **DE** | -€ 6,686 | 0.21 | 0.21 | Dominant | € 14,132 | 0.40 |
| **ES** | -€ 7,821 | 0.22 | 0.24 | Dominant | € 15,105 | 0.50 |
| **FR** | -€ 4,814 | 0.25 | 0.25 | Dominant | € 17,382 | 0.35 |
| **IT** | -€ 4,878 | 0.21 | 0.29 | Dominant | € 13,466 | 0.45 |
| **NL** | -€ 2,896 | 0.25 | 0.26 | Dominant | € 15,736 | 0.31 |
| **SE** | -72,611 SEK | 0.22 | 0.24 | Dominant | 316,752 SEK | 0.32 |
| **UK** | -£1,899 | 0.19 | 0.20 | Dominant | £5,984 | 0.30 |

BE: Belgium; CH: Switzerland; DE: Germany; ES: Spain; FR: France; IT: Italy; NL: the Netherlands; SE: Sweden; UK: United Kingdom; ICER: Incremental cost-effective ratio; NHB: Net health benefit; NMB: Net monetary benefit; QALYs: Quality-adjusted life years

**Table S35: Scenario 26: Increased PVS and AVS cost**

|  | Inc. Cost | Inc. Life years | Inc. QALYs | ICER | NMB | NHB |
| --- | --- | --- | --- | --- | --- | --- |
| **BE** | -€ 10,374 | 0.17 | 0.19 | Dominant | € 16,068 | 0.54 |
| **CH** | -22,757 CHF | 0.16 | 0.21 | Dominant | 33,034 CHF | 0.66 |
| **DE** | -€ 10,269 | 0.13 | 0.14 | Dominant | € 15,335 | 0.44 |
| **ES** | -€ 11,431 | 0.14 | 0.18 | Dominant | € 16,786 | 0.56 |
| **FR** | -€ 8,099 | 0.16 | 0.19 | Dominant | € 17,502 | 0.35 |
| **IT** | -€ 9,884 | 0.13 | 0.21 | Dominant | € 16,327 | 0.54 |
| **NL** | -€ 8,498 | 0.16 | 0.18 | Dominant | € 17,329 | 0.35 |
| **SE** | -115,875 SEK | 0.14 | 0.18 | Dominant | 296,627 SEK | 0.30 |
| **UK** | -£6,065 | 0.11 | 0.14 | Dominant | £8,906 | 0.45 |

AVS: Acute valve syndrome; BE: Belgium; CH: Switzerland; DE: Germany; ES: Spain; FR: France; IT: Italy; NL: the Netherlands; PVS: Progressive valve syndrome; SE: Sweden; UK: United Kingdom; ICER: Incremental cost-effective ratio; NHB: Net health benefit; NMB: Net monetary benefit; QALYs: Quality-adjusted life years

**Table S36: Scenario 27: HRQoL impact from PPM implant**

|  | Inc. Cost | Inc. Life years | Inc. QALYs | ICER | NMB | NHB |
| --- | --- | --- | --- | --- | --- | --- |
| **BE** | -€ 7,169 | 0.17 | 0.19 | Dominant | € 12,869 | 0.43 |
| **CH** | -15,467 CHF | 0.16 | 0.21 | Dominant | 25,754 CHF | 0.52 |
| **DE** | -€ 6,441 | 0.13 | 0.14 | Dominant | € 11,514 | 0.33 |
| **ES** | -€ 7,586 | 0.14 | 0.18 | Dominant | € 12,947 | 0.43 |
| **FR** | -€ 4,768 | 0.16 | 0.19 | Dominant | € 14,182 | 0.28 |
| **IT** | -€ 4,863 | 0.13 | 0.21 | Dominant | € 11,313 | 0.38 |
| **NL** | -€ 2,784 | 0.16 | 0.18 | Dominant | € 11,626 | 0.23 |
| **SE** | -72,622 SEK | 0.14 | 0.18 | Dominant | 253,582 SEK | 0.25 |
| **UK** | -£1,851 | 0.11 | 0.14 | Dominant | £4,695 | 0.23 |

BE: Belgium; CH: Switzerland; DE: Germany; ES: Spain; FR: France; HRQoL: health-related quality of life; IT: Italy; NL: the Netherlands; Permanent Pacemaker Implant; SE: Sweden; UK: United Kingdom; ICER: Incremental cost-effective ratio; NHB: Net health benefit; NMB: Net monetary benefit; QALYs: Quality-adjusted life years

Table S37: Scenario 28: Employing five-year time horizon

|  | Inc. Cost | Inc. Life years | Inc. QALYs | ICER | NMB | NHB |
| --- | --- | --- | --- | --- | --- | --- |
| **BE** | -€ 5,690 | 0.00 | 0.02 | Dominant | € 6,141 | 0.20 |
| **CH** | -8,530 CHF | 0.00 | 0.01 | Dominant | 9,176 CHF | 0.18 |
| **DE** | -€ 5,205 | 0.00 | 0.01 | Dominant | € 5,553 | 0.16 |
| **ES** | -€ 4,619 | 0.00 | 0.02 | Dominant | € 5,118 | 0.17 |
| **FR** | -€ 3,375 | 0.00 | 0.01 | Dominant | € 4,092 | 0.08 |
| **IT** | -€ 5,397 | 0.00 | 0.02 | Dominant | € 5,989 | 0.20 |
| **NL** | -€ 4,520 | 0.00 | 0.01 | Dominant | € 5,034 | 0.10 |
| **SE** | -62,307 SEK | 0.00 | 0.02 | Dominant | 78,007 SEK | 0.08 |
| **UK** | -£2,489 | 0.00 | 0.01 | Dominant | £2,747 | 0.14 |

BE: Belgium; CH: Switzerland; DE: Germany; ES: Spain; FR: France; IT: Italy; NL: the Netherlands; SE: Sweden; UK: United Kingdom; ICER: Incremental cost-effective ratio; NHB: Net health benefit; NMB: Net monetary benefit; QALYs: Quality-adjusted life years

## Figures

### Time to conversion

Figure S1: Time to conversion to AVR over 10 years in the CS arm

### AVR, aortic valve replacement

### Results: Cost Breakdowns

Figure S2a: Belgium deterministic cost breakdown for first ten years of the model
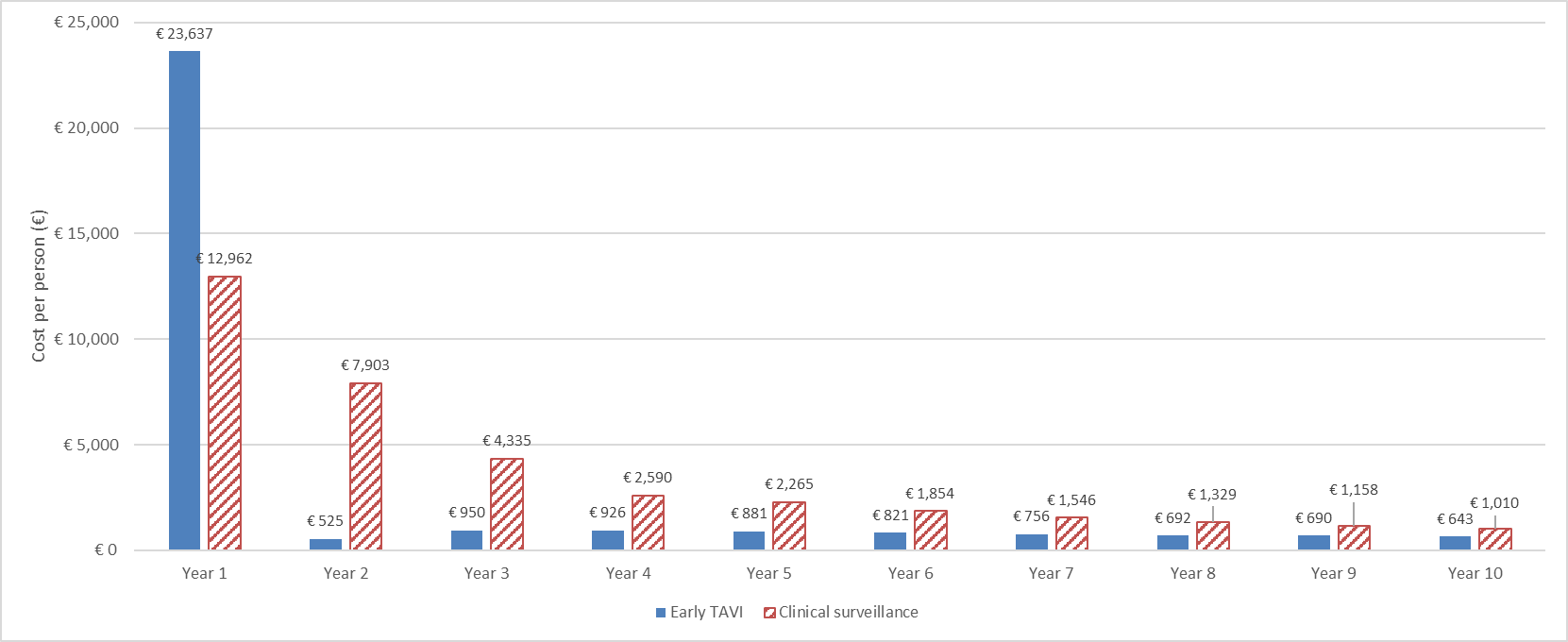


Figure S2b: Belgium deterministic cumulative costs per person


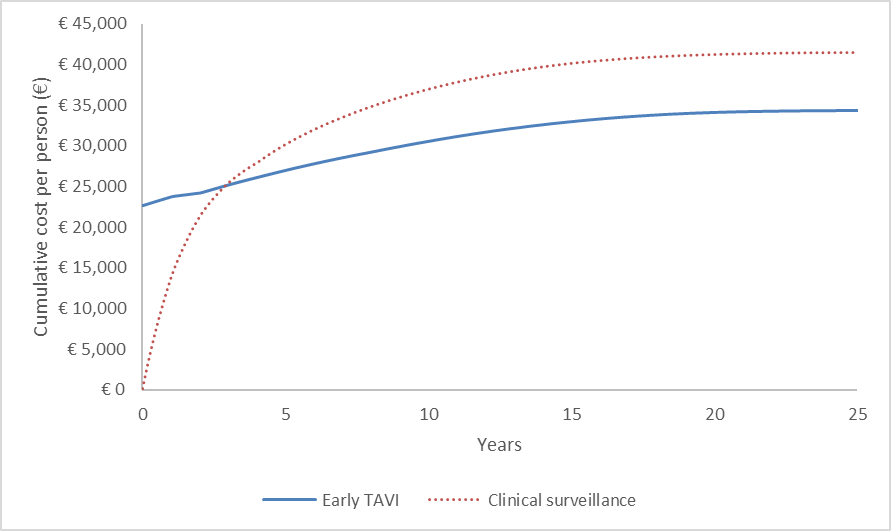


Figure S2c: Belgium deterministic cost breakdown by category per person


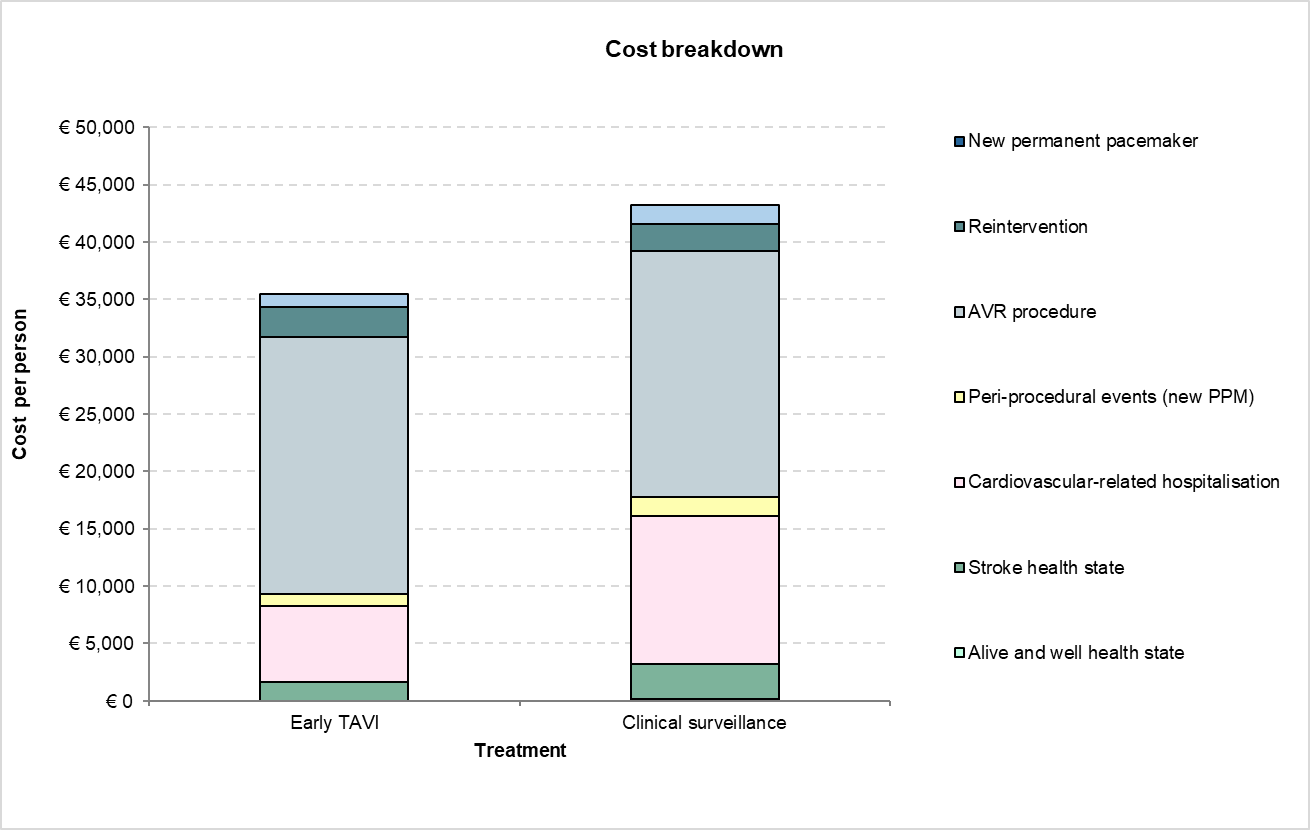


Figure S3a: Switzerland deterministic cost breakdown for first ten years of the model


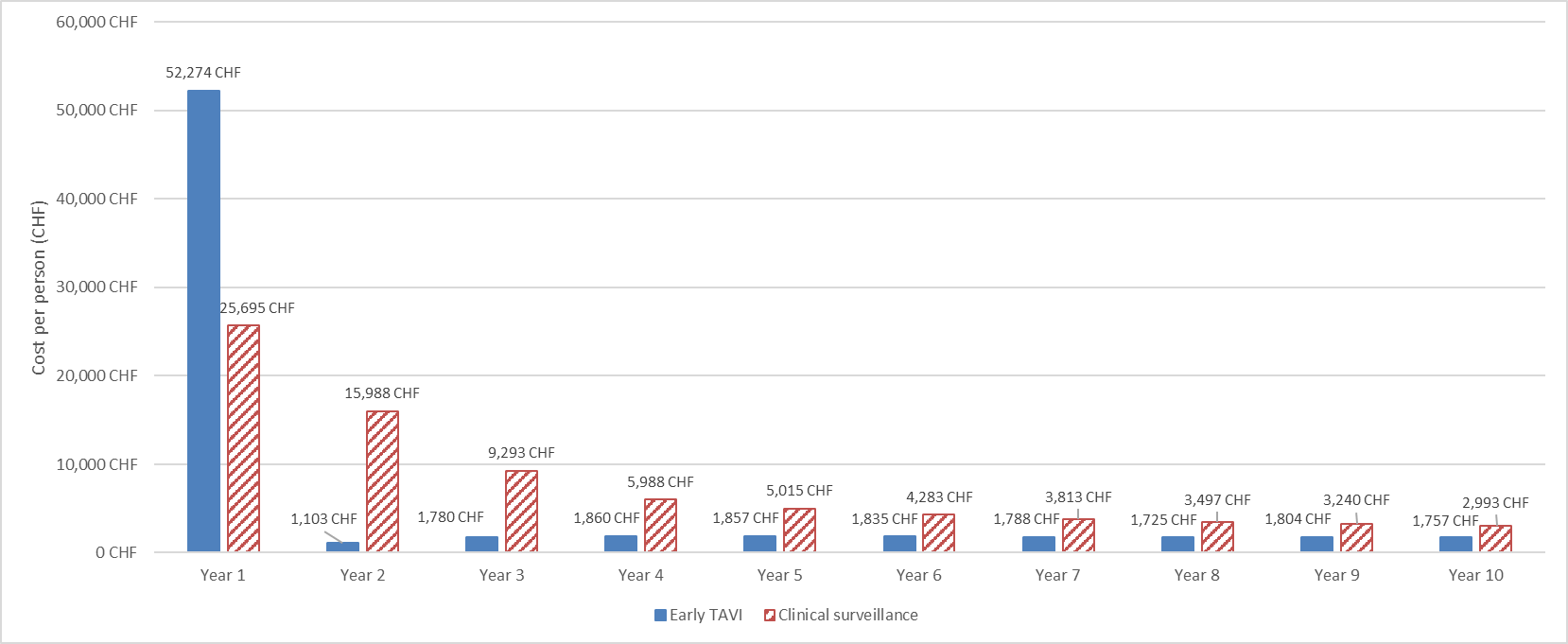


Figure S3b: Switzerland deterministic cumulative costs per person


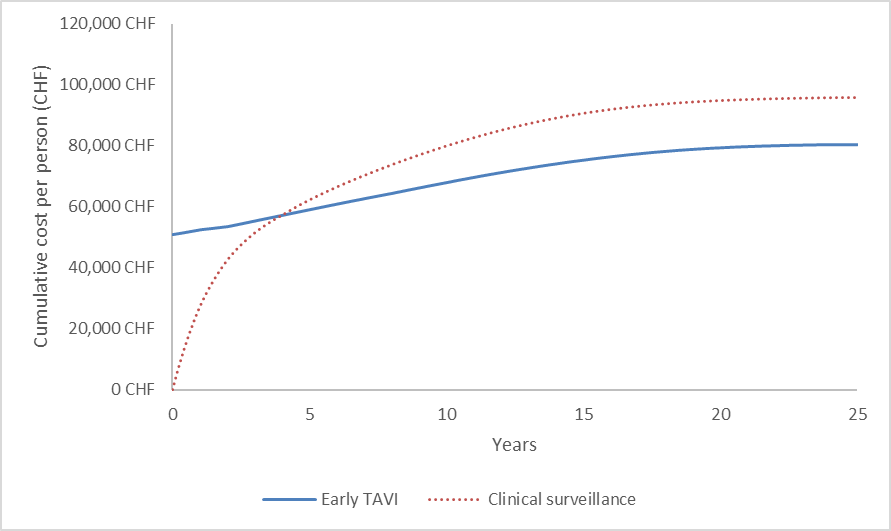


Figure S3c: Switzerland deterministic cost breakdown by category per person


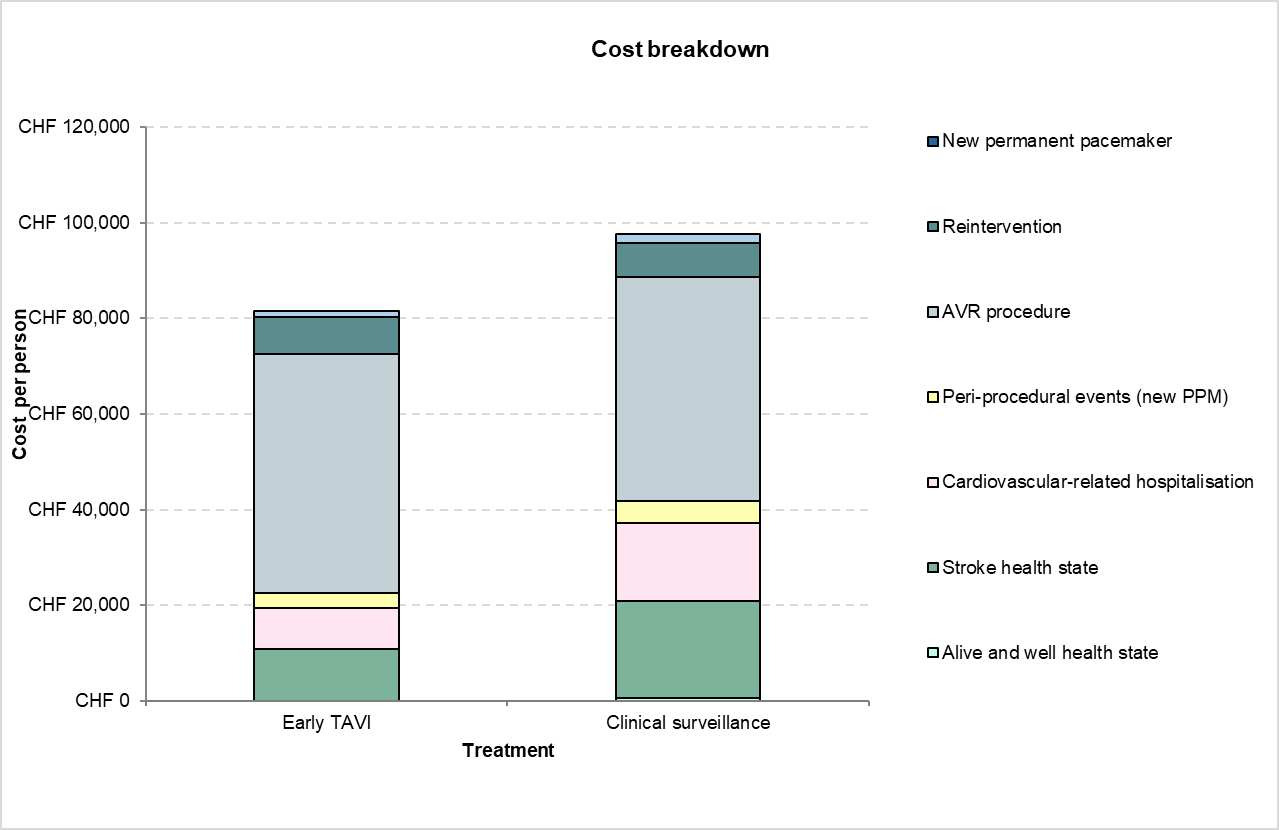


Figure S4a: Germany deterministic cost breakdown for first ten years of the model


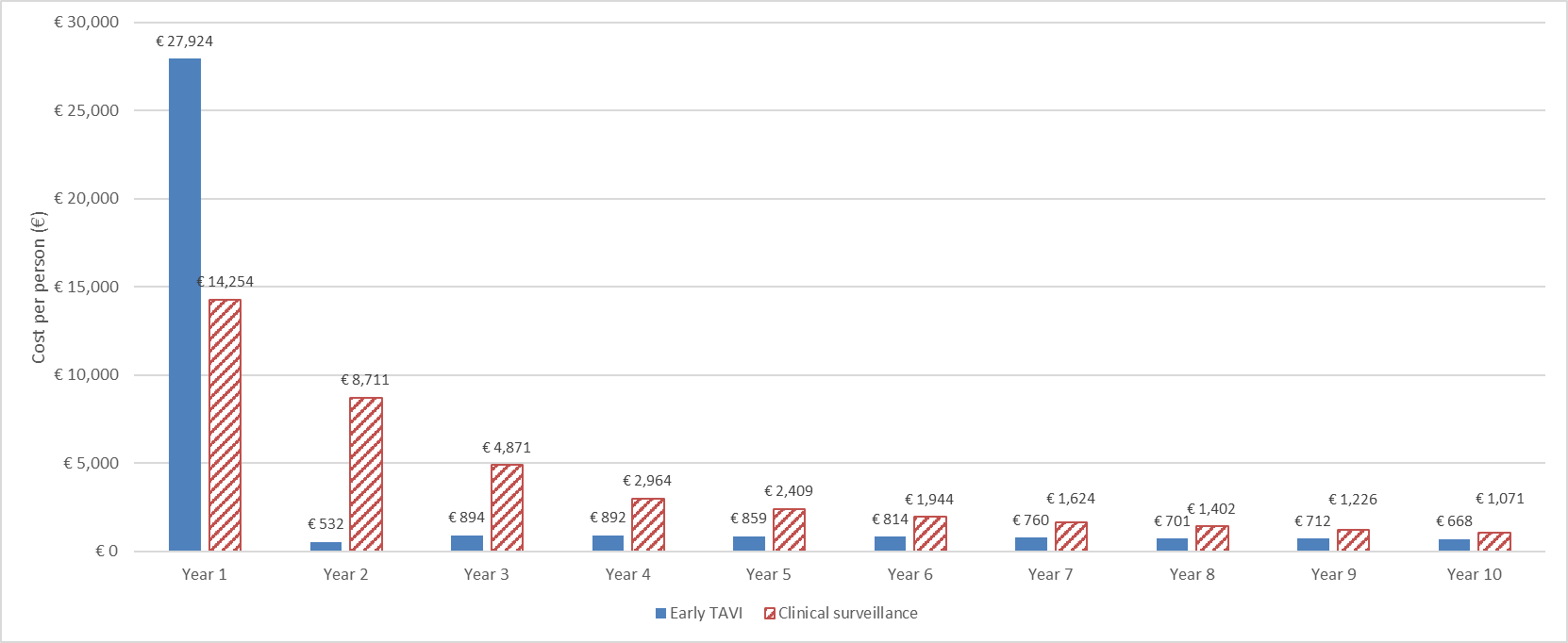


Figure S4b: Germany deterministic cumulative costs per person


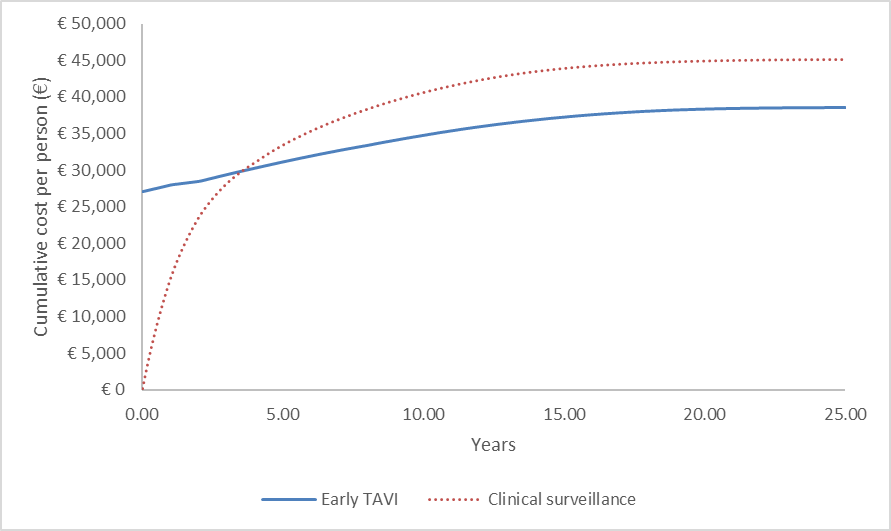


Figure S4c: Germany deterministic cost breakdown by category per person


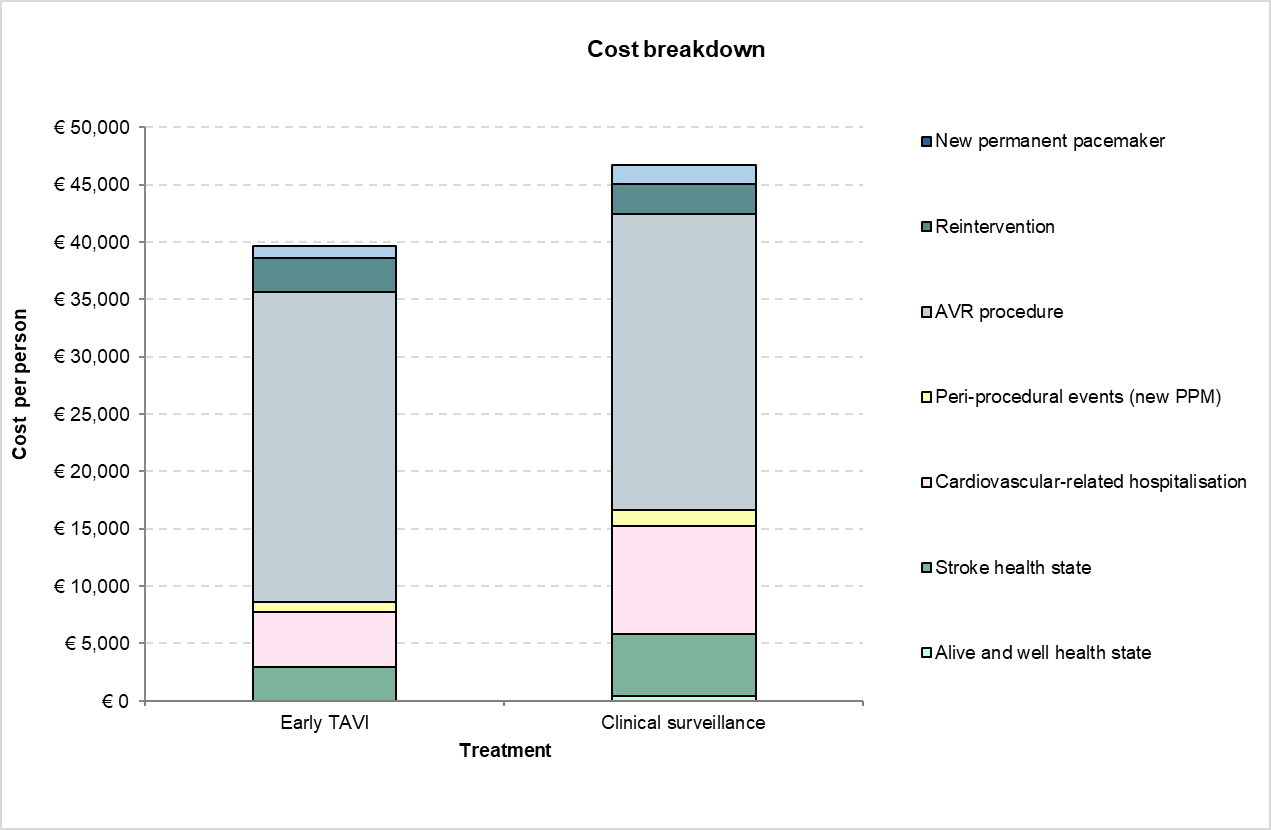


Figure S5a: Spain deterministic cost breakdown for first ten years of the model


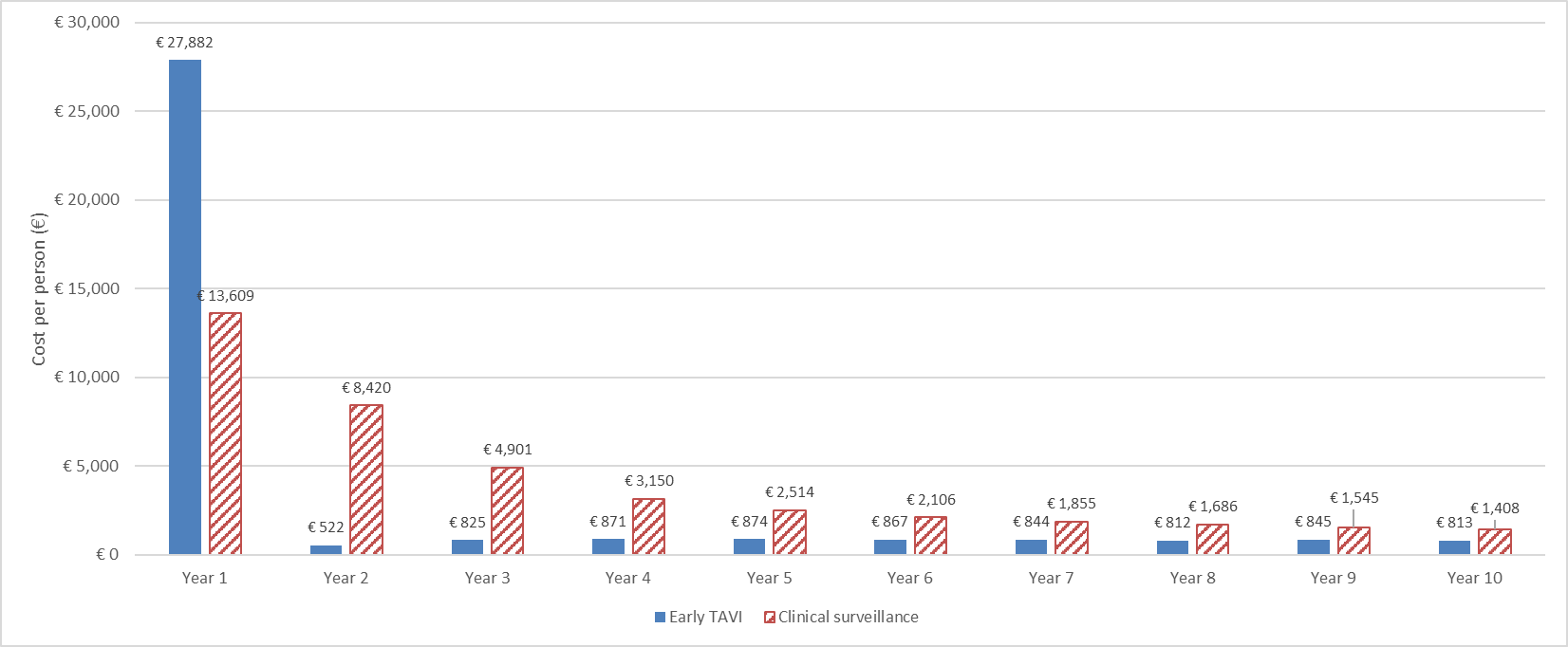


Figure S5b: Spain deterministic cumulative costs per person


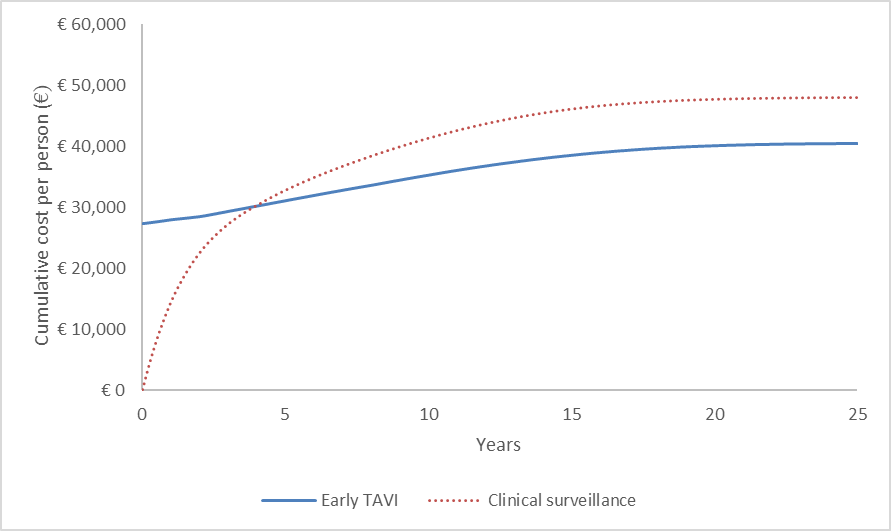


Figure S5c: Spain deterministic cost breakdown by category per person


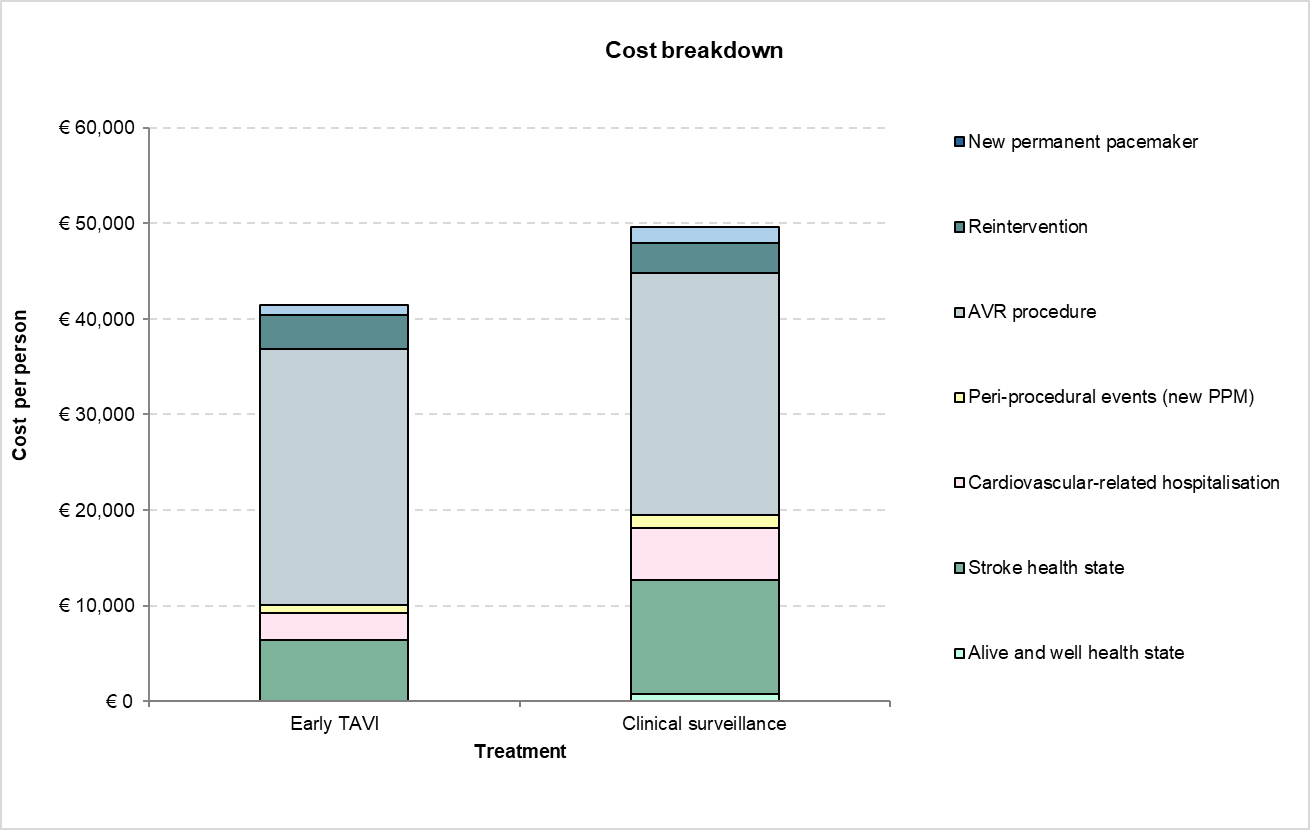


Figure S6a: France deterministic cost breakdown for first ten years of the model


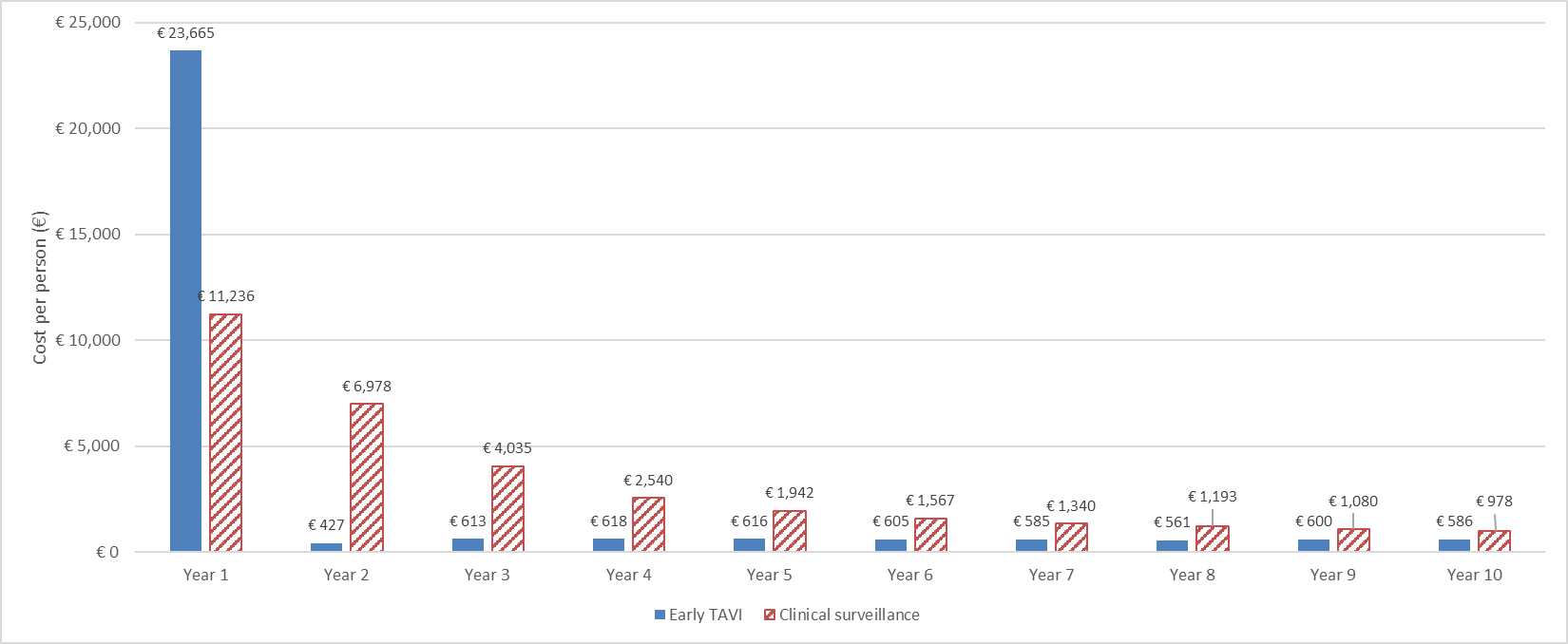


Figure S6b: France deterministic cumulative costs per person


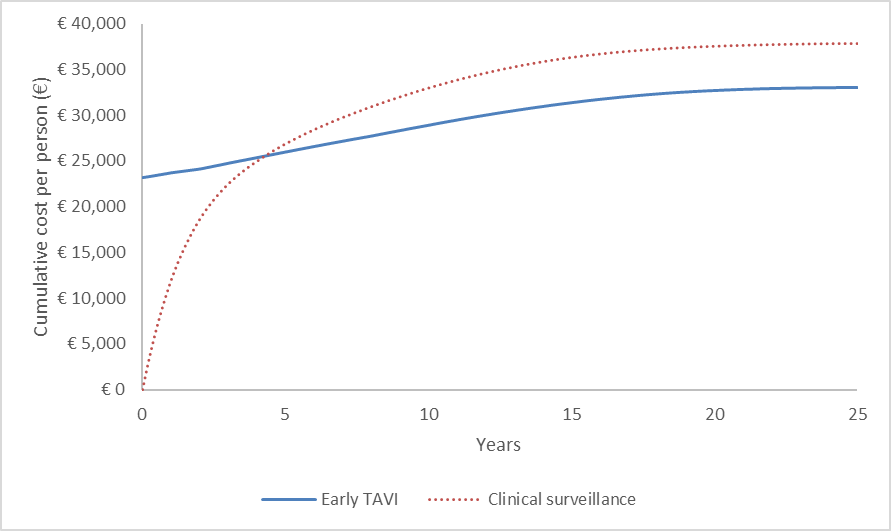


Figure S6c: France deterministic cost breakdown by category per person


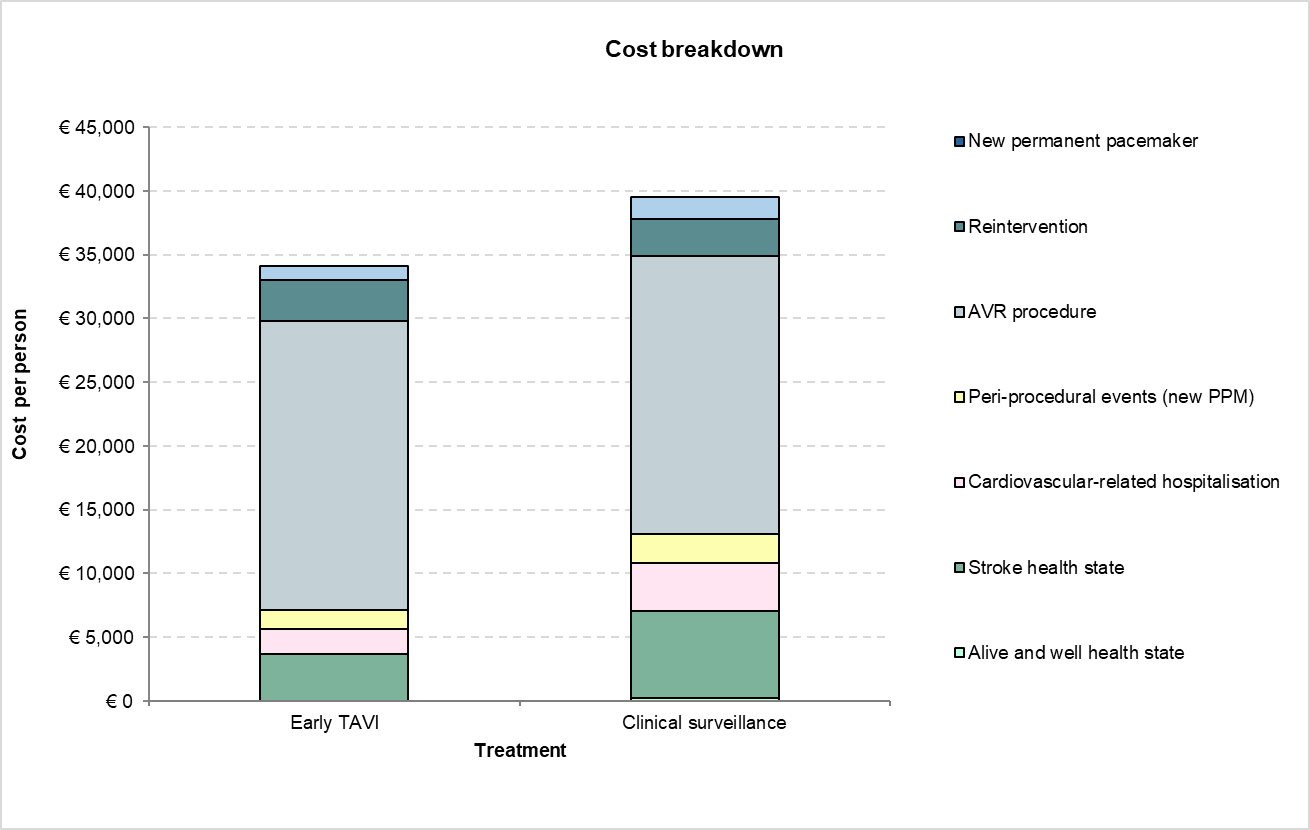


Figure S7a: Italy deterministic cost breakdown for first ten years of the model


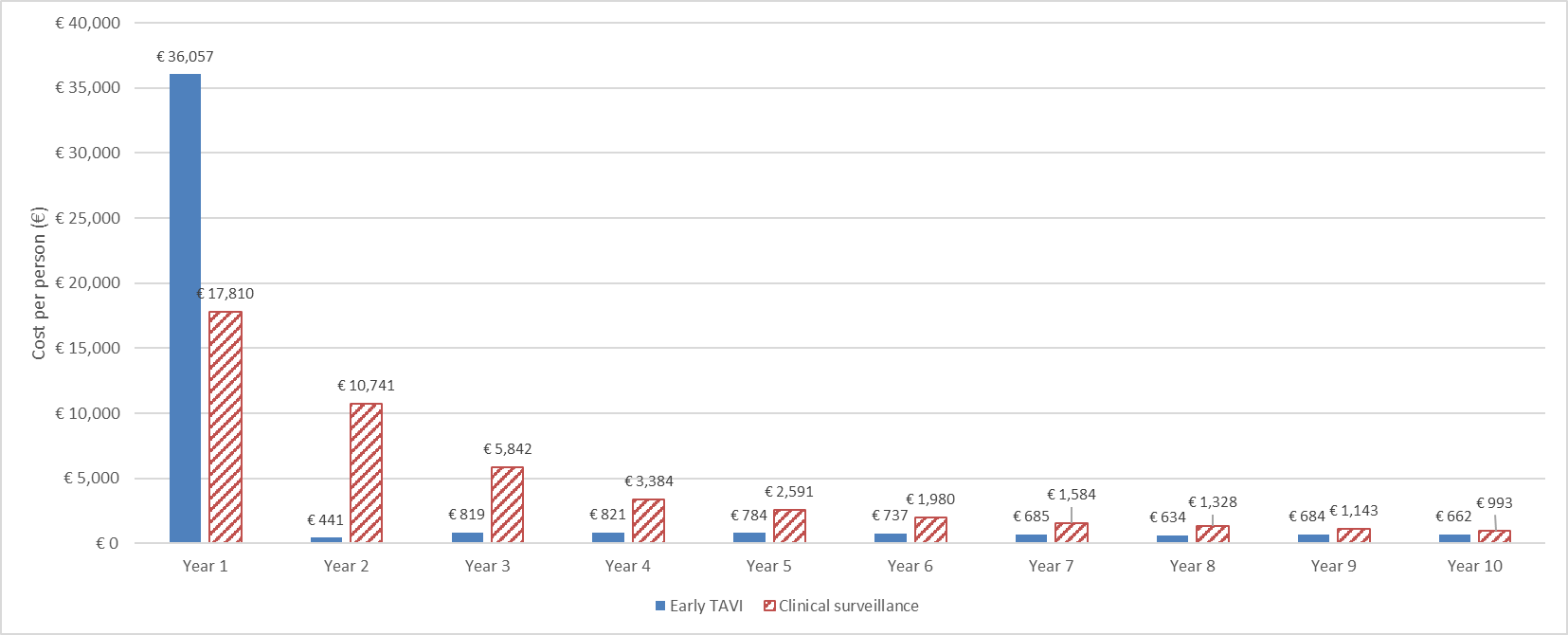


Figure S7b: Italy deterministic cumulative costs per person


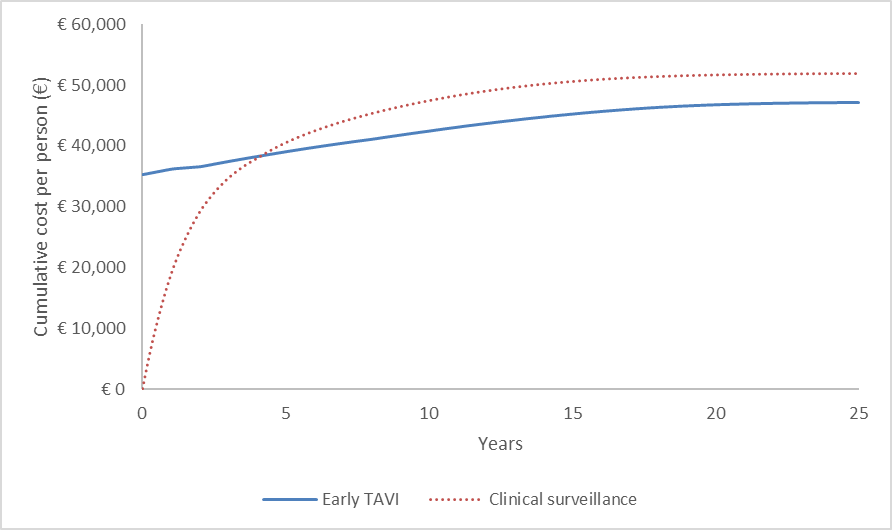


Figure S7c: Italy deterministic cost breakdown by category per person


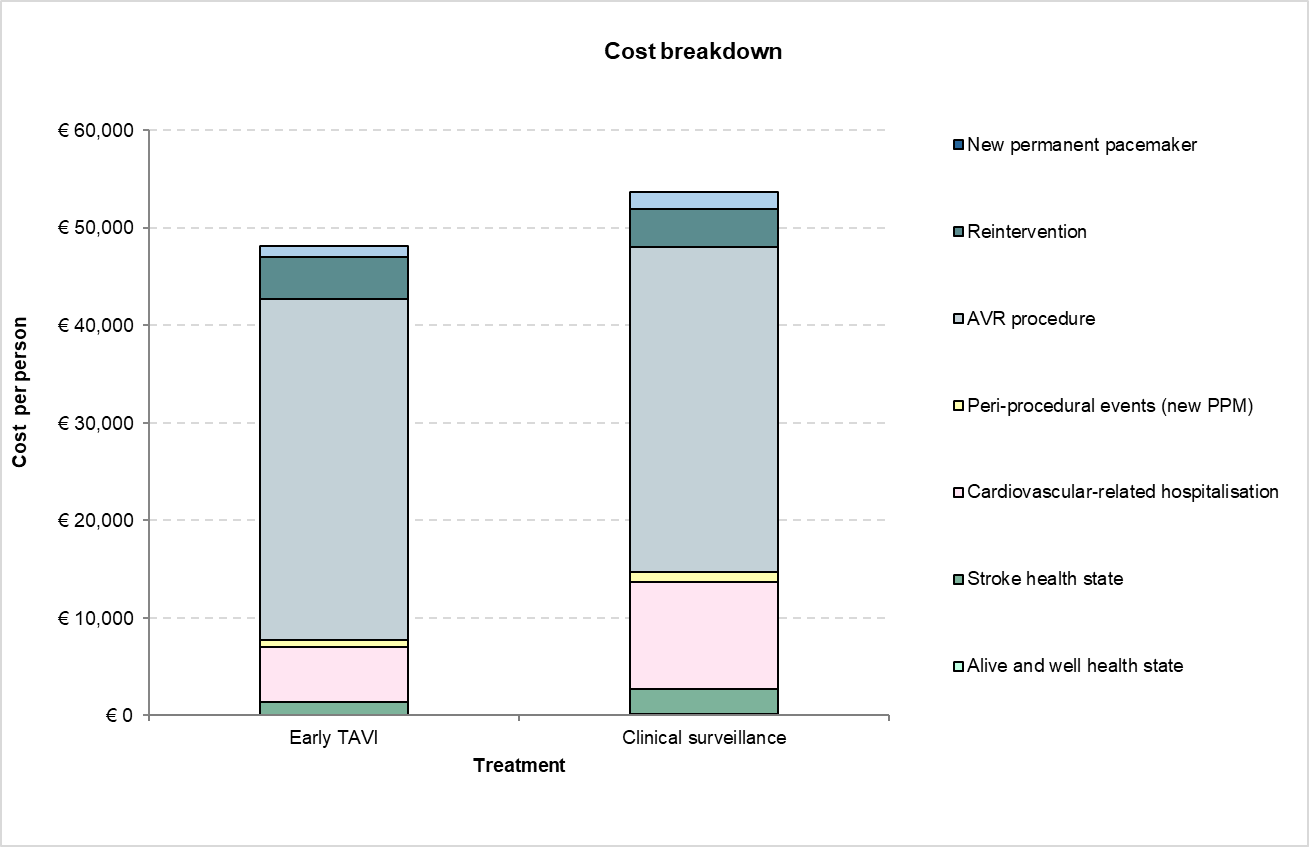


Figure S8a: The Netherlands deterministic cost breakdown for first ten years of the model


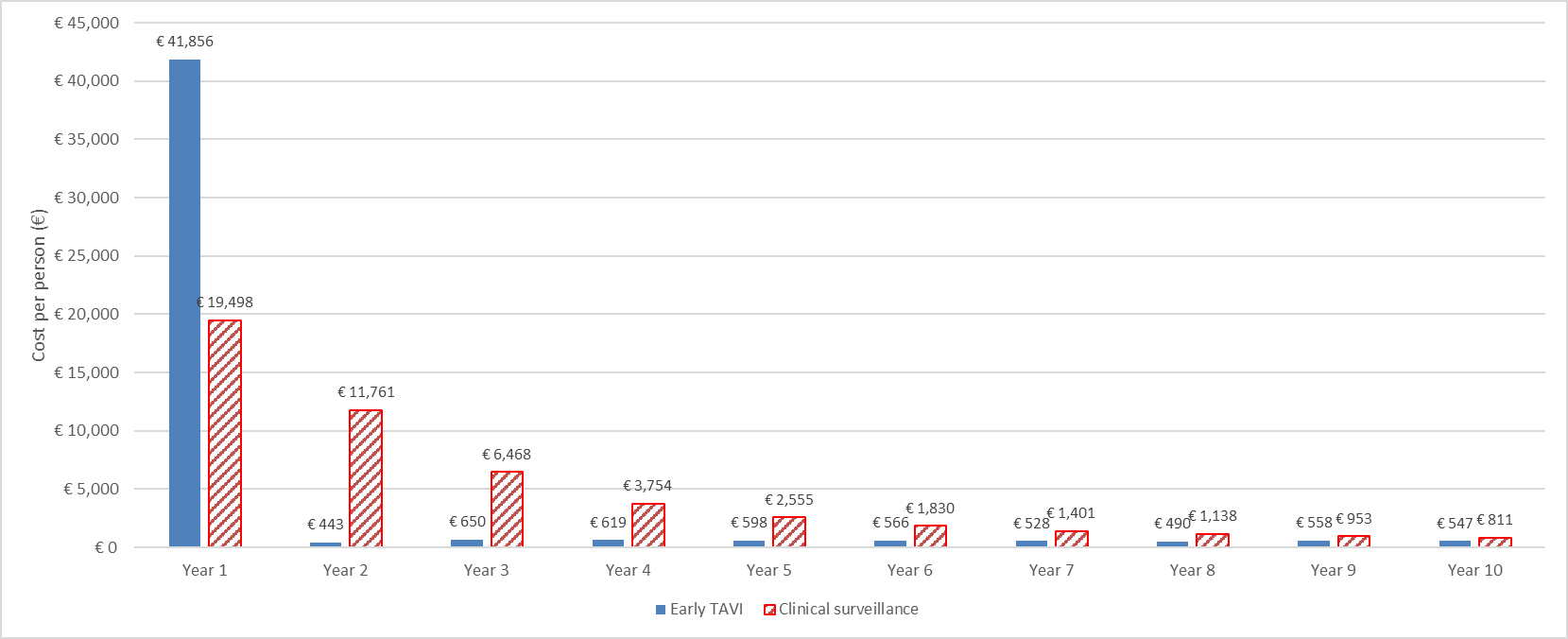


Figure S8b: The Netherlands deterministic cumulative costs per person


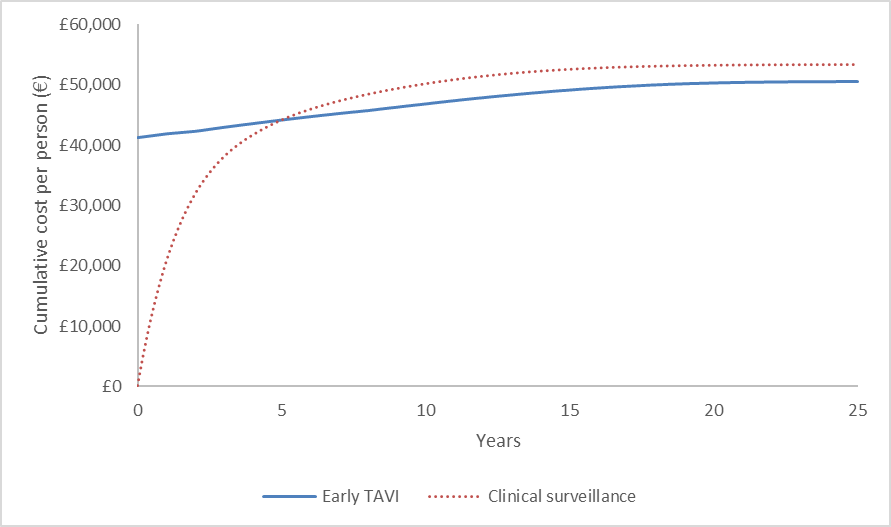


Figure S8c: The Netherlands deterministic cost breakdown by category per person


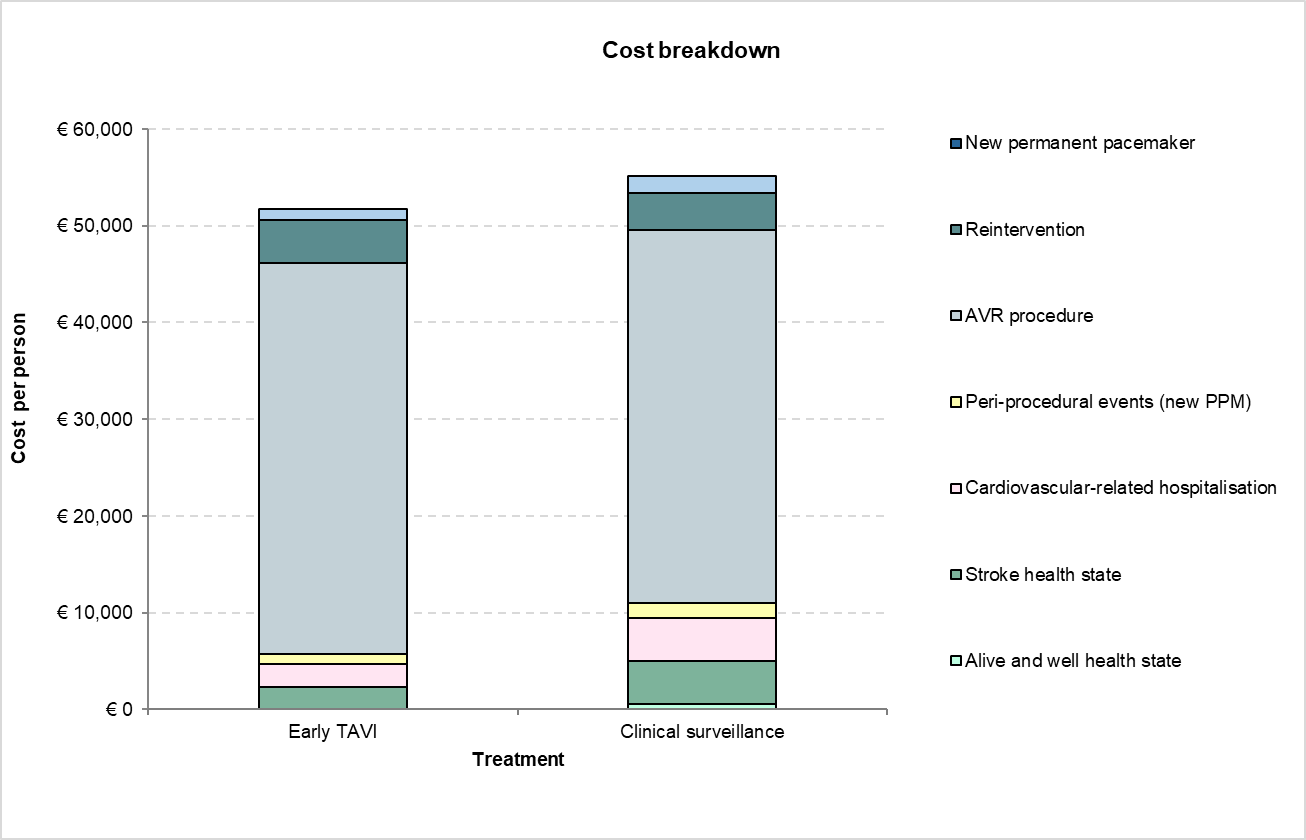


Figure S9a: Sweden deterministic cost breakdown for first ten years of the model


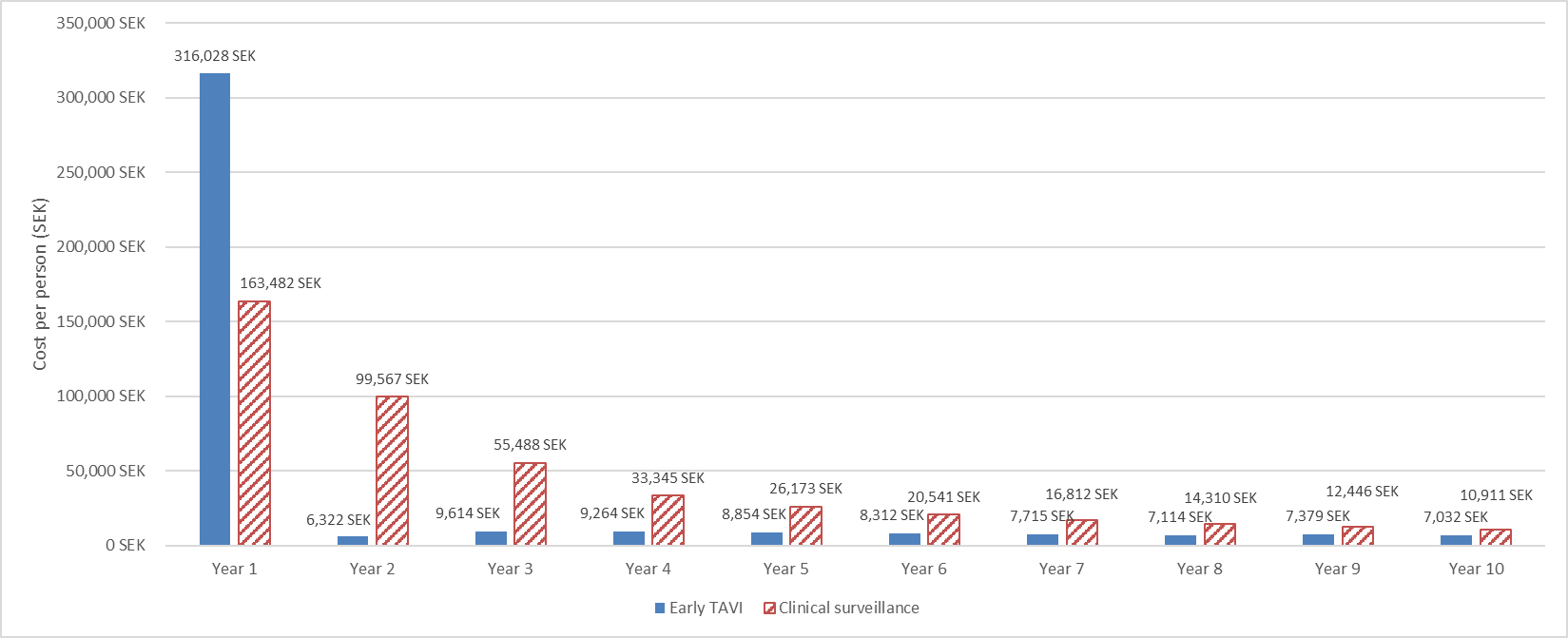


Figure S9b: Sweden deterministic cumulative costs per person


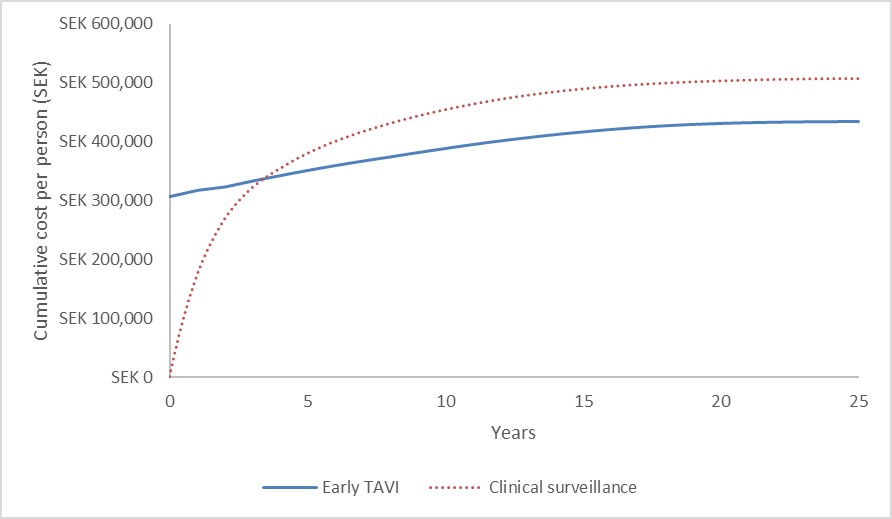


Figure S9c: Sweden deterministic cost breakdown by category per person


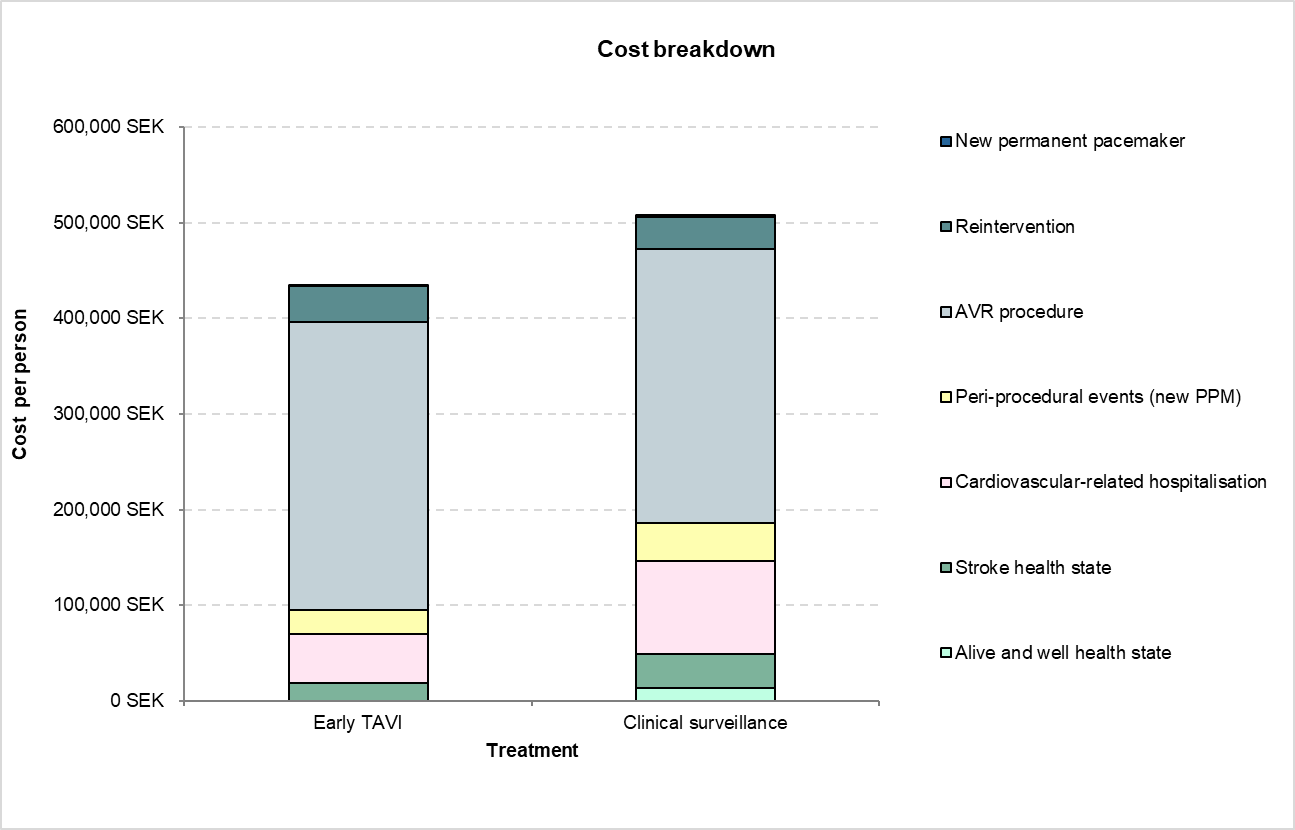


Figure S10a: UK deterministic cost breakdown for first ten years of the model


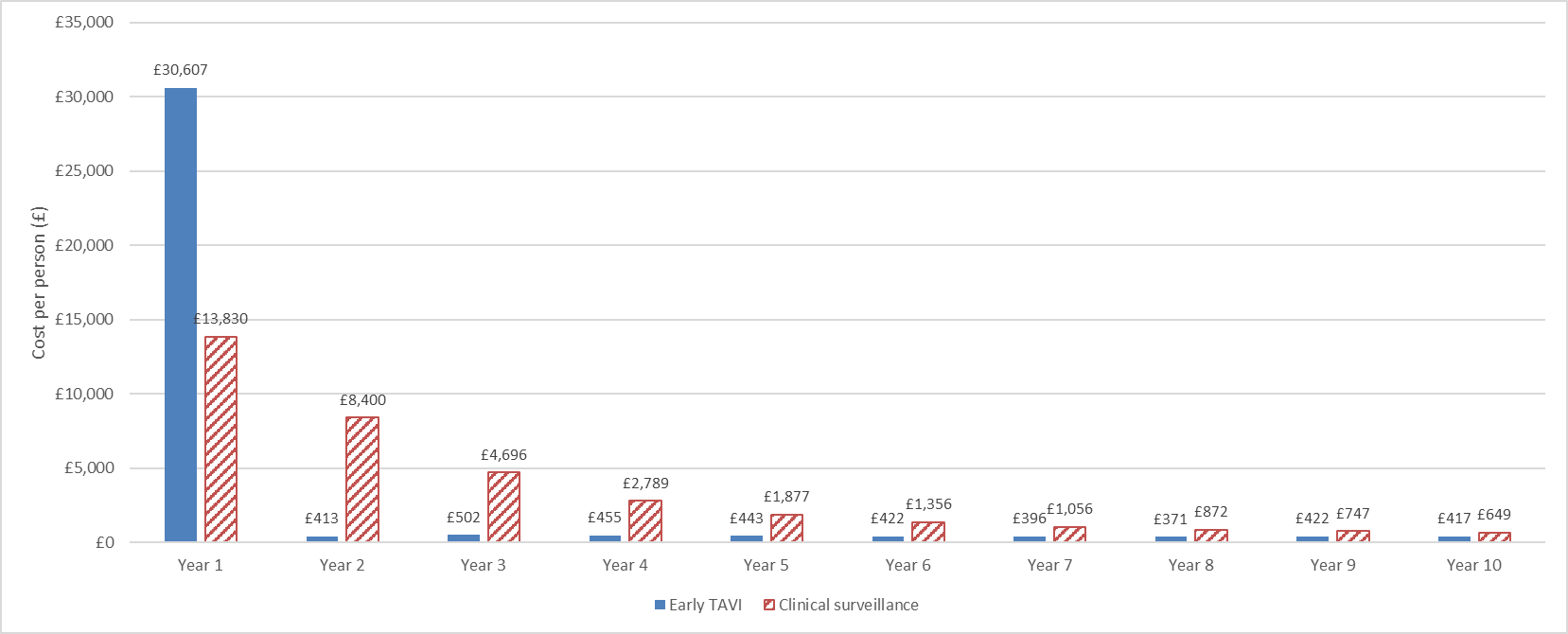


Figure S10b: UK deterministic cumulative costs per person


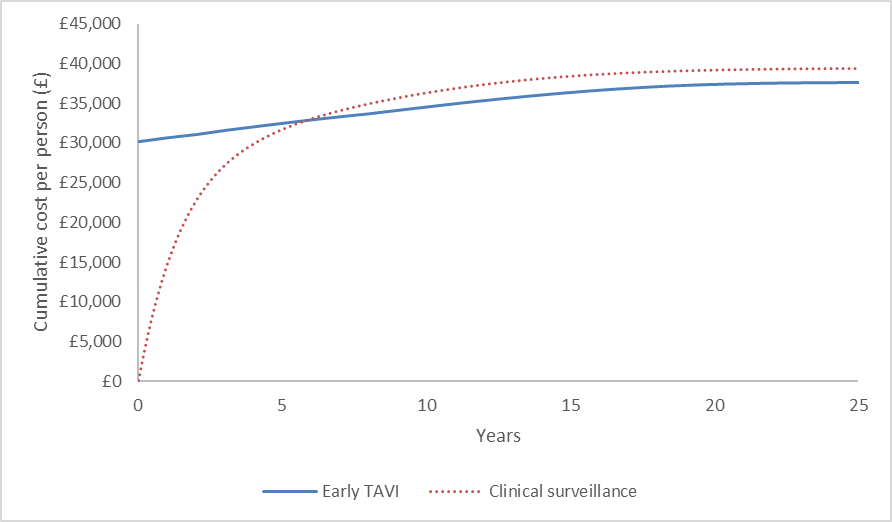


Figure S10c: UK deterministic cost breakdown by category per person


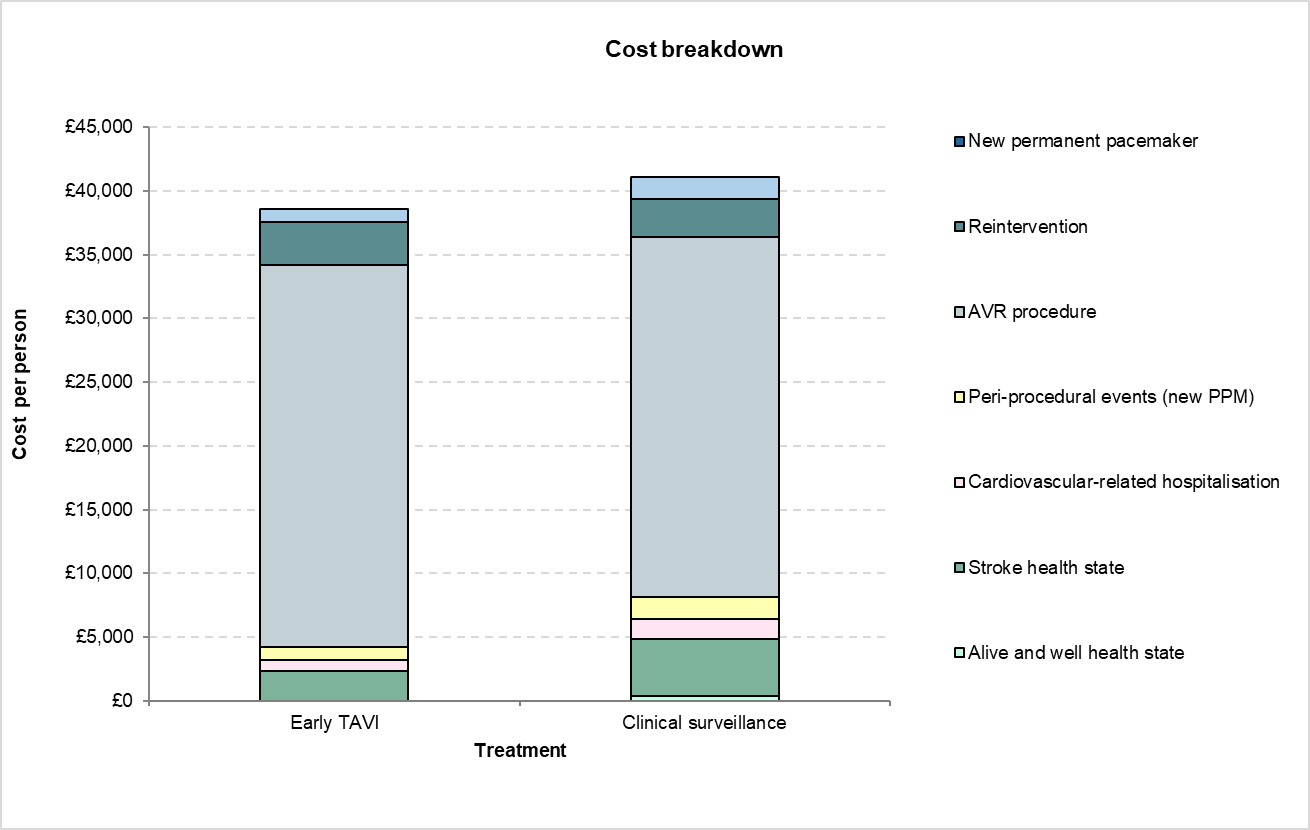


### Cost-effectiveness planes (CEP)

Figure S11: Belgium CEP


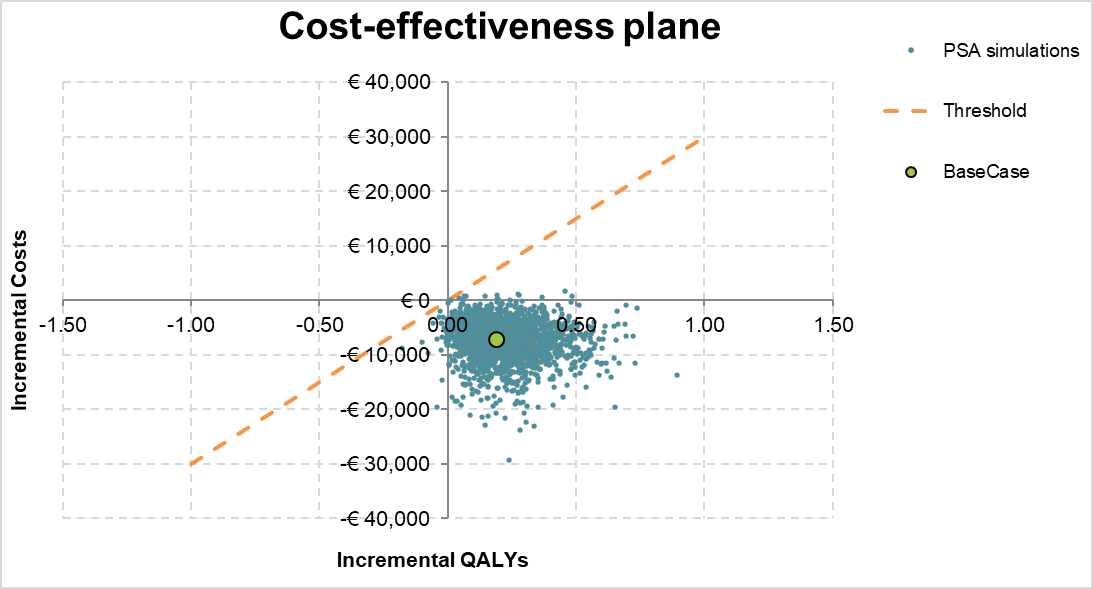


Figure S12: Switzerland CEP


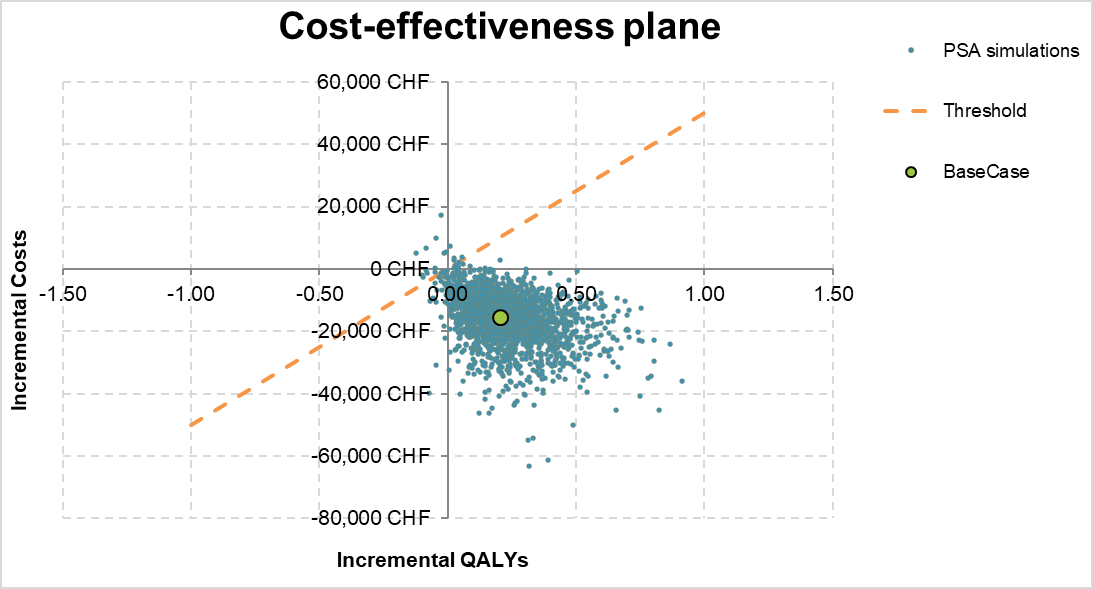


Figure S13: Germany CEP


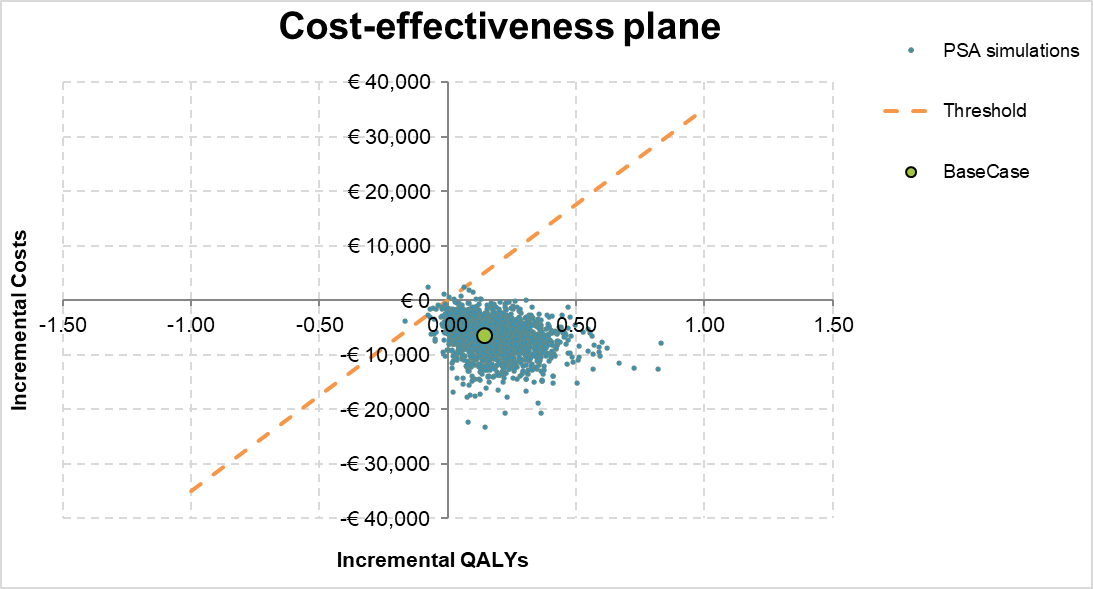


Figure S14: Spain CEP


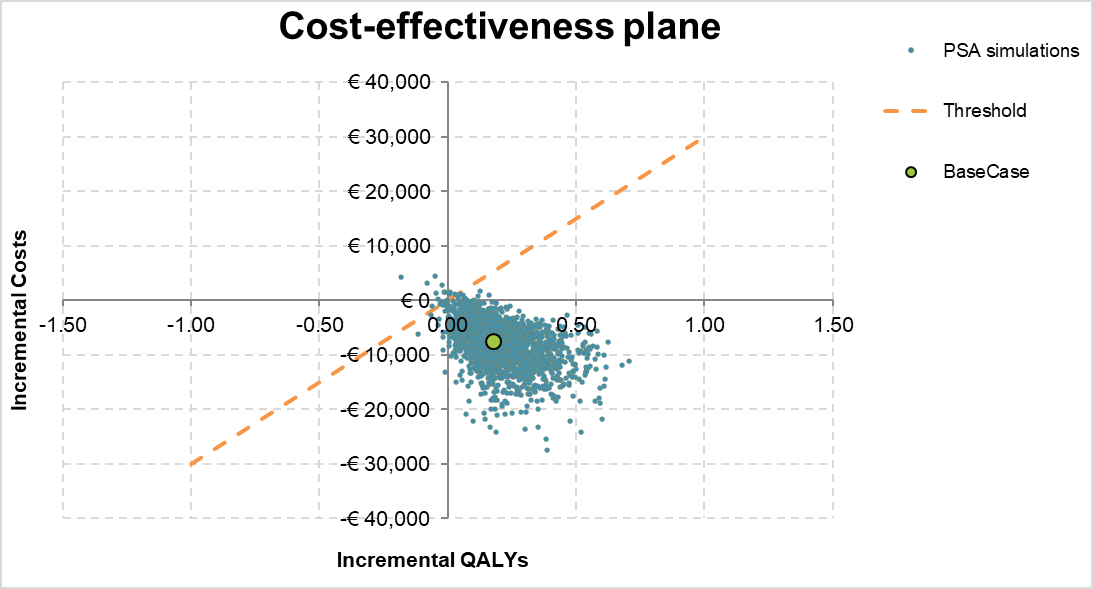


Figure S15: France CEP


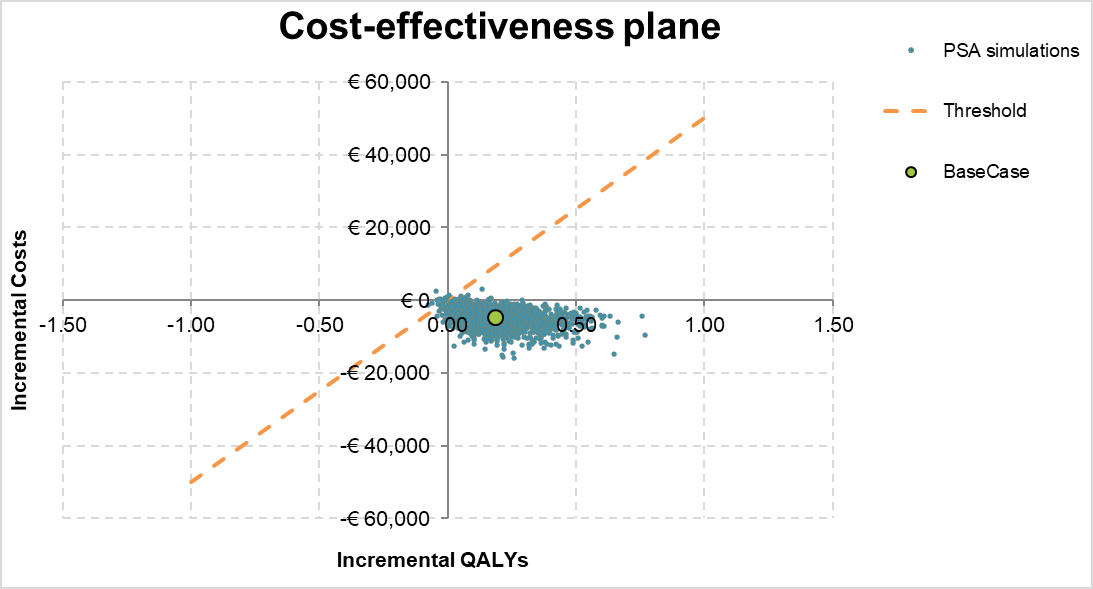


Figure S16: Italy CEP


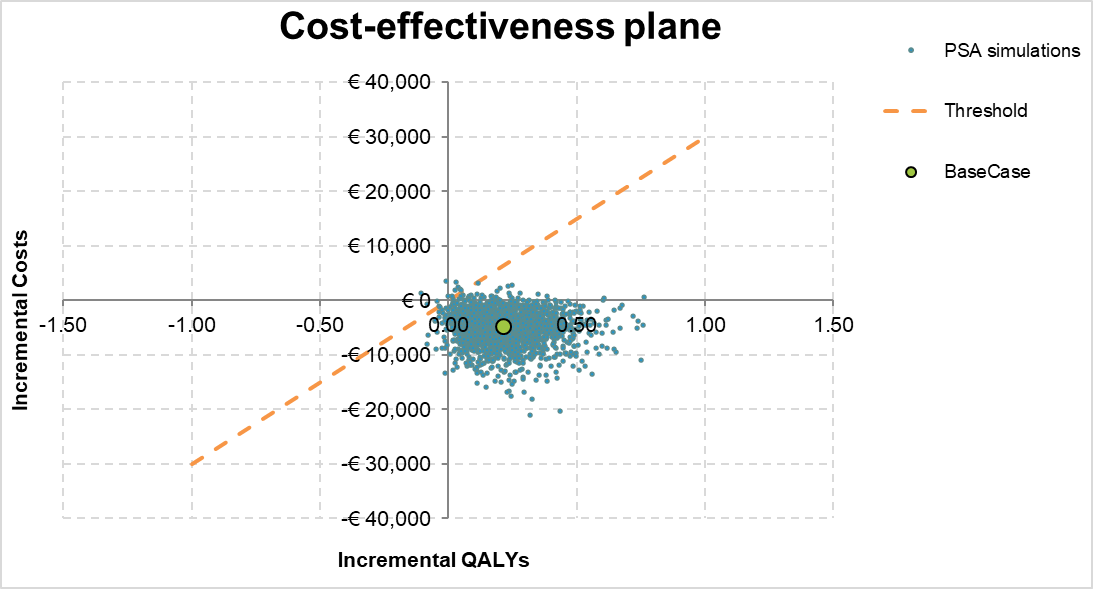


Figure S17: the Netherlands CEP


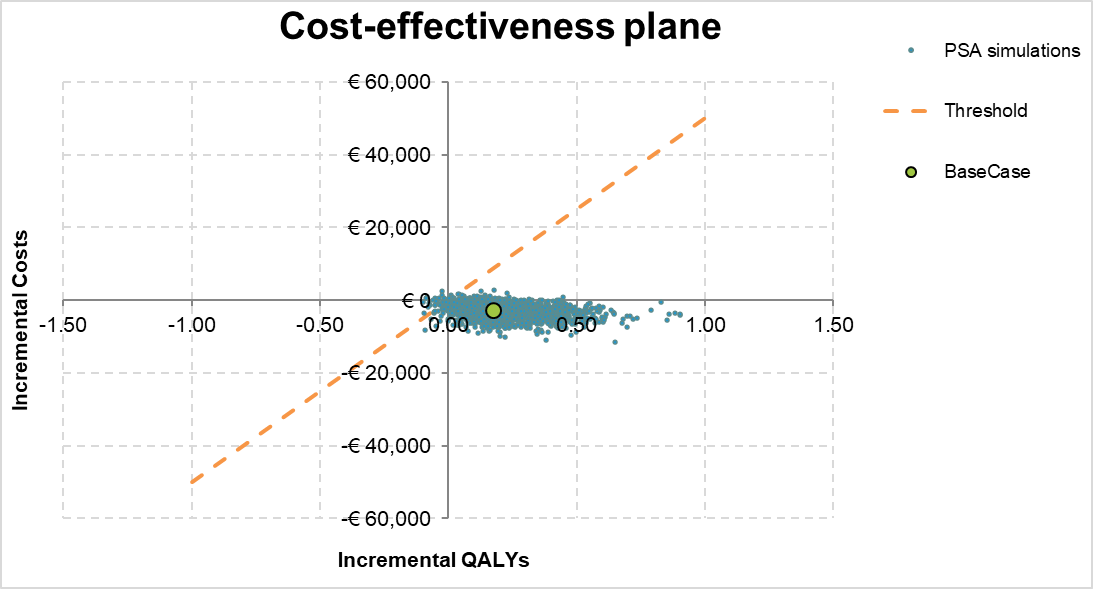


Figure S18: Sweden CEP


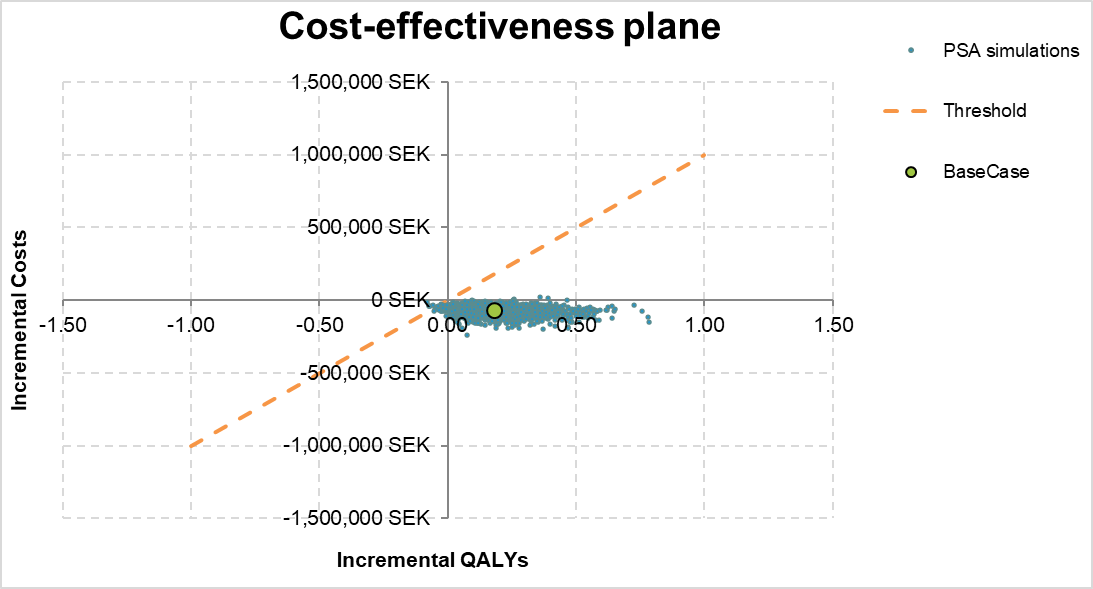


Figure S19: UK CEP

**
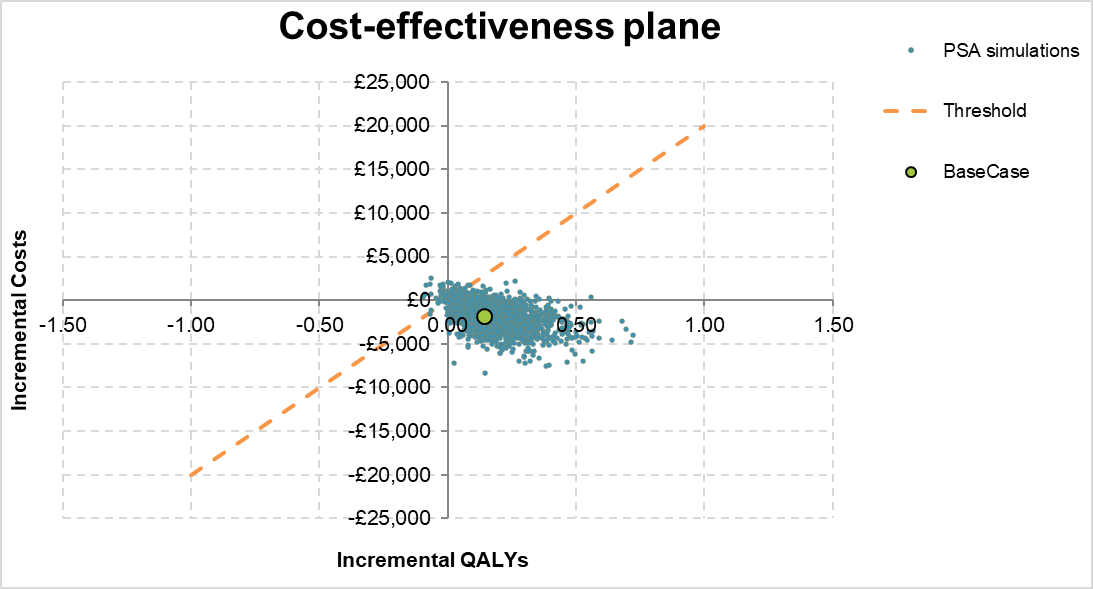
**

### Cost-effectiveness acceptability curves (CEAC)

Figure S20: Belgium CEAC


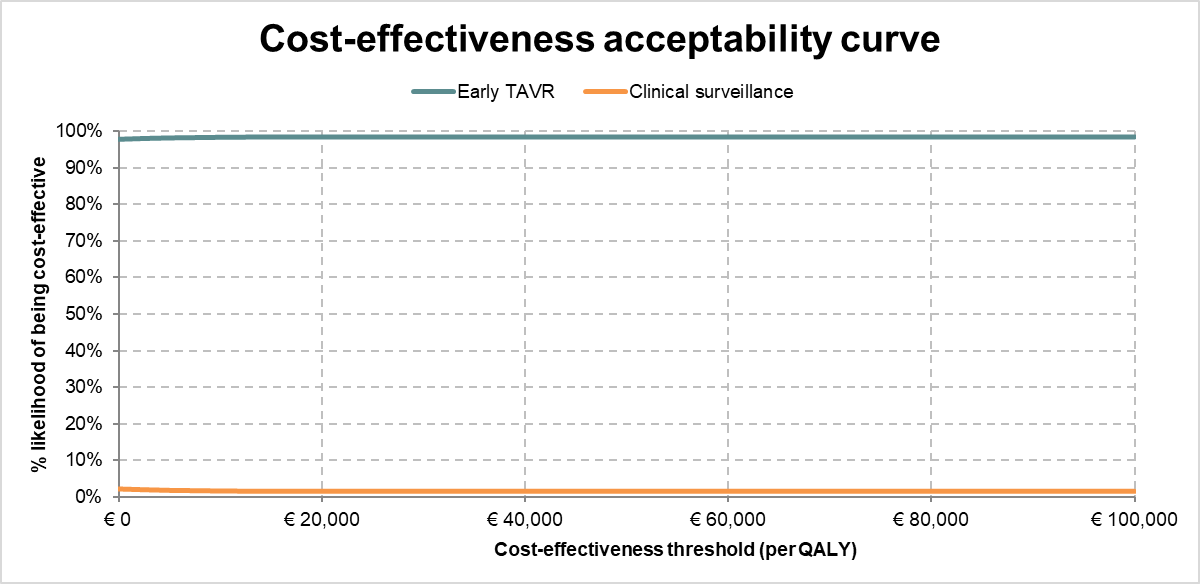


Figure S21: Switzerland CEAC


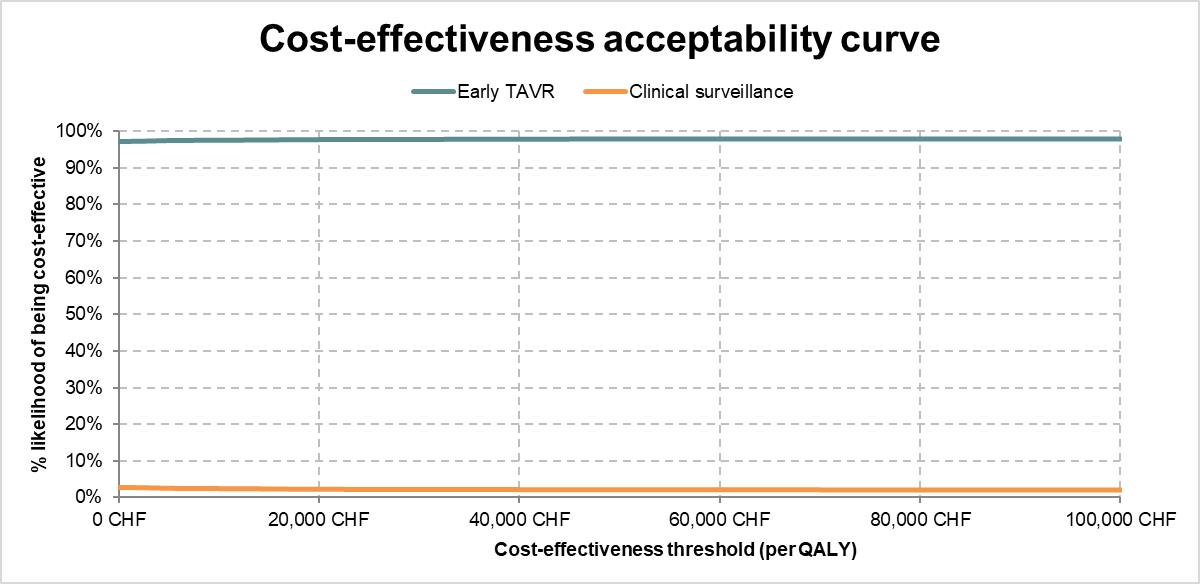


Figure S22: Germany CEAC


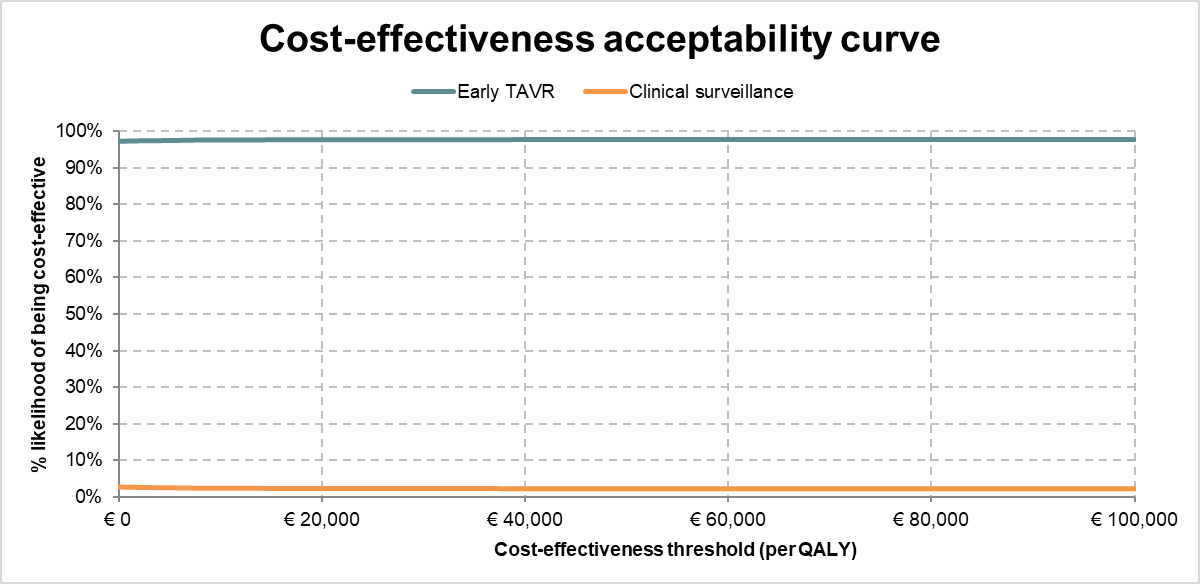


Figure S23: Spain CEAC


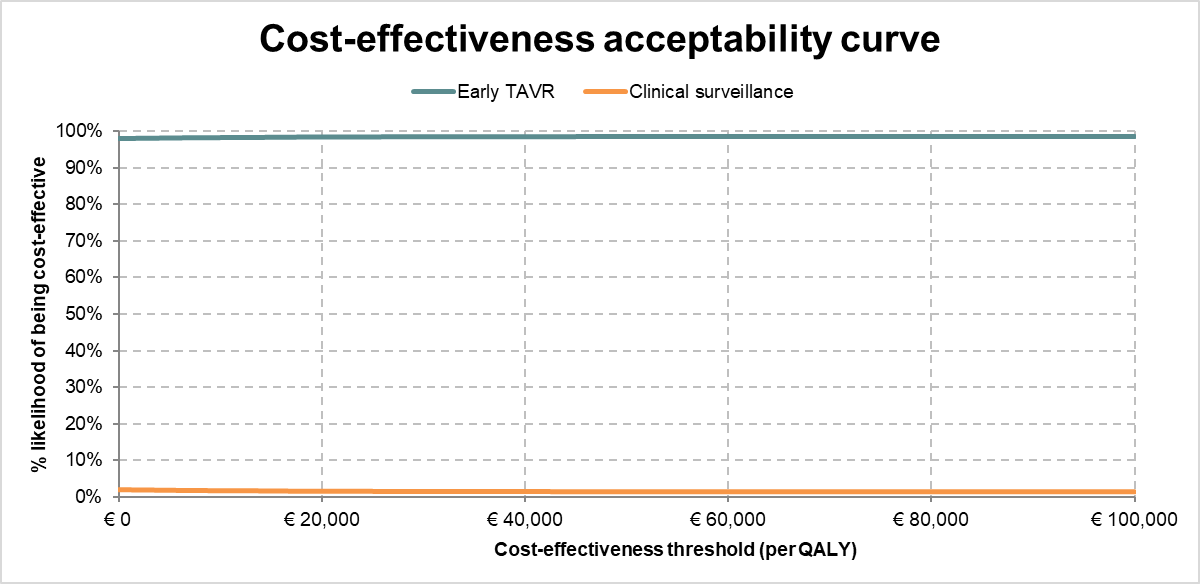


Figure S24: France CEAC


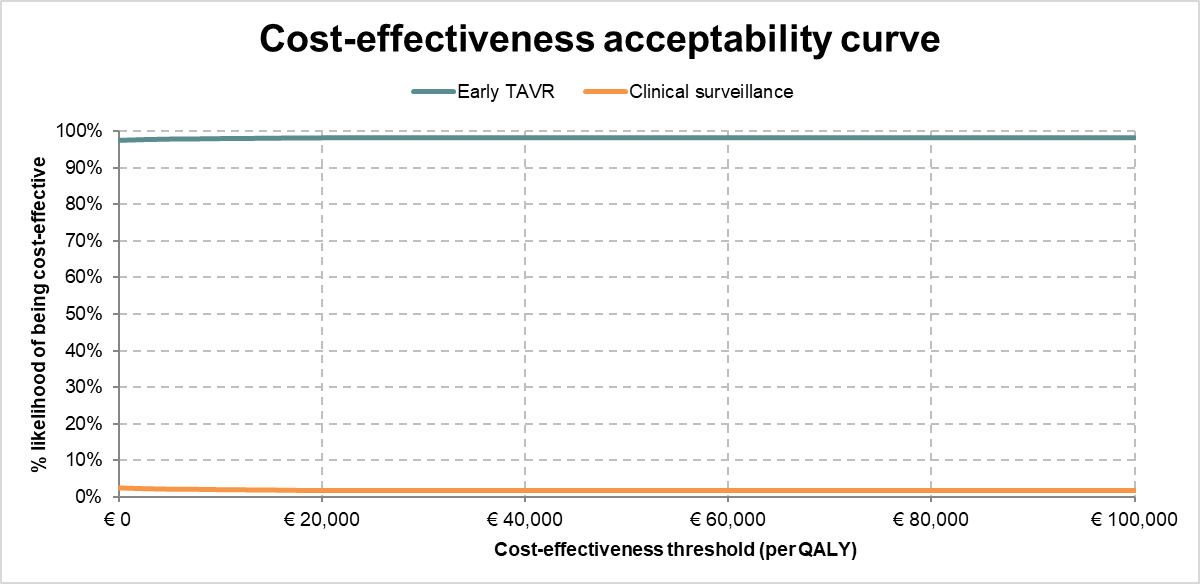


Figure S25: Italy CEAC


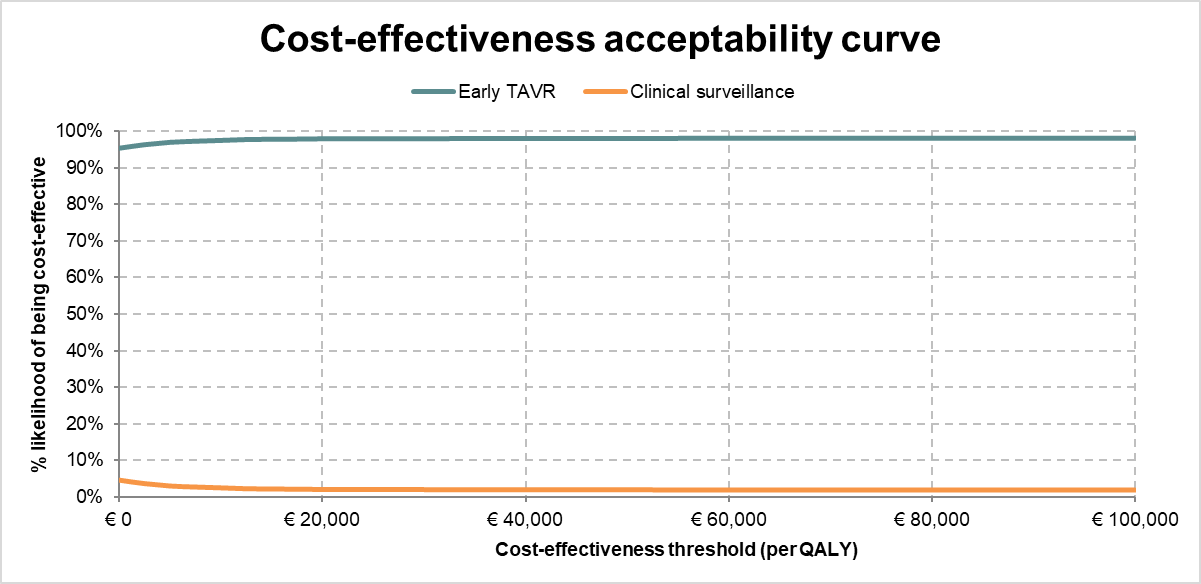


Figure S26: the Netherlands CEAC


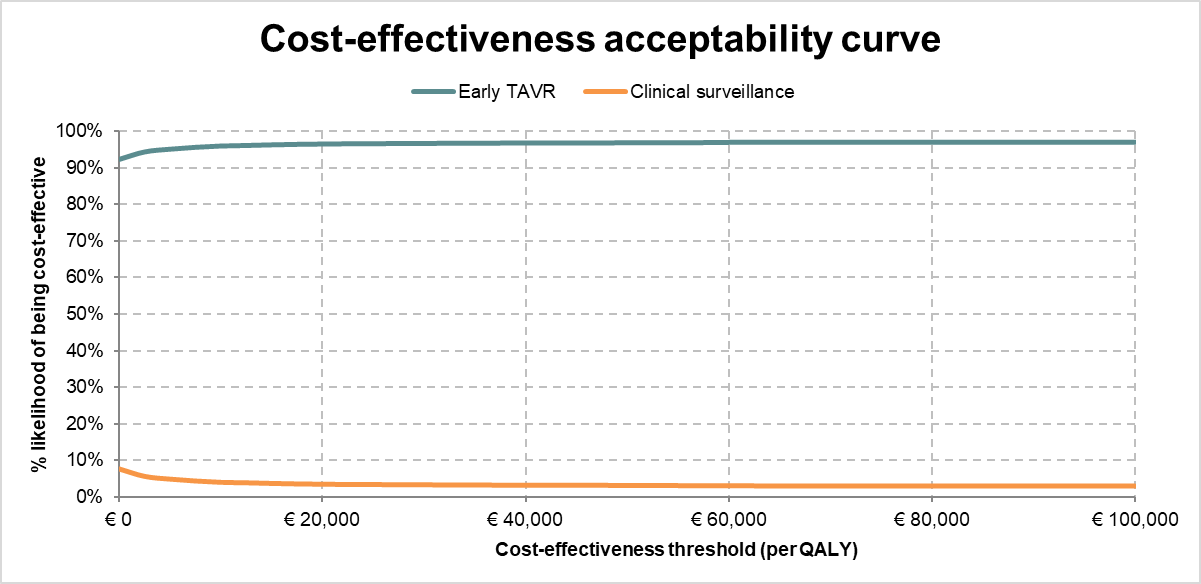


Figure S27: Sweden CEAC


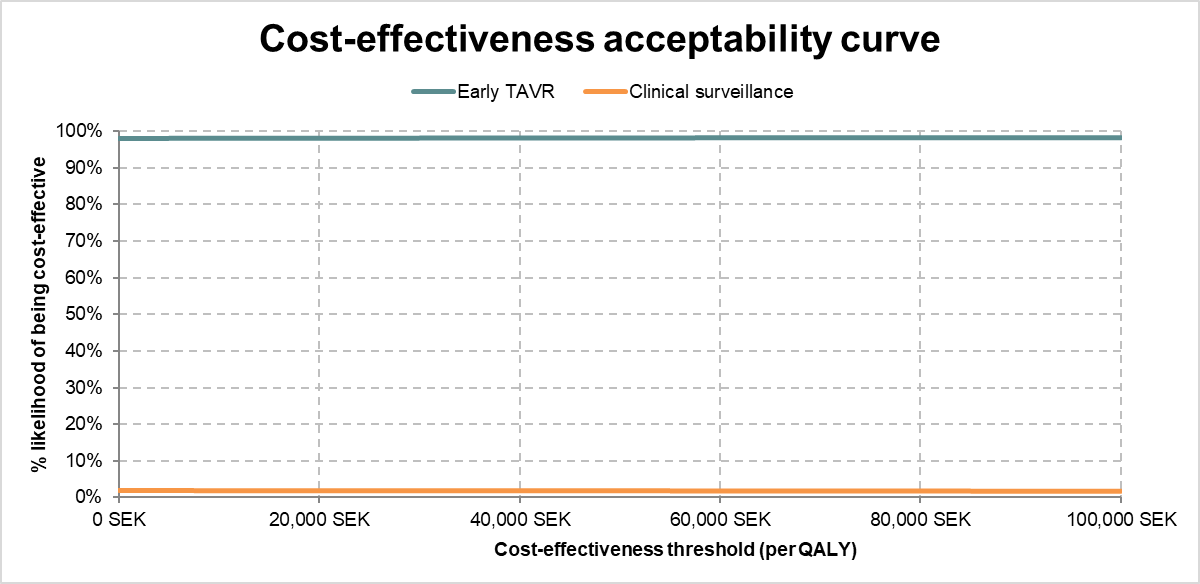


Figure S28: UK CEAC

**
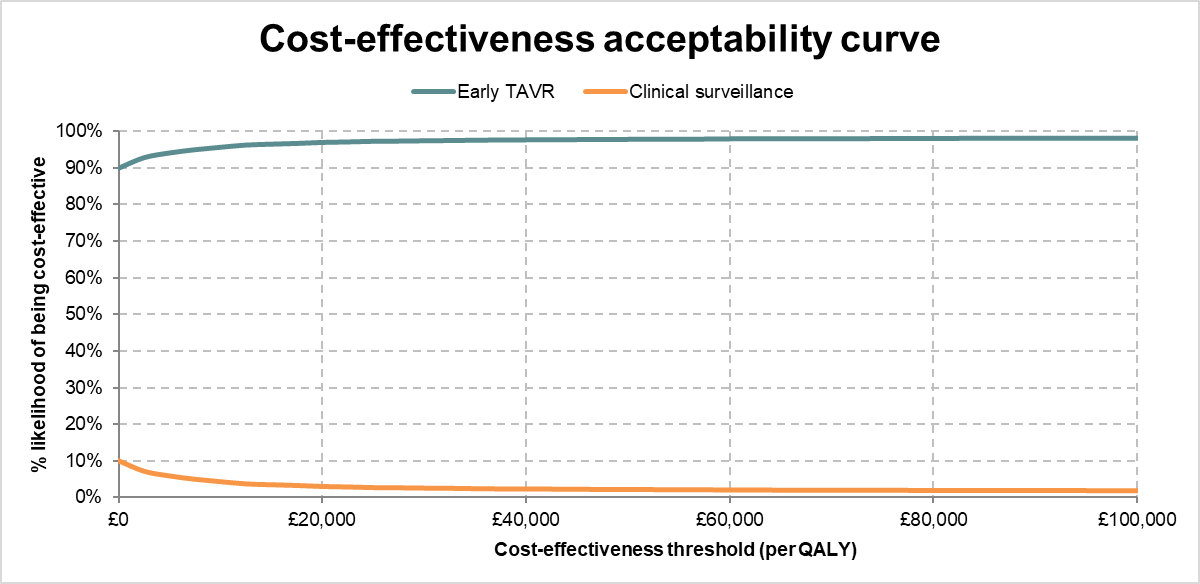
**

### Tornado Diagrams

Figure S29: Belgium tornado diagram


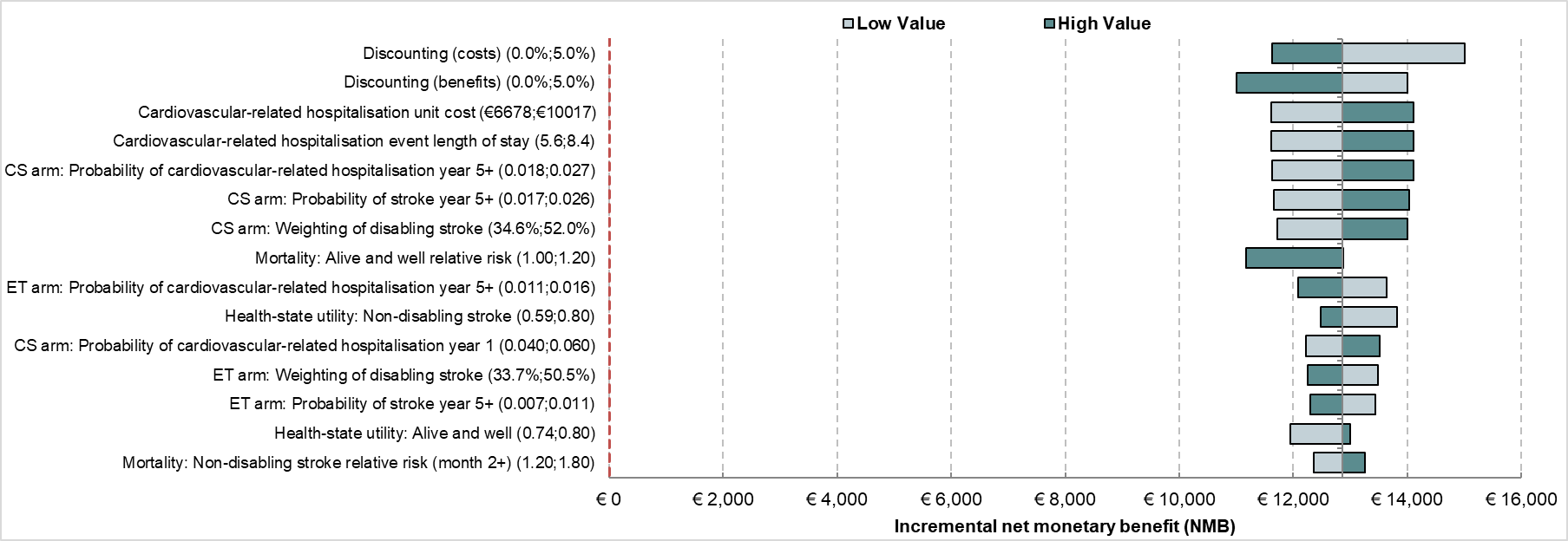


Figure S30: Switzerland tornado diagram


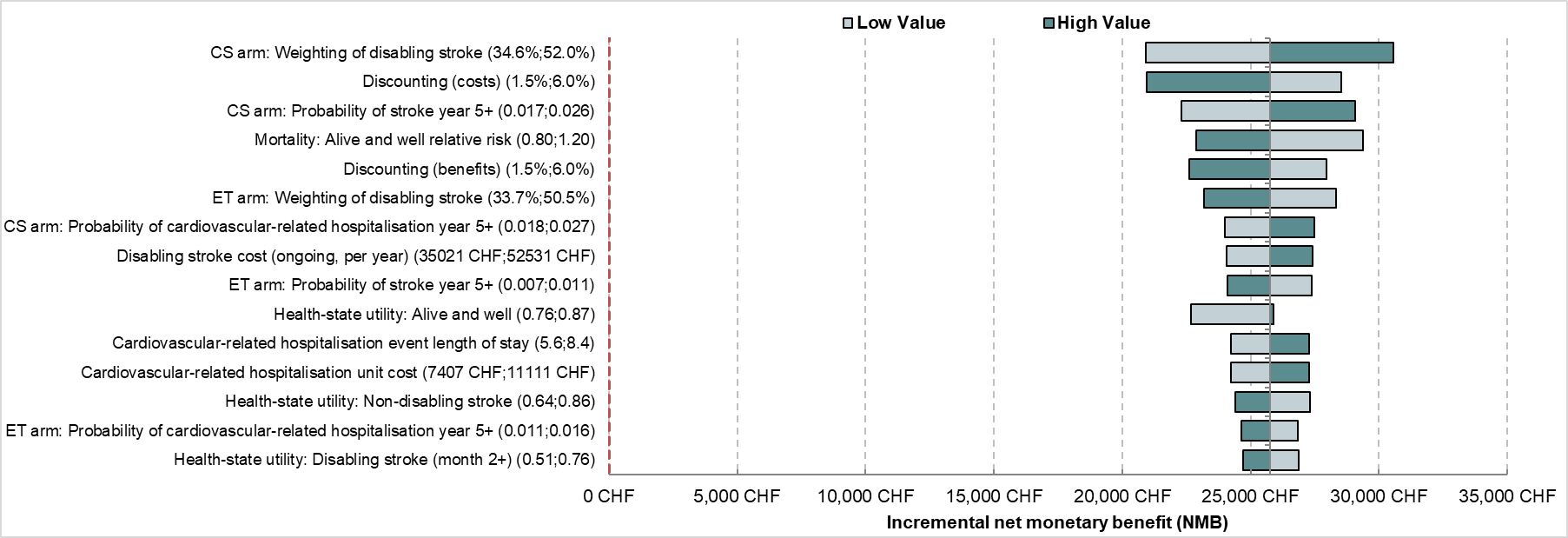


Figure S31: Germany tornado diagram


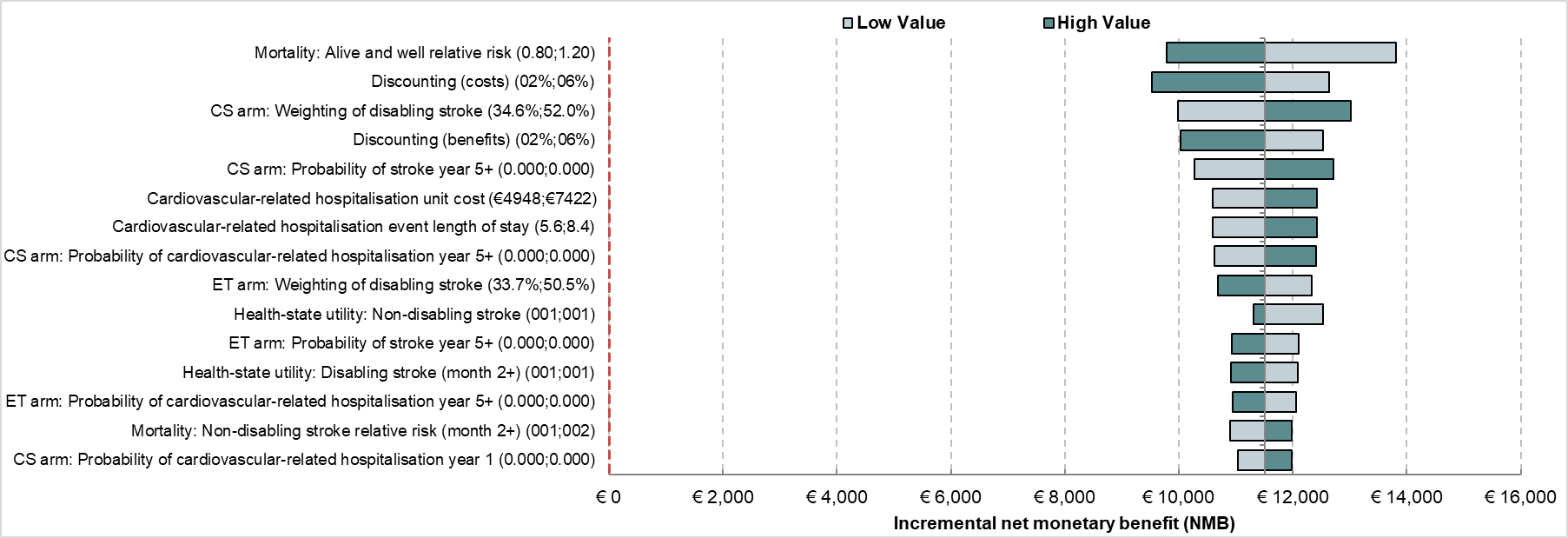


Figure S32: Spain tornado diagram


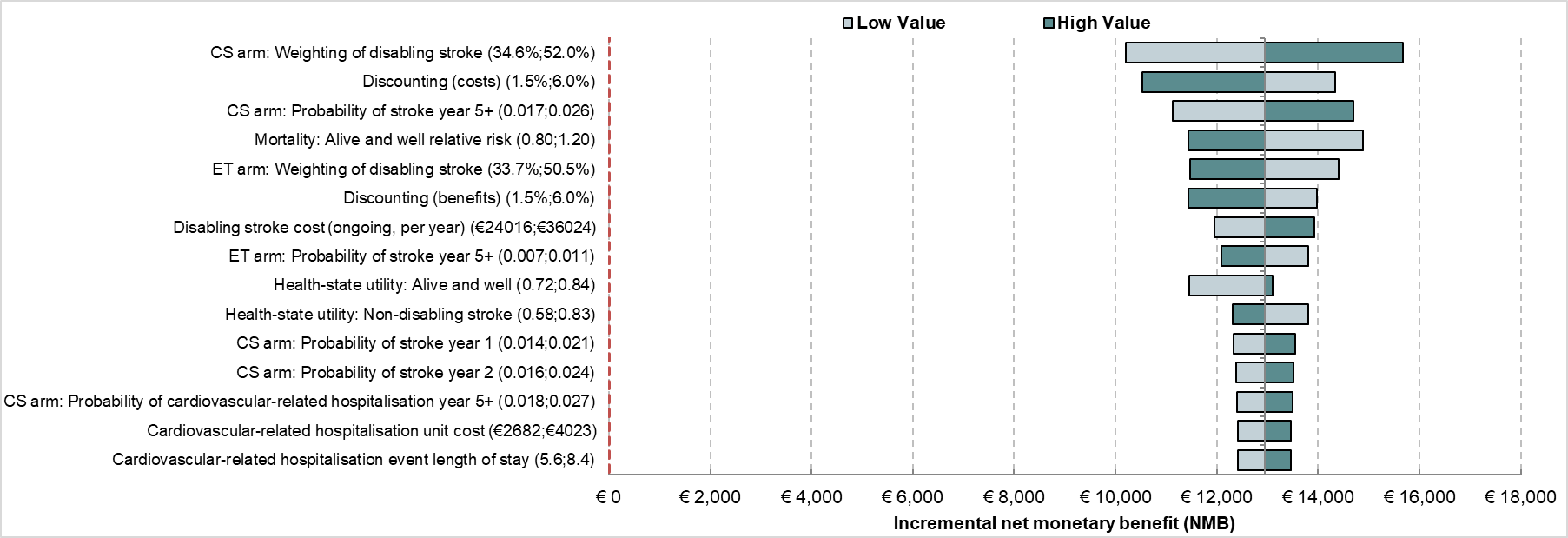


Figure S33: France tornado diagram


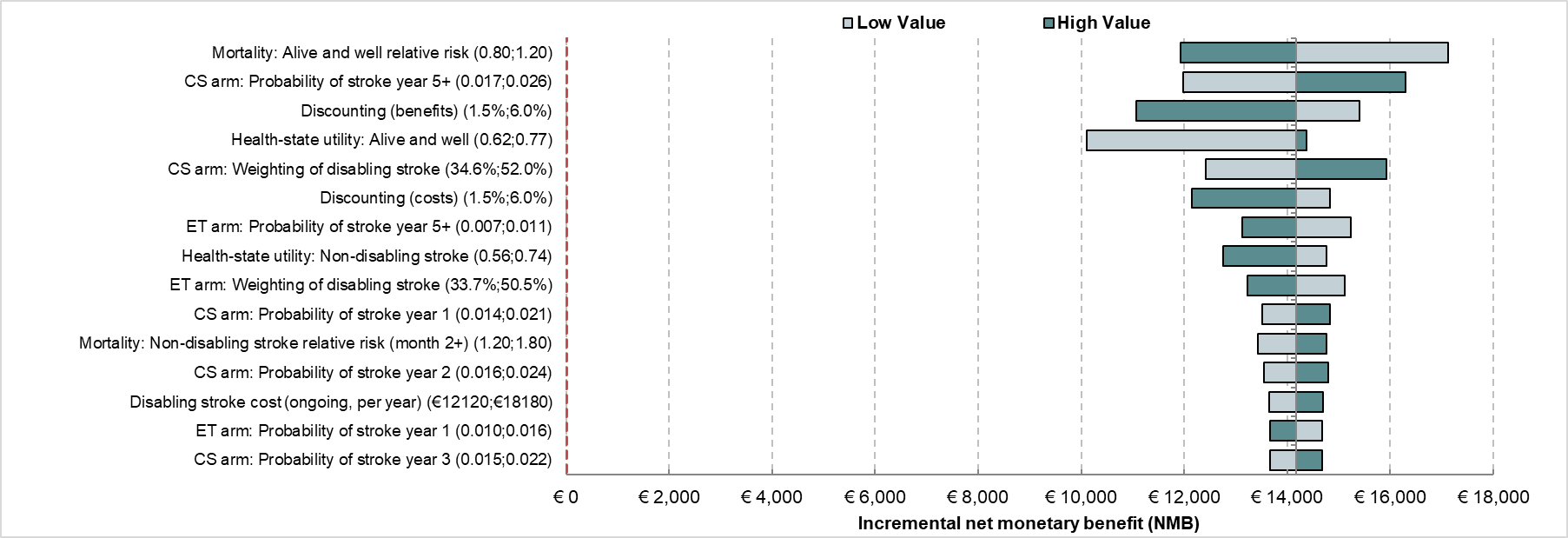


Figure S34: Italy tornado diagram


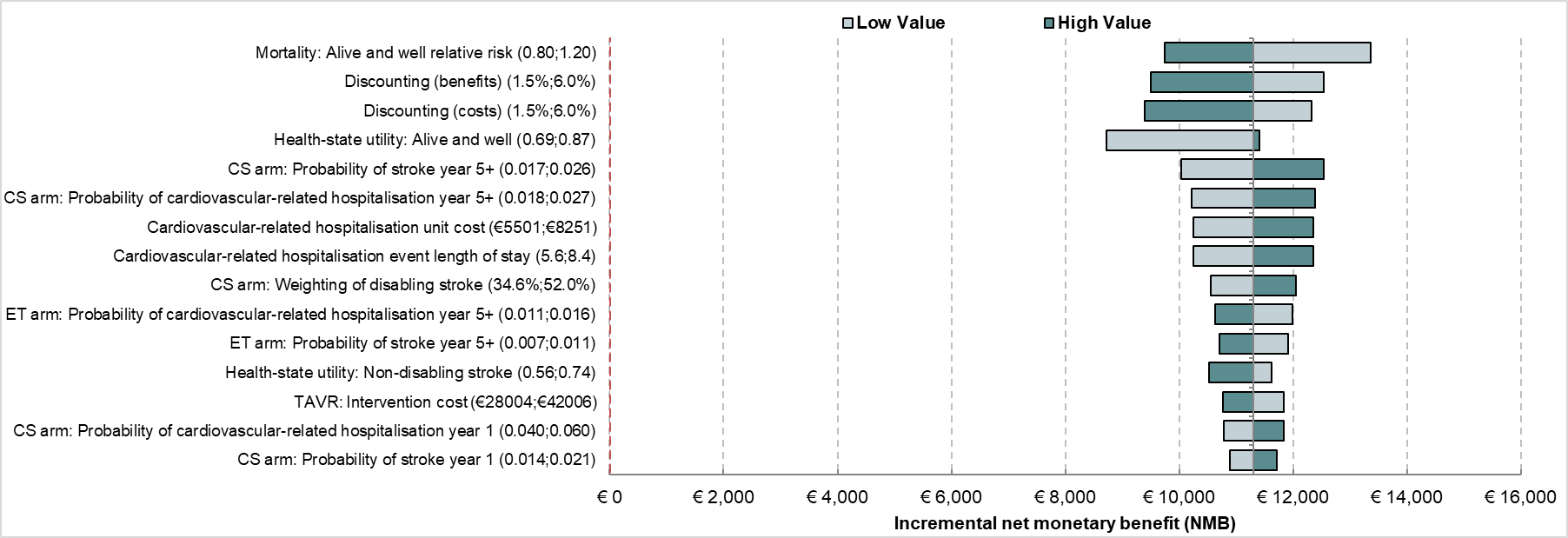


Figure S35: the Netherlands tornado diagram


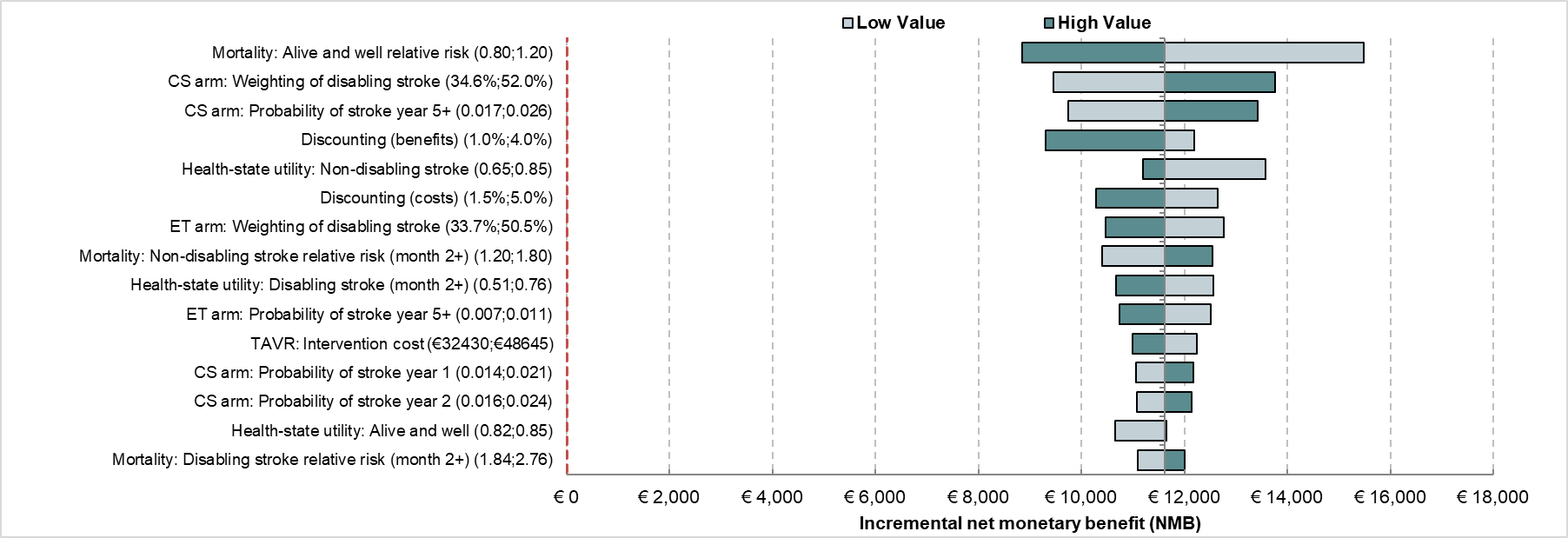


Figure S36: Sweden tornado diagram


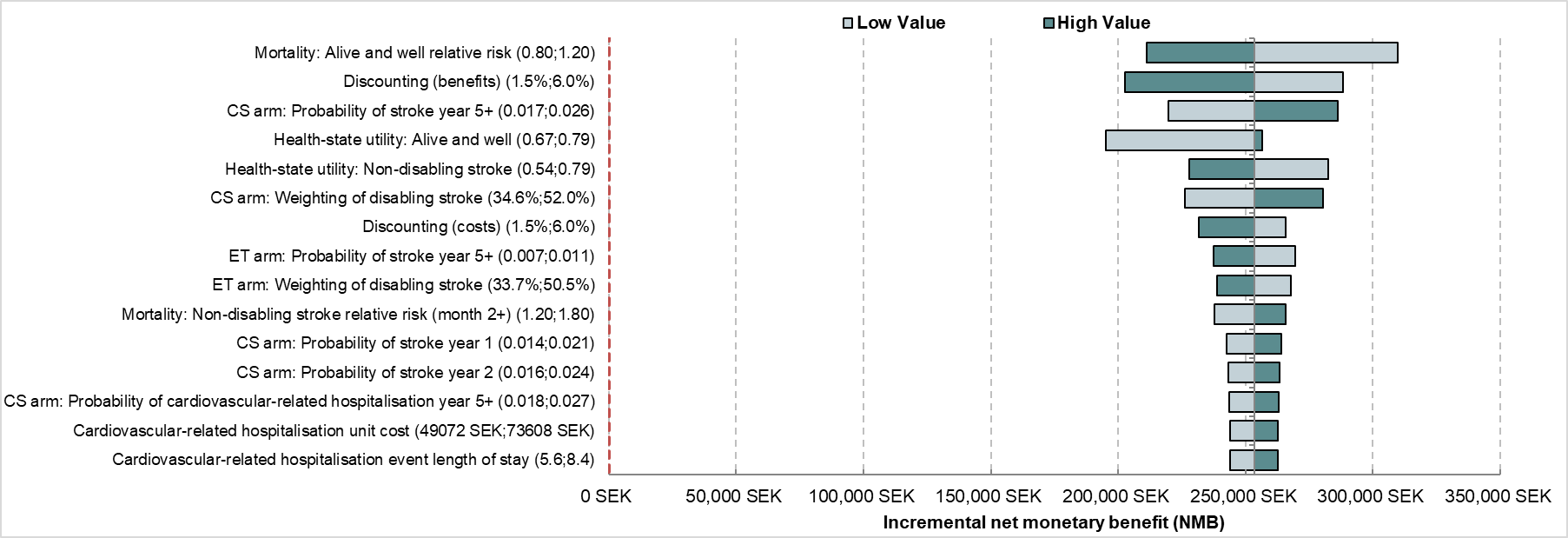


Figure S37: UK tornado diagram


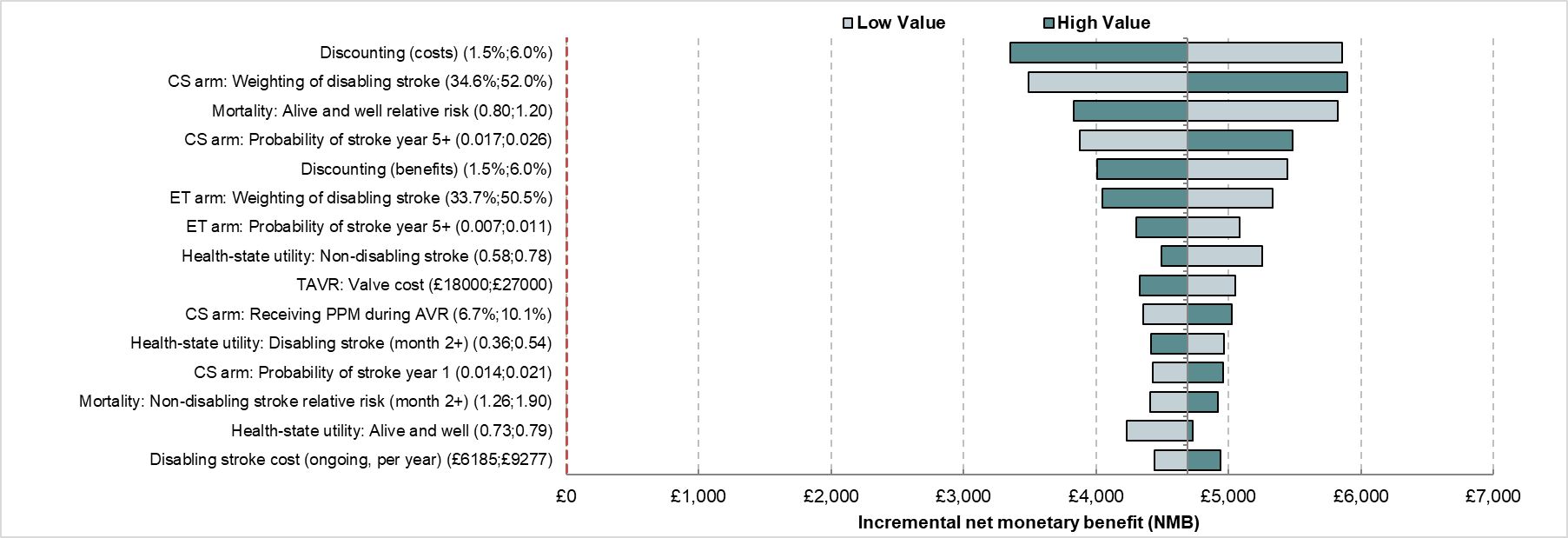


## Supplementary material references

1. Dubois C, Adriaenssens T, Annemans L, Bosmans J, Callebaut B, Candolfi P, Cornelis K, Delbaere A, Green M, Kefer J, Lancellotti P, Rosseel M, Shore J, Van Der Heyden J, Vermeersch S, Wyffels E. Transcatheter aortic valve implantation versus surgical aortic valve replacement in severe aortic stenosis patients at low surgical mortality risk: a cost-effectiveness analysis in Belgium. *Acta Cardiol*.2024;79:46-57

2. Gaziano T, Castellano JM, Dymond A, Looby A, Mealing S, Owen R, Pocock S, González-Juanatey JR, Cordero A, Fernández-Ortiz A, Linhart A, Schiele F, Doehner W, Fernández LO, Fuster V. Cost-effectiveness of the CV-polypill strategy versus standard care for secondary cardiovascular prevention in Spain: an analysis based on the SECURE trial. *The Lancet regional health Europe*.2025;55:101348

3. Hettle R, Wouters H, Ayres J, Gani R, Kelly S, Lion M, Decramer M. Cost-utility analysis of tiotropium versus usual care in patients with COPD in the UK and Belgium. *Respiratory medicine*.2012;106:1722-33

4. Mennini FS, Meucci F, Pesarini G, Vandoni P, Lettino M, Sarmah A, Shore J, Green M, Giardina S. Cost-effectiveness of transcatheter aortic valve implantation versus surgical aortic valve replacement in low surgical risk aortic stenosis patients. *Int J Cardiol*.2022;357:26-32

5. Nilsson K, James S, Angeras O, Backes J, Bjursten H, Candolfi P, Gotberg M, Hagstrom H, Malmberg C, Nielsen NE, Sarmah A, Settergren M, Bromilow T. Cost-effectiveness analysis of transcatheter aortic valve implantation versus surgical aortic valve replacement in patients with severe aortic stenosis at low risk of surgical mortality in Sweden. *Ups J Med Sci*.2025;129:10.48101/ujms.v129.10741

6. Vallejo-Torres L, García-Lorenzo B, Serrano-Aguilar P. Estimating a cost-effectiveness threshold for the Spanish NHS. *Health economics*.2018;27:746-761

7. Wyss CA, Corti R, Nestelberger T, Candolfi P, Delbaere A, Fischer B, Schwenkglenks M, Telser H. Transcatheter aortic valve implantation with SAPIEN 3 versus surgical aortic valve replacement in patients with symptomatic severe aortic stenosis at low risk of surgical mortality: a cost-utility analysis for Switzerland. *Swiss Med Wkly*.2024;154:3558

8. Latimer N. Technical support document 14 (NICE DSU): Survival analysis for economic evaluations alongside clinicial trials - Extrapolation with patient-level data. 2011. <https://www.ncbi.nlm.nih.gov/books/NBK395885/pdf/Bookshelf_NBK395885.pdf>

9. Office for National Statistics. Data from: National Life Tables, England and Wales, period expectation of life, based on data for the years 2017-19. 2025.

10. Human Mortality Database. National life table Spain. Spain Total population. <https://www.mortality.org/Country/Country?cntr=ESP> (Accessed August, 2025)

11. Federal Statistical Office of Germany (Statistisches Bundesamt). National life table Germany (2017/2019). <https://www-genesis.destatis.de/genesis/online?operation=abruftabelleBearbeiten&levelindex=1&levelid=1612797015906&auswahloperation=abruftabelleAuspraegungAuswaehlen&auswahlverzeichnis=ordnungsstruktur&auswahlziel=werteabruf&code=12621-0001&auswahltext=&werteabruf=Value+retrieval#abreadcrumb> (Accessed August, 2025)

12. The National Institute of Statistics and Economic Studies. Mortalité en 2018 - Tableaux de séries longues. <https://www.insee.fr/fr/statistiques/4503155?sommaire=4503178&q=Tables+de+mortalit%C3%A9%20par+sexe%2C%20%C3%A2ge> (Accessed August, 2025)

13. Statistics Sweden. Ettårig livslängdstabell för hela riket efter kön och ålder. År 1960 - 2024. <https://www.statistikdatabasen.scb.se/pxweb/sv/ssd/START__BE__BE0101__BE0101I/LivslangdEttariga/> (Accessed August, 2025)

14. Belgium StatBel. Sterftetafels en levensverwachting [Online]. <https://statbel.fgov.be/sites/default/files/files/documents/bevolking/5.4%20Sterfte%2C%20levensverwachting%20en%20doodsoorzaken/5.4.3%20Sterftetafels%20en%20levensverwachting/sterftetafelsAE.xls>. (Accessed August, 2025)

15. Italian National Institute of Statistics. National life tables Italy (2018). <http://dati.istat.it/Index.aspx?DataSetCode=DCIS_MORTALITA1&Lang=en> (Accessed August, 2025)

16. Statline Netherlands. Levensverwachting; geslacht, leeftijd (per jaar en periode van vijf jaren). <https://opendata.cbs.nl/statline/#/CBS/nl/dataset/37360ned/table?from> (Accessed August, 2025)

17. Généreux P, Schwartz A, Oldemeyer JB, Pibarot P, Cohen DJ, Blanke P, Lindman BR, Babaliaros V, Fearon WF, Daniels DV, Chhatriwalla AK, Kavinsky C, Gada H, Shah P, Szerlip M, Dahle T, Goel K, O'Neill W, Sheth T, Davidson CJ, Makkar RR, Prince H, Zhao Y, Hahn RT, Leipsic J, Redfors B, Pocock SJ, Mack M, Leon MB, Investigators ETT. Transcatheter aortic-valve replacement for asymptomatic severe aortic stenosis. *N Engl J Med*.2025;16:217-227

18. Automeris.io. WebpPlotDigitizer v5. <https://automeris.io/> (Accessed August, 2025)

19. Hernández Alava M , Pudney S WA. Estimating EQ-5D by Age and Sex for the UK. NICE DSU report. 2022. <https://www.sheffield.ac.uk/nice-dsu/methods-development/estimating-eq-5d>

20. Burstrom K, Johannesson M, Diderichsen F. A comparison of individual and social time trade-off values for health states in the general population. *Health Policy*.2006;76:359-70

21. Szende A, Janssen B, Cabases J. Self-reported population health: An international perspective based on EQ-5D. 2014.

22. Mack MJ, Leon MB, Thourani VH, Makkar R, Kodali SK, Russo M, Kapadia SR, Malaisrie SC, Cohen DJ, Pibarot P, Leipsic J, Hahn RT, Blanke P, Williams MR, McCabe JM, Brown DL, Babaliaros V, Goldman S, Szeto WY, Genereux P, Pershad A, Pocock SJ, Alu MC, Webb JG, Smith CR, Investigators P. Transcatheter Aortic-Valve Replacement with a Balloon-Expandable Valve in Low-Risk Patients. *N Engl J Med*.2019;380:1695-1705

23. Bourguignon T, Bouquiaux-Stablo AL, Candolfi P, Mirza A, Loardi C, May MA, El-Khoury R, Marchand M, Aupart M. Very long-term outcomes of the Carpentier-Edwards Perimount valve in aortic position. *Ann Thorac Surg*.2015;99:831-837

24. Baron SJ, Ryan MP, Chikermane SG, Thompson C, Clancy S, Gunnarsson CL. Long-term risk of reintervention after transcatheter aortic valve replacement. *Am Heart J*.2024;267:44-51

25. Edwards Lifesciences. Abstract: Propensity-matched 8-year outcomes following SAVR with novel calcification-resistant versus contemporary tissue bioprostheses. Data on file. presented at: Heart Valve Society April 2025; Cairo, Egypt.

26. Généreux P, Banovic M, Kang DH, Giustino G, Prendergast B, Lindman B, Newby D, Pibarot P, Redfors B, Craig N, Bartunek J, Schwartz A, Seyedin R, Cohen D, Iung B, Leon M, Dweck M. Aortic valve replacement vs clinical surveillance in asymptomatic severe aortic stenosis: A systematic review and meta-analysis. *J Am Coll Cardiol*.2025;85:912-922

27. Généreux P, Pellikka P, Lindman B, Pibarot P, Garcia S, Koulogiannis K, Rodriguez E, Thourani V, Dobbles M, Giustino G, Sharma R, Cohen D, Schwartz A, Leon M, Gillam L. Acute valve syndrome in aortic stenosis. *Struct Heart*.2024;e100377

28. Généreux P, Redfors B, Pibarot P, Lindman BR, Giustino G, Dratch A, Murphy S, Chikermane S, Leon MB, Baron SJ. Health care cost and resource utilization after aortic valve replacement according to the extent of cardiac damage. *Circ Cardiovasc Interv*.2025;18:e014945

29. Makino K, Mudge M, Hill M, Zaunmayr C, Tilden D. Cost-effectiveness of Micra™ VR leadless pacemaker in patients with bradycardia and atrial fibrillation in Australia. *J Arrhythm*.2024;40:1481-1489

30. Mattias Neyt NT, Irina Cleemput. Belgian guidelines for economic evaluations and budget impact analyses. 2025;

31. The Institute for Quality and Efficiency in Health Care (IQWIG). General methods. 2022. <https://www.iqwig.de/methoden/general-methods_version-6-1.pdf>

32. Lopez-Bastida J, Oliva J, Antonanzas F, Garcia-Altes A, Gisbert R, Mar J, Puig-Junoy J. Spanish recommendations on economic evaluation of health technologies. *Eur J Health Econ*.2010;11:513-20

33. Vázquez Rodríguez J, Pinar Bermúdez E, Zamorano JL, Moreu Burgos J, Díaz-Fernández J, García del Blanco B, Sarmah A, Candolfi P, Shore J, Green M. Cost-effectiveness of SAPIEN 3 transcatheter aortic valve implantation in low surgical mortality risk patients in Spain. *REC Interv Cardiol* 2023;5:38-45

34. Haute Autorité de Santé HAS. Choix méthodologiques pour l'évaluation économique à la HAS. Guide méthodologique. 2020. <https://www.has-sante.fr/jcms/r_1499251/en/choices-in-methods-for-economic-evaluation>

35. Farmaco AID. Guidelines for the compilation of the dossier to support the application for reimbursement and pricing of a medicine. 2020. <https://www.aifa.gov.it/en/domanda-rimborsabilita-e-prezzo>

36. Geuzinge HA, El Alili M, Enzing JJ, Huis In 't Veld LM, Knies S, de Wit GA, advisory c. The new Dutch guideline for economic evaluations in healthcare: Taking the societal perspective to the next level. *Value Health*.2025;28:930-935

37. Tandvårds- och läkemedelsförmånsverkets allmänna råd (TLV). Ändring i Tandvårds- och läkemedelsförmånsverkets allmänna råd (TLVAR 2003:2) om ekonomiska utvärderingar. 2017. <https://www.tlv.se/download/18.467926b615d084471ac3230c/1510316374332/TLVAR_2017_1.pdf>

38. NICE. NICE health technology evaluations, the manual: Process and methods [PMG36]. 2022. <https://www.nice.org.uk/process/pmg36/chapter/economic-evaluation-2>

39. Dennis MS, Burn JP, Sandercock PA, Bamford JM, Wade DT, Warlow CP. Long-term survival after first-ever stroke: the Oxfordshire Community Stroke Project. *Stroke*.1993;24:796-800

40. Prencipe M, Culasso F, Rasura M, Anzini A, Beccia M, Cao M, Giubilei F, Fieschi C. Long-term prognosis after a minor stroke: 10-year mortality and major stroke recurrence rates in a hospital-based cohort. *Stroke*.1998;29:126-32

41. Gandjour A, Stock S. A national hypertension treatment program in Germany and its estimated impact on costs, life expectancy, and cost-effectiveness. *Health Policy*.2007;83:257-67

42. de Andres-Nogales F, Alvarez M, de Miquel MA, Segura T, Gil A, Cardona P, Casado MA, Nogueira RG, Davalos A. Cost-effectiveness of mechanical thrombectomy using stent retriever after intravenous tissue plasminogen activator compared with intravenous tissue plasminogen activator alone in the treatment of acute ischaemic stroke due to large vessel occlusion in Spain. *Eur Stroke J*.2017;2:272-284

43. Gilard M, Eltchaninoff H, Iung B, Lefevre T, Spaulding C, Dumonteil N, Mutuon P, Roussel C, Candolfi P, de Pouvourville G, Green M, Shore J. Cost-effectiveness analysis of SAPIEN 3 Transcatheter Aortic Valve Implantation procedure compared with surgery in patients with severe aortic stenosis at low risk of surgical mortality in France. *Value Health*.2022;25:605-613

44. Shah A, Shewale A, Hayes CJ, Martin BC. Cost-effectiveness of oral anticoagulants for ischemic stroke prophylaxis among nonvalvular atrial fibrillation patients. *Stroke*.2016;47:1555-61

45. Ruggeri M, Basile M, Zini A, Mangiafico S, Agostoni EC, Lobotesis K, Saver J, Coretti S, Drago C, Cicchetti A. Cost-effectiveness analysis of mechanical thrombectomy with stent retriever in the treatment of acute ischemic stroke in Italy. *J Med Econ*.2018;21:902-911

46. Geisler BP, Huygens SA, Reardon MJ, Van Mieghem N, Kappetein AP, Osnabrugge RLJ, Pietzsch JB. Cost-effectiveness and projected survival of self-expanding transcatheter versus surgical aortic valve replacement for high risk patients in a European setting: A Dutch analysis based on the CoreValve High Risk trial. *Struct Heart*.2017;1:267-274

47. Myat A, Buckner L, Mouy F, Cockburn J, Baumbach A, Banning AP, Blackman DJ, Curzen N, MacCarthy P, Mullen M, de Belder M, Cox I, Kovac J, Brecker S, Turner M, Khogali S, Malik IS, Alsanjari O, Redwood S, Prendergast B, Trivedi U, Robinson D, Ludman P, de Belder A, Hildick-Smith D. In-hospital stroke after transcatheter aortic valve implantation: A UK observational cohort analysis. *Catheter Cardiovasc Interv*.2021;97:E552-E559

48. NICE. Heart valve disease presenting in adults: investigation and management [NG208]. 2021. <https://www.nice.org.uk/guidance/ng208/resources/heart-valve-disease-presenting-in-adults-investigation-and-management-pdf-66143721453253>

49. Belgian Health Care Knowledge Centre (KCE). Transcatheter aortic valve implantation (TAVI): a Health technology assessment update 2011. <https://kce.fgov.be/sites/default/files/2021-12/kce_163c_tavi_update.pdf>

50. Schuler A, Reuss J, Delorme S, Hagendorff A, Giesel F. [Costs of clinical ultrasound examinations - an economical cost calculation and analysis]. *Ultraschall Med*.2010;31:379-86

51. Wicke FS, Ditscheid B, Breitkreuz T, Glushan A, Lehmann T, Karimova K, Sawicki OA, Vogel M, Freytag A, Beyer M. Clinical and economic outcomes of a collaborative cardiology care program. *Am J Manag Care*.2021;27:e114-e122

52. Kuck KH, Leidl R, Frankenstein L, Wahlers T, Sarmah A, Candolfi P, Shore J, Green M. Cost-effectiveness of SAPIEN 3 Transcatheter Aortic Valve Implantation versus surgical aortic valve replacement in German severe aortic stenosis patients at low surgical mortality risk. *Adv Ther*.2023;40:1031-1046

53. Rodríguez V, Manuel J, Pinar Bermúdez E, Zamorano JL, Moreu Burgos J, Díaz-Fernández J, García del Blanco B, Sarmah A, Candolfi P, Shore J, Green M. Coste-efectividad del implante percutáneo de válvula aórtica con SAPIEN 3 en pacientes con bajo riesgo de mortalidad quirúrgica en España. *REC: Interventional Cardiology*.2023;5:38-45

54. Maladie lA. Health Insurance Public Dataset 2019. <https://www.ameli.fr/assure>

55. Corrao G, Rea F, Iommi M, Lallo A, Fantaci G, Di Martino M, Davoli M, Leoni O, Pompili M, Scondotto S, De Luca G, Carle F, Lorusso S, Giordani C, Di Lenarda A, Maggioni AP, Monitoring, Assessing care Pathways ' working group of the Italian Ministry of H. Cost-effectiveness of outpatient adherence to recommendations for monitoring of patients hospitalized for heart failure. *ESC Heart Fail*.2024;11:2719-2729

56. Marini M, Videsott L, Dalle Fratte CF, Francesconi A, Bonvicini E, Quintarelli S, Martin M, Guarracini F, Coser A, Benetollo PP, Bonmassari R, Boriani G. Economic analysis of remote monitoring in patients with implantable cardioverter defibrillators or cardiac resynchronization therapy defibrillators in the Trento area, Italy. *Front Cardiovasc Med*.2023;10:1151167

57. Lorenzoni V, Pirri S, Turchetti G. Cost-effectiveness of direct non-vitamin K oral anticoagulants versus vitamin K antagonists for the management of patients with non-valvular atrial fibrillation based on available "real-world" evidence: The Italian National Health System perspective. *Clin Drug Investig*.2021;41:255-267

58. Luijten D, van den Hout WB, Boon G, Barco S, Bogaard HJ, Delcroix M, Kreitner KF, Held M, Huisman MV, Jara-Palomares L, Konstantinides SV, Kroft LJM, Mairuhu ATA, Meijboom LJ, van Mens TE, Ninaber MK, Nossent EJ, Pruszczyk P, Valerio L, Vonk Noordegraaf A, Klok FA. Cost-effectiveness of follow-up algorithms for chronic thromboembolic pulmonary hypertension in pulmonary embolism survivors. *ERJ Open Res*.2025;11:

59. Dutch Healthcare Authority. DBC package 2025 integral. <https://puc.overheid.nl/nza/doc/PUC_781989_22/2/> (Accessed 2025,

60. Eerdekens R, Kats S, Grutters JP, Green M, Shore J, Candolfi P, Oortwijn W, Harst PV, Tonino P. Cost-utility analysis of TAVI compared with surgery in patients with severe aortic stenosis at low risk of surgical mortality in the Netherlands. *Cost Eff Resour Alloc*.2024;22:24

61. Sveriges Kommuner och Regioner. Healthcare regions price lists. <https://skr.se/skr/halsasjukvard/ekonomiavgifter/utomlansvardriksavtal/regionernasprislistor.31055.html>

62. NHS England. 2022/23 National Cost Collection data. <https://www.england.nhs.uk/costing-in-the-nhs/national-cost-collection/>

63. NICE. Heart valve disease presenting in adults: investigation and management. [NG208]. Cost-utility analysis: Transcatheter intervention for patients who have operable aortic stenosis. 2021. <https://www.nice.org.uk/guidance/ng208/evidence/tavi-economic-analysis-pdf-10890776557>

64. Shore J, Russell J, Frankenstein L, Candolfi P, Green M. An analysis of the cost-effectiveness of transcatheter mitral valve repair for people with secondary mitral valve regurgitation in the UK. *J Med Econ*.2020;23:1425-1434

65. NHS. National audit of intermediate care summary report. 2017.

66. Dewilde S, Annemans L, Lloyd A, Peeters A, Hemelsoet D, Vandermeeren Y, Desfontaines P, Brouns R, Vanhooren G, Cras P, Michielsens B, Redondo P, Thijs V. The combined impact of dependency on caregivers, disability, and coping strategy on quality of life after ischemic stroke. *Health and quality of life outcomes*.2019;17:31

67. Ali M, MacIsaac R, Quinn TJ, Bath PM, Veenstra DL, Xu Y, Brady MC, Patel A, Lees KR. Dependency and health utilities in stroke: Data to inform cost-effectiveness analyses. *Eur Stroke J*.2017;2:70-76

68. Lanitis T, Cotté FE, Gaudin AF, Kachaner I, Kongnakorn T, Durand-Zaleski I. Stroke prevention in patients with atrial fibrillation in France: comparative cost-effectiveness of new oral anticoagulants (apixaban, dabigatran, and rivaroxaban), warfarin, and aspirin. *J Med Econ*.2014;17:587-98

69. Pradelli L, Calandriello M, Di VR, Bellone M, Tubaro M. Pharmacoeconomic assessment of Apixaban versus standard of care for the prevention of stroke in Italian non-valvular atrial fibrillation patients. *Value Health*.2014;17:A486-7

70. Baeten SA, van Exel NJ, Dirks M, Koopmanschap MA, Dippel DW, Niessen LW. Lifetime health effects and medical costs of integrated stroke services - a non-randomized controlled cluster-trial based life table approach. *Cost Eff Resour Alloc*.2010;8:21

71. Lindgren P, Glader EL, Jönsson B. Utility loss and indirect costs after stroke in Sweden. *European journal of cardiovascular prevention and rehabilitation : official journal of the European Society of Cardiology, Working Groups on Epidemiology & Prevention and Cardiac Rehabilitation and Exercise Physiology*.2008;15:230-3

72. Qureshi AI, Palesch YY. Antihypertensive Treatment of Acute Cerebral Hemorrhage (ATACH) II: design, methods, and rationale. *Neurocritical care*.2011;15:559-76

73. Luengo-Fernandez R, Gray AM, Bull L, Welch S, Cuthbertson F, Rothwell PM. Quality of life after TIA and stroke: ten-year results of the Oxford Vascular Study. *Neurology*.2013;81:1588-95
